# Merging genotyping-by-sequencing data from two *ex situ* collections provides insights on the pea evolutionary history

**DOI:** 10.1093/hr/uhab062

**Published:** 2022-01-19

**Authors:** Stefano Pavan, Chiara Delvento, Nelson Nazzicari, Barbara Ferrari, Nunzio D’Agostino, Francesca Taranto, Concetta Lotti, Luigi Ricciardi, Paolo Annicchiarico

**Affiliations:** 1 Department of Soil, Plant and Food Sciences, University of Bari “Aldo Moro”, Via Amendola 165/A, 70126 Bari, Italy; 2 Council for Agricultural Research and Economics, Research Centre for Animal Production and Aquaculture, viale Piacenza 29, 26900 Lodi, Italy; 3 Department of Agricultural Sciences, University of Naples Federico II, via Università 100, 80055 Portici, Italy; 4 Institute of Biosciences and Bioresources, National Research Council of Italy, Via Amendola 165/A, 70126 Bari, Italy; 5 Department of Agriculture, Food, Natural Resources and Engineering, University of Foggia, Via Napoli 25, 71100 Foggia, Italy

## Abstract

Pea (*Pisum sativum* L. subsp. *sativum*) is one of the oldest domesticated species and a widely cultivated legume. In this study, we combined next generation sequencing (NGS) data referring to two genotyping-by-sequencing (GBS) libraries, each one prepared from a different *Pisum* germplasm collection. The selection of single nucleotide polymorphism (SNP) loci called in both germplasm collections caused some loss of information; however, this did not prevent the obtainment of one of the largest datasets ever used to explore pea biodiversity, consisting of 652 accessions and 22 127 markers. The analysis of population structure reflected genetic variation based on geographic patterns and allowed the definition of a model for the expansion of pea cultivation from the domestication centre to other regions of the world. In genetically distinct populations, the average decay of linkage disequilibrium (LD) ranged from a few bases to hundreds of kilobases, thus indicating different evolutionary histories leading to their diversification. Genome-wide scans resulted in the identification of putative selective sweeps associated with domestication and breeding, including genes known to regulate shoot branching, cotyledon colour and resistance to lodging, and the correct mapping of two Mendelian genes. In addition to providing information of major interest for fundamental and applied research on pea, our work describes the first successful example of integration of different GBS datasets generated from *ex situ* collections – a process of potential interest for a variety of purposes, including conservation genetics, genome-wide association studies, and breeding.

## Introduction

Pea (*Pisum sativum* L. subsp. *sativum*, hereinafter referred to as *P. s. sativum*) is the fourth most important legume in the world, with a production exceeding 36 Mt in 2019 (http://www.fao.org/faostat/). Pea seeds are a strategic commodity for global food security, as they represent a rich and affordable source of proteins for human nutrition and livestock feeding. In addition, the inclusion of pea in crop rotations allows the fixation of atmospheric nitrogen and leads to beneficial effects on soil physical properties [1]. Positive assets of pea relative to other grain legumes are a trend towards higher production of grain and feed energy per unit area and a relatively high rate of genetic yield gain [2].

According to a widely accepted classification, the genus *Pisum* includes two wild taxa: *Pisum fulvum*, endemic to Jordan, Syria, Lebanon, Israel and Palestine, and *Pisum sativum* subsp. *elatius* (hereinafter referred to as *P. s. elatius*), the wild pea ancestor, reported in the Mediterranean Basin, Europe, and the Caspian Region [3, 4]. Archaeological records indicate that pea domestication occurred in farming settlements of the Fertile Crescent about 9000 years BP, thus making pea one of the first crops in the agricultural history [5]. A second and more recent domestication event is thought to be at the origin of a minor taxon, *P. sativum* L. subsp. *abyssinicum* (Abyssinian pea), presently grown in Ethiopia and Yemen [4, 6].

With the aim to preserve and exploit *Pisum* biodiversity, several *ex situ* collections have been established around the world, together encompassing more than 55 000 accessions [7]. From there, smaller collections were selected to summarize genetic variation and fuel research in genetics and breeding. Among them, the Pea Single Plant Plus (PSPP) + *P. fulvum* collection, owned by the United States Department of Agriculture (USDA), includes 431 *P. sativum* and 25 *P. fulvum* accessions selected on the basis of geographic and morphological diversity [8]. The collection owned by the Italian Council for Agricultural Research and Economics (CREA) includes 234 *P. sativum* accessions selected on the ground of indications provided by curators of national or international gene banks, in an effort to maximize the morpho-physiological variation from each country represented in the collection [9].

Genotyping methods based on reduced representation DNA libraries, such as genotyping-by-sequencing (GBS), allow for the cost-effective application of next generation sequencing (NGS) to explore genetic variation of germplasm collections [10, 11, 12]. GBS is particularly suitable for species with large genomes, such as pea (~ 4.45 Gb), for which whole genome resequencing of a large number of individuals would be economically burdensome [13, 14]. Recently, the PSPP + *P. fulvum* collection was subjected to GBS, leading to the identification of SNPs used to study pea genetic structure [8]. Based on principal component analysis (PCA), clear genetic differentiation was found between wild and cultivated *Pisum* accessions; in addition, within *P. s. sativum*, a peculiar gene pool was associated with landraces originating from Central Asia, known as “Afghanistan” landraces [8]. Genetic divergence of “Afghanistan” landraces was also reported by additional studies, and explained as the result of genetic drift and adaptation to a specific environmental niche [4, 9, 15, 16].

**Table 1:** Breadth of coverage associated with the PSPP + *P. fulvum* and CREA GBS libraries. Information is provided on the number of nucleotide sites with at least 1x depth on average over all samples, and the percentage of these sites shared with the mate library

**Chromosome**	**CREA**		**USDA**	
	**Sites covered**	**Sites shared (%)**	**Sites Covered**	**Sites shared (%)**
chr1LG6	1 653 095	35.21	942 482	61.76
chr2LG1	1 673 759	33.86	972 034	58.30
chr3LG5	1 853 831	34.56	1 054 515	60.75
chr4LG4	1 875 339	35.01	1 085 451	60.48
chr5LG3	2 506 131	35.45	1 461 342	60.79
chr6LG2	2 059 467	35.75	1 183 290	62.23
chr7LG7	2 127 700	35.42	1 218 009	61.87
Average	1 964 189	35.04	1 131 018	60.88

NGS data from the PSPP + *P. fulvum* GBS library were made publicly available to provide a resource for the scientific community and breeders [8]. In the present study, we merged these data with those from a newly developed GBS library, referring to the CREA pea collection. The resulting large SNP dataset, mapped onto the recently released pea genome assembly [13], was used with the purpose to study the pea genetic structure, identify genomic signatures of domestication and breeding, and carry out demo genome-wide association studies.

## Results

### Obtainment of a SNP panel by library data merging

Sequencing of an *ApeK*I GBS library prepared from the CREA collection generated about 3.6 million reads/sample. Raw FASTQ files were deposited at the Sequence Read Archive (SRA) database under the BioProject identification number PRJNA719084. Genomic windows containing sites sequenced with at least 1x average depth from the CREA library and/or the PSPP + *P. fulvum* library, the latter also obtained with the *ApeK*I restriction enzyme [8], are shown in Supplementary Fig. S1. Considering nucleotide sites with at least 1x average depth, the CREA library was associated with 74% higher breadth of coverage than the PSPP + *P. fulvum* library. In addition, sites shared with the mate library were 60.9% for the PSPP + *P. fulvum* library and 35.1% for the CREA library (Table 1). The SNP calling pipeline yielded a total of 307 063 variants. Filtering for MAF, call rate and inbreeding coefficient resulted in the selection of 22 127 SNP loci called in both germplasm collections, with a transition/transversion rate of 1.98. Most of the SNPs (19 130) mapped in genic regions, of which 10 226 in coding sequences and 8 904 in introns or UTRs. Sanger sequencing of 12 PCR fragments mapping on the seven pea chromosomes confirmed, with no exception, 20 random polymorphisms identified after the SNP call and filtering procedures (Supplementary Table S1).

Thirty-five accessions displaying more than 30% of missing data were excluded from downstream analyses, leading to a final dataset of 652 accessions (Supplementary Table S2). Available information on the origin of these accessions is shown in Supplementary Table S2.

**Figure 1:**
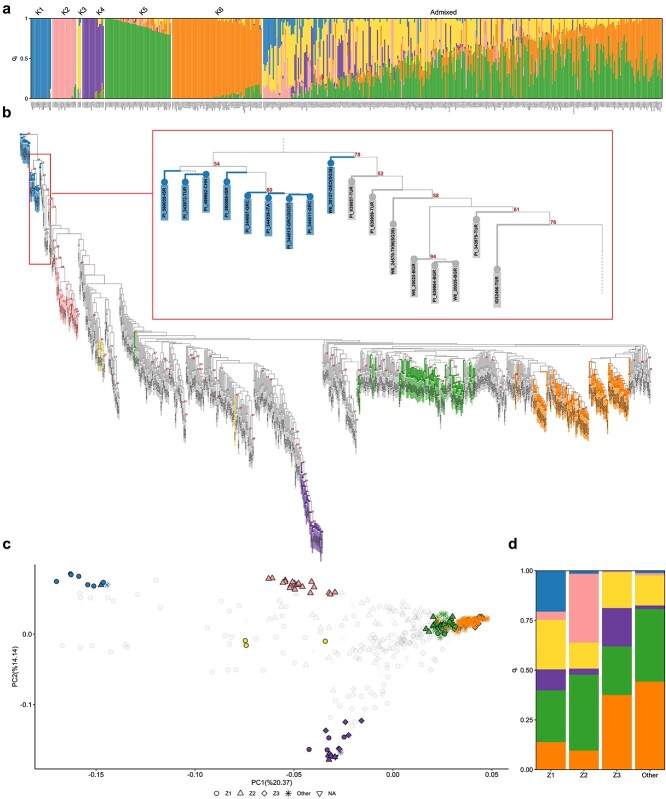
Results from the analysis of pea genetic structure. (a) Bar plot for a model with six ancestral populations (K). Each vertical bar refers to an individual accession. The length and the colour of segments in each bar represent the proportion of the genome (q_i_) associated with each population. (b) Neighbour joining phylogeny, using *P. fulvum* as outgroup. Bootstrap values >50 are presented. Accessions assigned to populations K1-K6 are coloured in accordance with Fig. 1a, while admixed individuals are coloured in grey. A detail of the tree is enlarged to show divergence of accessions from Turkey, Bulgaria, and Turkmenistan [1] from K1. (c) Scatter plot for *P. sativum* diversity explained by the first two principal components (PCs). Samples are coloured according to panel b. Symbols indicate the origin of the accessions: 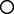 = Z1 (Southeastern Balkans, Northern Africa, Western Asia); Δ = Z2 (Central Asia); 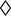 = Z3 (Northern/Western Balkans, Central Europe, France); 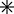  = other regions; 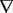  = NA. (d) Stacked bar plot indicating the ancestry of admixed individuals identified by structure analysis for K = 6, in relation to their origin in Z1, Z2, Z3, or other regions of the world.

### Identification of genetic redundancy among accessions

Based on the pairwise allelic identity by state (IBS) threshold of 0.99, 38 groups of putatively duplicated accessions (duplication groups or DGs) were identified, together encompassing 91 accessions. With a few exceptions, accessions in the same DG were referable to the same or neighbouring countries (Supplementary Table S3). The rate of accessions having at least one putative duplicate within the same collection was 17% for the PSPP + *P. fulvum* collection and 3% for CREA collection.

A lower IBS threshold (0.95) was used to obtain groups of closely related accessions (similarity groups or SGs). In this case, 212 accessions were assigned to 81 SGs. As closely related individuals might introduce bias in genetic structure analysis [17], only one accession for each SG, displaying the highest SNP call rate, was retained (Supplementary Table S4), leading to a dataset of 521 accessions.

### Genetic structure

The application of the STRUCTURE algorithm [18] and the ΔK method [19] indicated that models with two, three and six ancestral populations (K) were appropriate to describe the pea genetic structure (Supplementary Fig. S2). At the finest level of resolution, (K = 6, Fig. 1a), 188 individuals were assigned to a specific population, as they were characterized by a fraction of the genome having ancestry in that population (the membership coefficient parameter q_i_) higher than 0.8, whereas the remaining 333 accessions were classified as admixed. The first population (K1) grouped wild germplasm of *P. fulvum* [8] and *P. s. elatius* [8], together with one accession with unspecified taxonomy (PI 499982); K2 grouped 20 accessions from different countries of Central Asia (Afghanistan, China, India, Iran, Nepal and Pakistan); K3 grouped two accessions from Georgia and one from Russia; K4 grouped 18 accessions, mainly from the Balkan Peninsula, Central Europe, and France, among which the frost tolerant varieties Champagne, Cote d’Or, Haute Loire, Picar, and Pleven 10 (the latter corresponding to the accession PI 639981) [20, 21, 22]; K5 grouped 55 accessions, mainly from Ethiopia and India; K6 grouped 75 accessions, mainly improved cultivars developed in U.S. and Europe. After excluding *P. fulvum* accessions from K1, the pairwise genetic distance among the six *P. sativum* populations, expressed as the Wright’s F_ST_ coefficient, ranged from 0.12 (K5 vs. K6) to 0.48 (K1 vs. K4) (Table 2). Nucleotide diversity (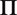) calculated for the populations from K1 to K6 were 0.102, 0.118, 0.104, 0.074, 0.095, and 0.079, respectively.

The three-population model implied the merging of K1 with K2, and K5 with K6 (Supplementary Fig. S3). The two-population scenario resulted in the merging of K5 with K6 on the one hand, and K1, K2, K3 and K4 on the other hand (Supplementary Fig. S3).

Hierarchical clustering and principal component analysis (PCA) were used to complement STRUCTURE results and define relationships among individual accessions. The first approach, using *P. fulvum* as outgroup, resulted in a phylogeny in which eight accessions from Turkey (4), Bulgaria (3), and Turkmenistan (1) were the first to diverge from the K1 group. In addition, K2 and K4 accessions were included into two well-defined monophyletic clades (Fig. 1b).

PCA highlighted, on its main axis (PC1), a pattern of variation from the wild K1 group to cultivated germplasm. Specifically, the closest accessions to K1 grouped in K3, or displayed admixture dominated by K1 and K3 (Fig. 1c and Supplementary Fig. S4). With a few exceptions, they originated from a zone (Z1) encompassing Southeastern Balkans, Northern Africa, and Western Asia (including the Middle East and the Caspian region) (Fig. 1c and Supplementary Table S5). Accessions from Central Asia (zone Z2), among which those grouping in K2, started to be significantly represented at an intermediate distance from K1, together with accessions from Northern and Western Balkans, Central Europe, and France (zone Z3), several of which grouped in K4. Finally, accessions from other regions of the world, including those grouping in K5 and K6, were in a distal position with respect to K1. PC2 and PC3 captured variation leading to the differentiation of accessions included in K4 and K2, respectively (Fig. 1c and Supplementary Fig. S5).

The ancestry of the 333 admixed accessions identified for K = 6 exhibited a clear geographic pattern in relation to the zones defined by PCA (Fig. 1d). Specifically, significant wild ancestry (K1) only occurred in admixed individuals from Z1. The K2 ancestry, occurring in admixed individuals from Z1 and Z2, was negligible in admixed samples from Z3 and other regions of the world; similarly, the K4 ancestry, significant in admixed individuals from Z1 and Z3, was poorly represented in admixed individuals from Z2 and other regions of the world.

### Evolutionary signatures in the pea genome

**Figure 2:**
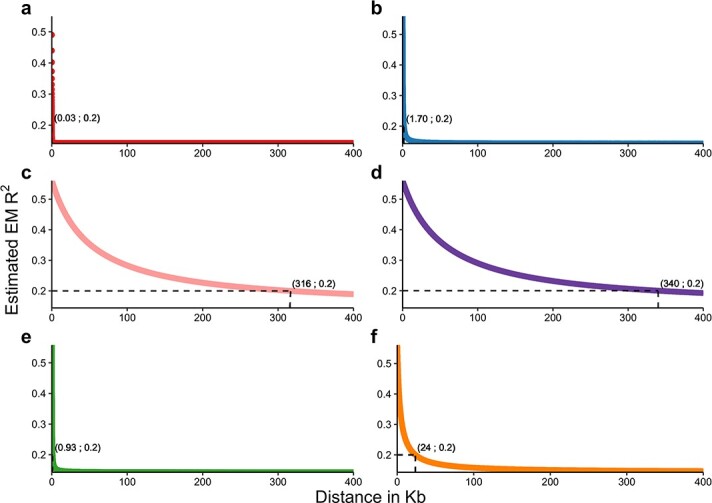
Average linkage disequilibrium (LD) decay. Curves refer to *P. sativum* accessions referable to the whole germplasm panel (a) and the populations K1 (b), K2 (c), K4 (d), K5 (e) and K6 (f) identified by the analysis of the genetic structure.

When examining *P. sativum* germplasm as a unique group, LD decayed to the squared correlation coefficient (R^2^) threshold value of 0.2 after 30 bp. However, the extent of LD decay varied greatly among *P. sativum* populations identified by STRUCTURE analysis, as it ranged from 0.93 Kb (K5) to 340 Kb (K4) (Fig. 2).

**Figure 3:**
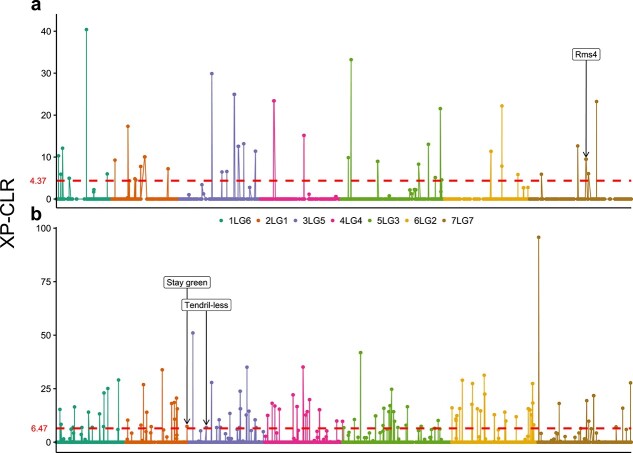
Genomic location of potential domestication (a) and breeding (b) selective sweeps, predicted by XP-CLR analysis. Dashed lines refer to XP-CLR scores corresponding to the 97^th^ percentile. The genomic positions of the *Rms4*, *Stay green*, and *Tendril-less* genes, associated with shoot branching, cotyledon colour and resistance to lodging, respectively, are also reported.

XP-CLR analysis identified 40 putative sweeps for domestication (XP-CLR scores from 4.37 to 40.40) and 98 for breeding (XP-CLR scores from 6.47 to 95.75) (Fig. 3 and Supplementary Tables S6 and S7). A genomic search was carried out to verify whether any of the pea genes reviewed by Weeden [23] as important for domestication, and by Bordat *et al*. [24] as controlling known phenotypic traits, were included in putative selective sweeps. A putative domestication sweep on chromosome 7LG7 harboured the gene *Rms4*, regulating shoot branching [23]. Two putative breeding sweeps, on chromosomes 2LG1 and 3LG5, contained the genes *Stay Green* and *Tendril-less*, which control cotyledon colour and the formation of tendrils, respectively [24] (Fig. 3 and Supplementary Tables S6 and S7).

### Validation of the SNP panel for genome-wide association studies

**Figure 4:**
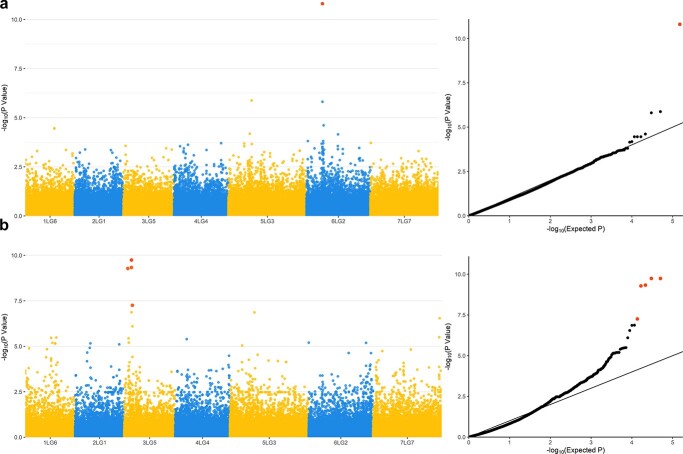
Manhattan plots and corresponding Q-Q plots resulting from GWAS for flower colour (a) and seed shape (b). Red dots indicate SNPs significantly associated with the traits after Bonferroni correction (p < 0.01).

To test the effectiveness of the SNP panel identified in our study for the identification of marker-trait associations, we performed demo genome-wide association studies using two phenotypic traits, flower colour and seed shape, controlled by the previously isolated Mendelian genes A and R [25, 26]. For flower colour, a highly significant signal was found on the pea chromosomes 6LG2, located about 64.8 Kb upstream the gene A (Fig. 4a). For seed shape, significant signals were identified on the pea chromosome 3LG5, with the leading SNP located about 13.3 Mb upstream the gene R (Fig. 4b).

**Table 2:** F_ST_ distance matrix. The composition of the populations (K) subjected to F_ST_ calculation was the same as the one resulting from the analysis of genetic structure, except for the removal of *P. fulvum* accessions from K1

	K1	K2	K3	K4	K5	K6
K1						
K2	0.42					
K3	0.42	0.43				
K4	0.48	0.45	0.44			
K5	0.38	0.30	0.31	0.34		
K6	0.44	0.36	0.38	0.39	0.12	

## Discussion

The CREA and the USDA PSPP + *P. fulvum* GBS libraries were obtained with the same restriction enzyme (*ApeK*I), thus it was expected that they were targeting largely overlapping genomic regions. However, different approaches (100 bp x single end vs. 150 bp x paired ends) and platforms (HiSeq 2500 vs. HiSeq X) were used for library sequencing, which explain the significantly different breadth of coverage observed for the two libraries (Table 1 and Supplementary Fig. S1), and consequent loss of information when filtering for SNP loci called in both germplasm collections. Nonetheless, this did not prevent the obtainment of one of the largest SNP datasets ever used to study pea genomic variation, consisting of 652 accessions and 22 127 markers. Overall, this encourages the scientific community to share and combine raw GBS data to explore the biodiversity of crops and related species in a cost-effective manner.

The identification of duplicates within and across gene banks is of major importance for the rational management of germplasm collections [12]. The low rate of putative duplicates observed for the CREA collection is indicative of low genetic redundancy, and thus emphasizes the value of this collection for studies on genetic diversity and breeding. The accessions here classified as putative duplicates (Supplementary Table S3) may indeed differ for one or a few phenotypic traits. This was verified for putative duplications found within the CREA collection, as, on the ground of data recorded by Annicchiarico *et al*. [9], only two accessions (from neighbouring countries) displayed no morphophysiological difference.

The boundary between wild *P. s. elatius* and cultivated *P. s. sativum* is blurred, due to the interfertility between these two taxa and the occurrence of early domesticated forms [3]. Indeed, accessions recorded in genebanks as *P. s*. *elatius* sometimes display phenotypes that are typical of the cultivated status, including absence of seed dormancy and pod indehiscence [13, 27]. Here, parametric analysis of genetic structure (Fig. 1a) allowed us to define an ancestral population (K1) referable to *P. fulvum* and a subset of *P. s. elatius* accessions. Therefore, this subset was deemed with confidence as truly wild. The accessions PI 639957 and PI 639959, collected in the adjacent provinces of Dyarbakir and Adiyaman in Southeastern Turkey, were the first to diverge from accessions assigned to K1 in the pea phylogeny (Fig. 1b), suggesting they might be the result of early domestication. Remarkably, this hypothesis is supported by two independent lines of evidence: (i) archaeobotanical remains indicate that the Neolithic village of Çayönü, in the province of Dyarbakir, was one of the first pea domestication sites [5]; (ii) according to USDA phenotypic records, both PI 639957 and PI 639959 have indehiscent pods. Several accessions from Bulgaria, Eastern Turkey, and Turkmenistan also displayed close phylogenetic relationship with germplasm assigned to K1, suggesting that this area was a further early set of pea cultivation. In accordance, pea seed remains have been traced back to Neolithic Bulgaria [5].

Besides K1, other five ancestral populations were detected by the STRUCTURE algorithm (Fig. 1a). The identification of ancestral populations referable to “Afghanistan” landraces (K2) and winter landraces and cultivars (K4) is consistent with the results of previous studies on pea molecular and morphophysiological diversity [4, 8, 9, 16, 28]. Two accessions from Georgia and one from Russia were assigned to population K3, indicating the possible occurrence of an ancestral population in the Caucasian region. The identification of a population mainly referable to landraces cultivated in Ethiopia, India, and Southeastern Asia (K5) might result from the introduction and exchange of germplasm in these specific areas. Finally, K6, associated with cultivars released in Europe and U.S., is likely reflecting recent common ancestry due to kinship among genotypes used in breeding programs.

The admixture model implemented in the STRUCTURE algorithm assumes an history in which populations isolated for a long time recently interbred [29]. This may not hold for pea, a self-pollinated species whose evolution has been shaped by human activities, therefore caution should be taken in interpreting admixed individuals as the product of population interbreeding [29]. In this context, accessions classified as admixed and displaying the wild K1 ancestry might be interpreted not only as feral forms resulting by hybridization with *P. s. elatius*, but also as early domesticated forms. These accessions mostly originate from a zone (here referred as Z1) including the Fertile Crescent, Southeastern Balkans, Northern Africa, and the Caspian Region (Fig. 1d), which can be therefore assumed to delimit the pea early cultivation area. Accordingly, accessions showing admixture among cultivated populations might reflect hybridization, especially in the case of modern cultivars, or evolutionary intermediates among the different ancestral populations identified by STRUCTURE analysis.

**Figure 5:**
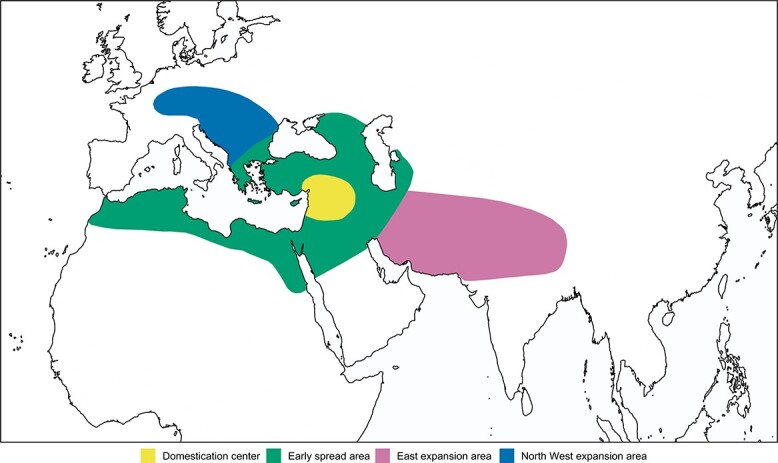
Model describing the early phases of the pea cultivation history. Starting from the Fertile Crescent, pea diffused into a macroregion also including Southeastern Balkans, Northern Africa, and the Caspian Region (Z1). In a second phase, independent expansions occurred towards east (Central Asia, Z2), and North West (the remaining part of the Balkans, Central Europe and France, Z3).

The continuous pattern of genetic differentiation from wild to cultivated germplasm highlighted by the main PC axis (PC1) provides further indication that pea cultivation was brought from the domestication centre to other regions of Z1 (Fig. 1c). In addition, it suggests a scenario (Fig. 5) in which, starting from Z1, pea was first introduced to Central Asia (Z2) and the remaining part of the Balkans, Central Europe, and France (Z3), and then to other regions of the world. Most likely, the expansion of pea cultivation from Z1 occurred independently eastwards and westwards, as accessions from Z2 and Z3 exhibited markedly different ancestries (Fig. 1c and 1d). Within Z2 and Z3, peculiar histories led to the evolution of K2 and K4, as the accessions included in these populations formed two well-defined monophyletic clades (Fig. 1b) and were associated with specific patterns of variation identified by PCA (Fig. 1c and Supplementary Fig. S5). In accordance with this evidence, accessions referable to K2 and K4 were previously shown to display distinctive phenotypes, namely resistance to nodulation by European strains of the root symbiont *Rhizobium leguminosarum* and winter frost tolerance, respectively [8, 15, 30].

K4 and K6 displayed lower genetic diversity than the other populations identified by STRUCTURE analysis. As several modern cultivars were assigned to these populations, this result is most likely to reflect genetic erosion caused by breeding. Pea domestication was predicted to cause a mild genetic bottleneck [15], however we found that the extent of genetic diversity of *P. s. elatius* accessions grouping in the population K1 was slightly lower than the one displayed by *P. s. sativum* accessions grouping in K2 and K3. We explain this by the relative low number of *P. s. elatius* accessions grouping in K1, which might not be representative of the diversity occurring in the wild.

SNPs called in this study, differently from those previously reported for the PSPP + *P. fulvum* germplasm collection [8], could be physically mapped onto the recently released pea genome [13], and therefore used for the genome-wide characterization of LD decay and selection signatures. In accordance with previous studies [31, 32], markedly different rates of LD decay were observed among intraspecific populations (Fig. 2). The slow LD decay observed for K2 and K4 might reflect strong bottlenecks and adaptation to specific environmental niches shaping the evolution of these two specific populations. Moderately slow LD decay in K6 might also be the result of anthropic selection for key traits associated with improved cultivars, and relatedness among lines used in breeding programs.

The average LD decay is a key factor in genome-wide association studies (GWAS), as it influences the chance to detect marker-trait associations on the one hand, and the chance to identify genes underlying phenotypes of interest on the other hand [14]. In this respect, our results may provide useful information for the choice of the type and the size of germplasm collections to be used for pea GWAS, when adopting GBS or other genotyping methods.

Genome scanning by the XP-CLR method led to the identification of putative selective sweeps associated with domestication and breeding (Fig. 3 and Supplementary Tables S6 and S7), thus providing a basis for future functional studies. Notably, one of the putative domestication sweeps contained the gene *Rms4*, encoding for a F-box protein known to regulate basal branching through strigolactone perception [33]. Therefore, anthropic selection of *Rms4* variants might be at the basis of the dramatic reduction of basal branching displayed by cultivated pea compared with the wild type [34]. Indeed, the involvement of a gene of the *Rms* series in pea domestication was assumed earlier [23]. As for putative breeding sweeps, two of them included the genes *Stay-green* (*Sgr*), involved in chlorophyll degradation, and *Tendril-less (Tl),* regulating tendril formation in pea leaves [35, 36]. This is consistent with the development of cultivars retaining green cotyledons at harvest and resistant to lodging through the formation of mutual tendril support. We predict that the use of denser marker data and different sets of germplasm may result in the identification of sweeps that are not reported in this study and include genes previously suggested to represent main selection targets, such as those responsible for determinate growth and day neutral flowering [23].

Most of the SNPs identified in our study (86.4%) lies within genes. This is consistent with the notion that GBS libraries prepared with the methylation-sensitive enzyme *ApeK*I tends to avoid repetitive sequences, which are extremely abundant in the pea genome [10, 13, 37, 38]. Genic SNPs have higher chance to reveal significant marker-trait associations [39, 40], therefore we envisage that our SNP dataset, allowing the correct mapping of the Mendelian genes A and R in demo studies (Fig. 4), may serve as a framework for future GWAS experiments addressing the mapping and isolation of genes of importance for pea breeding.

## Materials and methods

### DNA extraction and obtainment of GBS data

DNA from the CREA germplasm collection [9] (Supplementary Table S2) was extracted from leaf green tissues using the DNeasy Plant Mini Kit (Qiagen) and checked for integrity on 1% agarose gel. DNA quantitation was performed using the Quant-iTTM PicoGreen dsDNA assay kit (Life Technologies). A GBS library was prepared by the Elshire Group Ltd. using the *ApeK*I restriction enzyme, following the protocol described by Elshire *et al*. [10]. Library sequencing was performed using the Illumina HiSeq X platform and paired-end runs (2 x 150 bp).

DNA from the PSPP + *P. fulvum* collection (Supplementary Table S2) was previously used to prepare an *ApeK*I GBS library, which was sequenced using the Illumina HiSeq 2500 platform and single-end runs (1 x 100 bp) [8]. FASTQ files referring to this experiment were downloaded from the Short Read Archive (SRA) database (BioProject accession ID: PRJNA379298). Data referring to Abyssinian pea were removed, as this taxon was only represented by three accessions.

### Library characterization, SNP calling and quality control

FASTQ files from the CREA and PSPP + *P. fulvum* GBS libraries were separately subjected to pre-processing, using Trimmomatic [41], and alignment against the pea reference genome v1a [13], using the Burrows-Wheeler Aligner [42]. Both tools were run through the dDocent pipeline [43]. Breadth of coverage associated with the two libraries was assessed as the amount of nucleotide sites with a minimum average depth (given by the number of reads divided by the number of samples) of 1x, using the genomecov tool of the BEDTools suite [44]. BAM files were joined and sorted. Finally, SNP calling was performed using the dDocent pipeline, which internally makes use of the FreeBayes tool [45], with options -m 5 (minimum mapping quality), −q 5 (minimum base quality), —haplotype-length 3, —min-repeat-entropy 1, —binomial-obs-priors-off, —use-best-n-alleles 10.

Quality control was carried out using TASSEL 5.2.31 [46]. Specifically, biallelic SNPs were selected and filtered for minor allele frequency (MAF) > 0.01. As the PSPP + *P. fulvum* collection included 66% of the individuals subjected to SNP call, filtering for call rate > 70% was applied to ensure the selection of SNP loci called in both the PSPP + *P. fulvum* and the CREA collections. Finally, filters for SNPs with inbreeding coefficient > 0.7 and accessions with less than 30% of missing data were applied. The vcftools software suite [47] was used to calculate the transition/transversion rate and for SNP annotation. To validate the SNP quality, the Primer3 tool [48] was used to randomly design primer pairs spanning the seven pea chromosomes, which flanked one or more SNPs resulting from the SNP call and filtering procedures (Supplementary Table S1). Then, Sanger sequencing was performed on amplicons obtained from polymorphic accessions.

Accessions with pairwise identity by state (IBS) distance >0.99 were joined to form groups of putatively duplicated accessions (duplication groups or DGs). The IBS threshold of 0.95 was chosen to form groups of closely related accessions (similarity groups or SGs). Within each SG, the accession displaying the lowest amount of missing data was retained for downstream analyses.

### Genetic structure analysis

Genetic structure was investigated using SNPs in approximate linkage equilibrium (r^2^ < 0.2), using SVS v.8.9.0 (Golden Helix). The parametric model implemented in the software STRUCTURE v.2.3.4. [18] was run for a number of populations (K) ranging from 1 to 10, using 10 runs for each K, a burn in period of 25 000 and 50 000 Markov chain Monte Carlo iterations. StrAuto [49] was used to speed up the analysis. The software Structure Harvester [50], based on the ΔK statistics [19], was used to infer the number of populations best describing genetic variation in the dataset. Individuals were assigned to a specific population when their membership coefficient (qi) were higher than 0.8, otherwise they were considered admixed among different populations. Pairwise genetic differentiation (F_ST_) among populations and nucleotide diversity (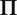) within populations were calculated using SVS v.8.9.0 and MEGA [51], respectively, after removing *P. fulvum* accessions from K1.

Hierarchical clustering was performed based on the allele-sharing distance and the neighbour joining (NJ) algorithm, using the AWclust, poppr and ape R packages [52, 53, 54]. Each node of the tree was statistically tested with 1 000 bootstrap replicates.

PCA was carried out using the SNPRelate package [55]. *P. fulvum* accessions were excluded prior to analysis to capture patterns of variation mainly reflecting the pea cultivation history*.* A scatter pie plot combining PCA coordinates and membership coefficients associated with each accession was obtained using the scatterpie R package [56].

### Characterization of LD decay and putative selective sweeps

The study of LD decay was carried out after removing *P. fulvum* from the SNP dataset, using SVS v.8.9.0. LD, expressed as squared correlation coefficient (R^2^) of the allelic state at adjacent loci, was calculated for all the accessions and for each population identified by STRUCTURE for K = 6, except for K3, which only counted three individuals. A curve describing the average LD decay against physical distance was fit using the expectation–maximization (EM) algorithm.

Putative selective sweeps were identified through the cross-population composite likelihood ratio (XP-CLR) test [57], based on multilocus allele frequency differentiation between groups. Specifically, to detect domestication sweeps, accessions grouped in K1 (except for those belonging to *P. fulvum*) were compared with cultivated germplasm (except for accessions displaying more than 1% admixture with K1). To identify improvement sweeps, accessions grouped in K6, referable to improved cultivars, were compared with other cultivated germplasm (except for accessions displaying more than 1% admixture with K6). A sliding window approach was used, based on a window size of 1 Mb, a step size of 200 Kb, and the occurrence of at least 5 SNP loci in the window. Putative selective sweeps were defined for windows with XP-CLR scores exceeding the 97^th^ percentile. Genes within putative sweeps were extracted using the intersect function of the BEDTools package [44]. The occurrence, in putative selective sweeps, of genes predicted in scientific literature to have a role in domestication [23], or controlling known phenotypic traits [24], was verified by blasting these genes against the pea genome [13].

### Phenotyping and genome-wide association study (GWAS)

Ten plants of each accession of the CREA collection were grown in 2020 at the experimental farm “P. Martucci” of the University of Bari (Bari, Italy, 41°01′22.1″N 16°54′21.0″E). The flower colour and the seed shape were scored as binary traits (white or pigmented and smooth or wrinkled, respectively). The EMMAX linear mixed model [58] was used to perform GWAS, using the pairwise identity-by-state (IBS) matrix among accessions as a covariance matrix of random effects. The Bonferroni correction was used to indicate association for P < 0.01. The genomic coordinates of the A and R genes, known to control flower colour and seed shape in pea [25, 26], were retrieved by BLAST search against the pea reference genome v1a [13].

## Acknowledgments

Genotyping of the CREA pea collection was funded through the project: “Plant Genetic Resources – FAO Treaty”, granted by the Italian Ministry of Agricultural, Food and Forestry Policies; data analysis was partly funded through the project: “LEgume GEnetic REsources as a tool for the development of innovative and sustainable food TEchnological system” supported under the “Thought for Food” Initiative by Agropolis Fondation (through the “Investissements d’avenir” program with reference number ANR-10-LABX-0001-01), Fondazione Cariplo, and Daniel & Nina Carasso Foundation.

## Author contributions

SP and PA planned and designed the study and provided funding acquisition. BF prepared the samples for GBS analysis. CD, SP, NN, NDA, FT and CL performed data analysis. SP wrote the manuscript. LR, CL, and PA critically revised the manuscript. All authors read and approved the manuscript.

## Data availability

Raw FASTQ files were deposited at the SRA database under the BioProject identification number PRJNA719084 (https://www.ncbi.nlm.nih.gov/bioproject/719084).

## Conflict of interest

The authors declare that they have no conflict of interest.

## Supplementary data

Supplementary data is available at *Horticulture Research Journal* online.

## Supplementary Material

Web_Material_uhab062
