# Supplementary material for: Merging genotyping-by-sequencing data from two ex situ collections provides insights on the pea evolutionary history
Source: Hortic Res. 2022 Jan 19;9:uhab062. doi: 10.1093/hr/uhab062 (PMC8935929; doi:10.1093/hr/uhab062)
Supplement: Web_Material_uhab062 [file web_material_uhab062.zip › Supporting Information Figures.docx]

**Supporting Information Figures**

**Article title:** Merging genotyping-by-sequencing data from two *ex situ* collections provides insights on the pea evolutionary history

**Authors:** Stefano Pavan, Chiara Delvento, Nelson Nazzicari, Barbara Ferrari, Nunzio D’Agostino, Francesca Taranto, Concetta Lotti, Luigi Ricciardi, Paolo Annicchiarico

**
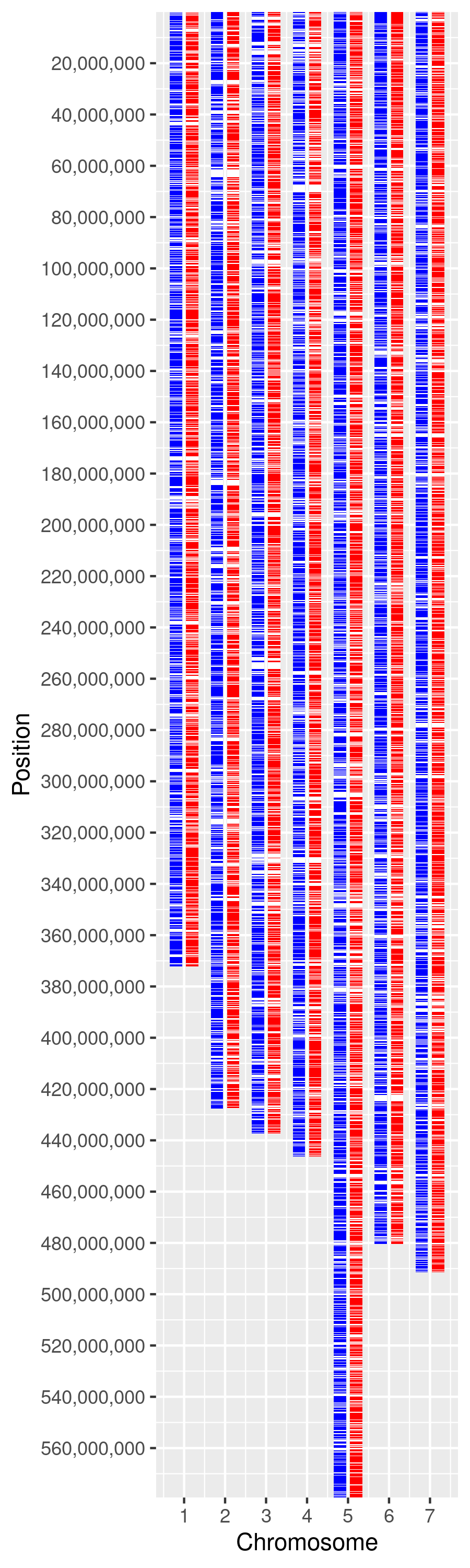
Fig. S1.** Genomic coverage associated with the CREA (blue) and the PSPP + *P. fulvum* (red) GBS libraries. Each line represents a genomic window of 10 Kb containing sites with an average depth of at least 1x.

**
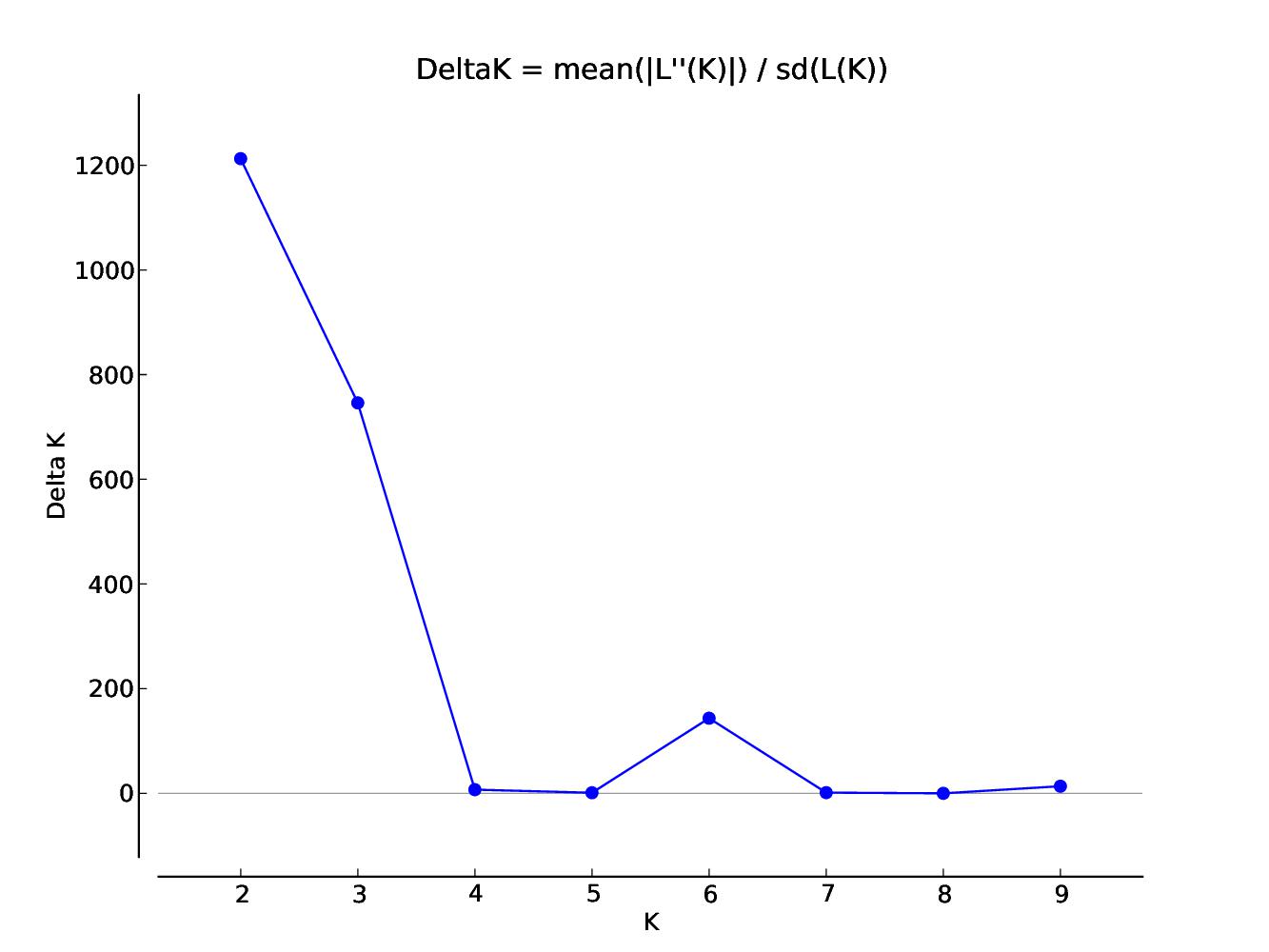
Fig. S2.** Distribution of the Evanno’s ΔK parameter.


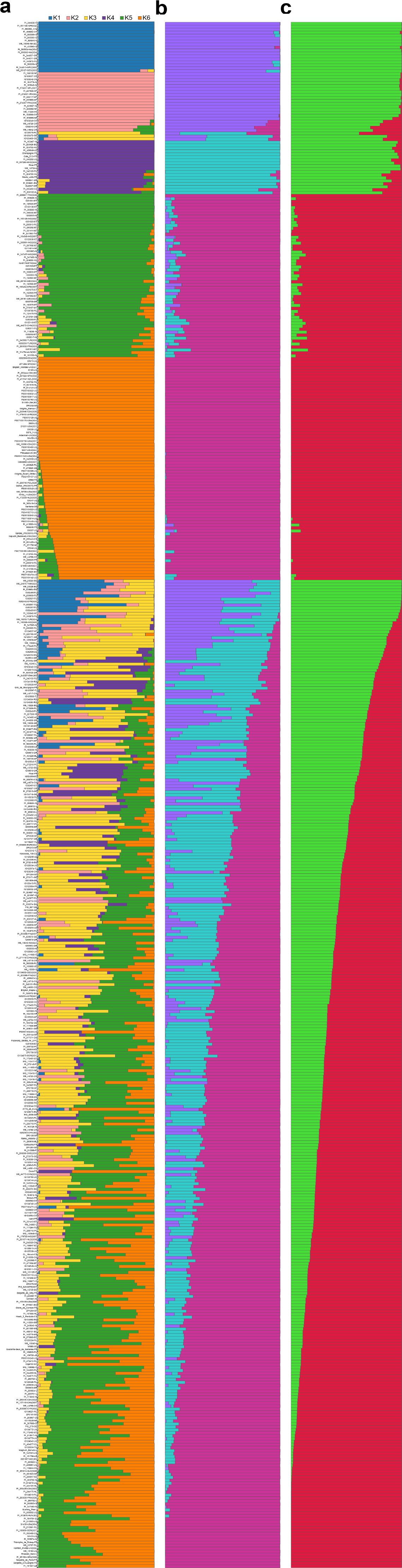
**Fig. S3.** Genetic structure identified for (a) K=6, (b) K=3 and (c) K=2.

**
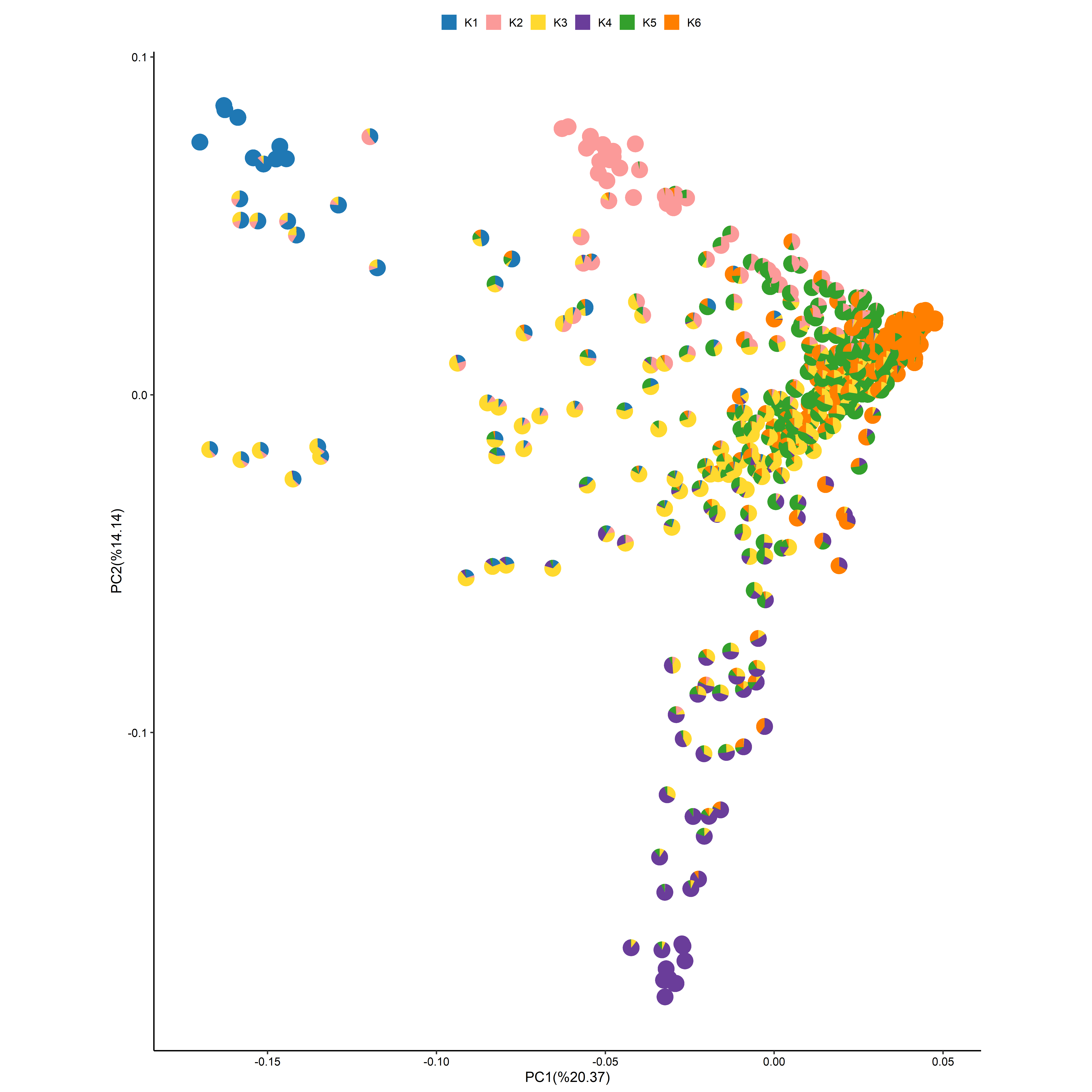
Fig. S4.** Scatter plot for *P. sativum* diversity explained by the first two principal components (PCs). Each sample is represented by a pie chart, in which colours, in accordance with Fig. 2, indicate the proportion of the genome referable to each of the six ancestral populations identified by STRUCTURE analysis.

**
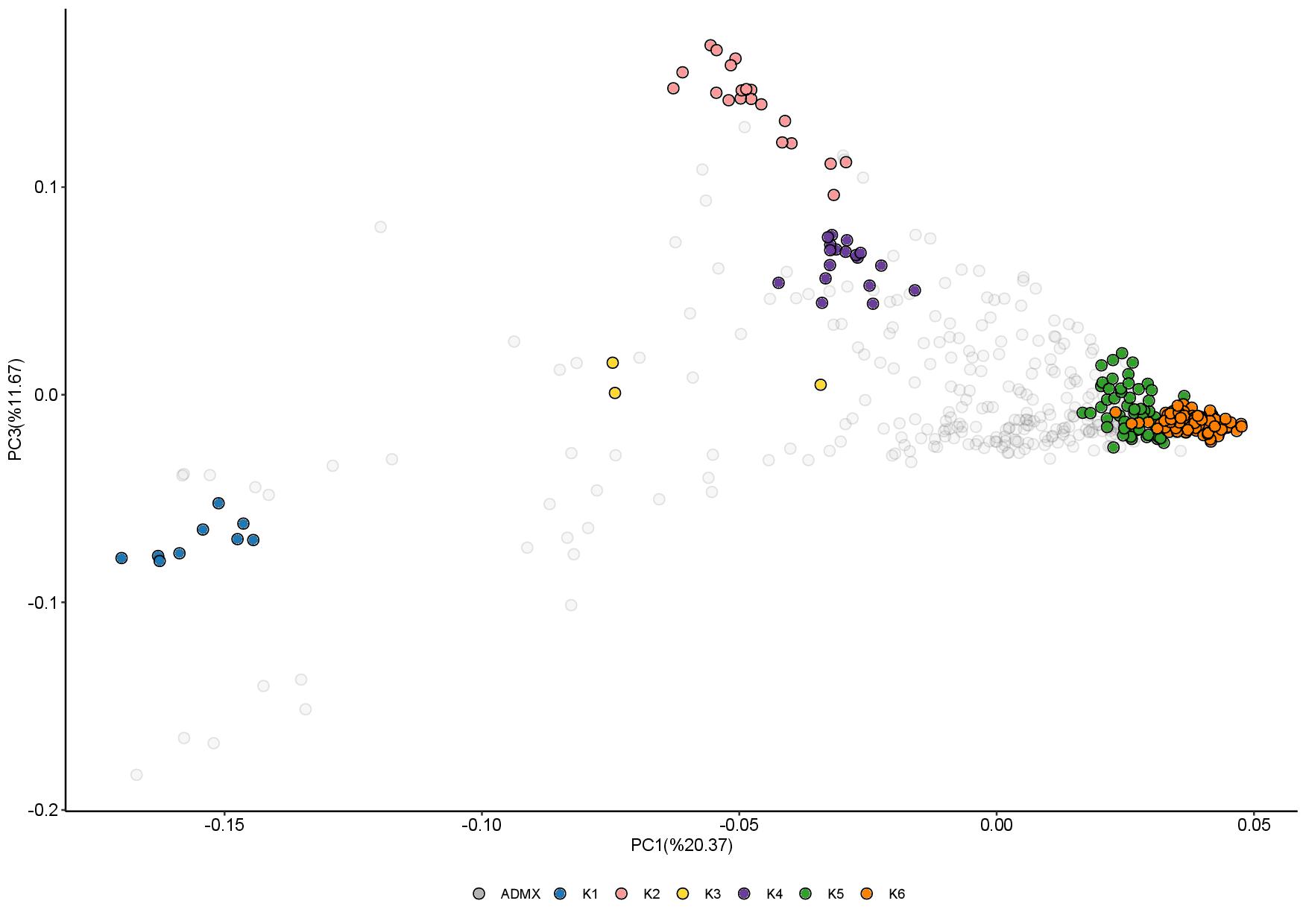
Fig. S5.** PCA plot for the first and the third principal components.
