# Supplementary material for: Merging genotyping-by-sequencing data from two ex situ collections provides insights on the pea evolutionary history
Source: Hortic Res. 2022 Jan 19;9:uhab062. doi: 10.1093/hr/uhab062 (PMC8935929; doi:10.1093/hr/uhab062)
Supplement: Web_Material_uhab062 [file Supplementary_Tables_R2.pdf › Supplementary_Tables_R2.pdf]

Supporting Information Tables

Article title: Merging genotyping-by-sequencing data from two ex situ collections provides insights on the pea evolutionary history

Authors: Stefano Pavan, Chiara Delvento, Nelson Nazzicari, Barbara Ferrari, Nunzio D'Agostino, Francesca Taranto, Concetta Lotti, Luigi Ricciardi, Paolo Annicchiarico

Table S1. Sanger sequencing validation of polymorphisms resulting from the SNP call and filtering procedures. For each PCR fragment subjected to sequencing, information is provided on the pertaining chromosome, the primer combination used, and the number of validated SNPs.

Chromosome	Primer Sequence (5' → 3')		Number of validated SNPs
	Forward	Reverse	
Chr1LG6	AGAGATAGCGCGGAG	CCTTCTGCCGCTGATGAATC	1
Chr1LG6	TCATGATCATCCTCACG	GCAGCCTGGGGATCTAACTT	2
Chr2LG1	TGTGCCAGGTGAGTGA	CACCAAGGCTTCTCAAACGT	1
Chr2LG1	TCGAATCCTACCACCCA	TGACCGATACGCTGTGCTAT	2
Chr3LG5	GCAGCCACCTCTTTGTT	TTTTGAACCGCGGTGACATT	1
Chr4LG4	CGTTTCCCTTCGTCGCT	TCGCACTGAACTCACCTCAA	1
Chr4LG4	ACGTGAATGAAGCATC	AGCAGCATTCGAGAAAACCG	1
Chr5LG3	ACACTCGCTAACAAGC	AAAACCTATGATCGCCGCAC	2
Chr6LG2	CAACGGTTGCTCCTCCA	GACCCTCCAACCTTAGTCCC	4
Chr6LG2	TCAGCTGCTTCTTGTGG	AGGAAACGGTGTAGCTACGA	1
Chr7LG7	AAGGTCCGAACGAGG[illegible]	ACACTTCTGCACCACCACTA	3
Chr7LG7	CACAGCACAGCCAGTT	ACCTCAGCAGCATCCACTAT	1

Table S2. Accessions from the PSPP + *P. fulvum* and the CREA collections considered in this study. The scientific name and the biological status of each accessions were retrieved through the Genesys database (https://www.genesys-pgr.org/), using information from USDA for the PSPP + *P. fulvum* collection and information from CREA or the donor institute for CREA collection. Accessions classified as *Pisum sativum* L. subsp. *asiaticum* and *transcaucasicum* are reported as *Pisum sativum* L. subsp. *sativum* in accordance with the common taxonomy. Thirty-five accessions displaying > 30% of missing SNP data, which were excluded from downstream analyses, are highlighted in yellow.

Accession	Origin	Holding institute	Scientific name	Biological status
Alderman	United Kingdom	CREA	*Pisum sativum* L.	Improved cultivar
Attika	France	CREA	*Pisum sativum* L.	Improved cultivar
Cartouche	France	CREA	*Pisum sativum* L.	Improved cultivar
Champagne	France	CREA	*Pisum sativum* L.	Traditional cultivar/Landrace
Chemin Long	France	CREA	*Pisum sativum* L.	Improved cultivar
Cigarron	Spain	CREA	*Pisum sativum* L.	Improved cultivar
CL 19cvs1	Czech Republic	CREA	*Pisum sativum* L.	Traditional cultivar/Landrace
Cote D'Or	France	CREA	*Pisum sativum* L.	Improved cultivar
Dove	France	CREA	*Pisum sativum* L.	Improved cultivar
Emerald Gem	United Kingdom	CREA	*Pisum sativum* L.	Breeding/Research Material
Enduro	France	CREA	*Pisum sativum* L.	Improved cultivar
English Maple	United Kingdom	CREA	*Pisum sativum* L.	Improved cultivar
English Wonder	United Kingdom	CREA	*Pisum sativum* L.	Improved cultivar
Englishsabel	United Kingdom	CREA	*Pisum sativum* L.	Improved cultivar
Fillbasket	United Kingdom	CREA	*Pisum sativum* L.	Improved cultivar
Genial (PSC0073)	France	CREA	*Pisum sativum* L.	Improved cultivar
Gloire de Correze	France	CREA	*Pisum sativum* L.	Improved cultivar
Gris de Bourgogne	France	CREA	*Pisum sativum* L.	Traditional cultivar/Landrace
Haute Loire	France	CREA	*Pisum sativum* L.	NA
Hrach Z Pardubic	Czech Republic	CREA	*Pisum sativum* L.	Traditional cultivar/Landrace
IG112140	Morocco	CREA	*Pisum sativum* L.	Wild
IG114899	Nepal	CREA	*Pisum sativum* L.	Traditional cultivar/Landrace
IG114914	Nepal	CREA	*Pisum sativum* L.	Traditional cultivar/Landrace
IG114977	Nepal	CREA	*Pisum sativum* L. subsp. *sativum*	Traditional cultivar/Landrace
IG115100	Nepal	CREA	*Pisum sativum* L.	Traditional cultivar/Landrace
IG115114	Nepal	CREA	*Pisum sativum* L.	Traditional cultivar/Landrace
IG49181	Afghanistan	CREA	*Pisum sativum* L.	Traditional cultivar/Landrace
IG115228	Nepal	CREA	*Pisum sativum* L.	Traditional cultivar/Landrace
IG115266	Nepal	CREA	*Pisum sativum* L.	Traditional cultivar/Landrace
IG115331	Nepal	CREA	*Pisum sativum* L. subsp. *sativum*	Traditional cultivar/Landrace
IG115341	Nepal	CREA	*Pisum sativum* L.	Traditional cultivar/Landrace
IG116232	Turkey	CREA	*Pisum sativum* L.	Wild
IG116297	Turkey	CREA	*Pisum sativum* L.	Traditional cultivar/Landrace
IG116455	Turkey	CREA	*Pisum sativum* L.	Traditional cultivar/Landrace
IG122966	Turkmenistan	CREA	*Pisum sativum* L. subsp. *sativum*	Traditional cultivar/Landrace
IG122974	Tajikistan	CREA	*Pisum sativum* L. subsp. *sativum*	Traditional cultivar/Landrace
IG122996	Kazakhstan	CREA	*Pisum sativum* L.	Traditional cultivar/Landrace
IG123004	Tunisia	CREA	*Pisum sativum* L. subsp. *sativum*	NA
IG123006	Ethiopia	CREA	*Pisum sativum* L. subsp. *sativum*	Traditional cultivar/Landrace
IG123021	Afghanistan	CREA	*Pisum sativum* L. subsp. *sativum*	Traditional cultivar/Landrace
IG123028	Afghanistan	CREA	*Pisum sativum* L. subsp. *sativum*	Traditional cultivar/Landrace
IG123029	Afghanistan	CREA	*Pisum sativum* L. subsp. *sativum*	Traditional cultivar/Landrace
IG123034	Afghanistan	CREA	*Pisum sativum* L. subsp. *sativum*	Traditional cultivar/Landrace
IG123041	Kazakhstan	CREA	*Pisum sativum* L. subsp. *sativum*	Traditional cultivar/Landrace
IG123050	Turkey	CREA	*Pisum sativum* L. subsp. *sativum*	Traditional cultivar/Landrace
IG123073	Algeria	CREA	*Pisum sativum* L. subsp. *sativum*	Traditional cultivar/Landrace
IG123080	Palestine	CREA	*Pisum sativum* L.	Traditional cultivar/Landrace
IG123084	Palestine	CREA	*Pisum sativum* L. subsp. *elatius*	NA
IG123085	Lebanon	CREA	*Pisum sativum* L. subsp. *elatius*	NA
IG52367	Afghanistan	CREA	*Pisum sativum* L.	Traditional cultivar/Landrace
IG123118	Ethiopia	CREA	*Pisum sativum* L. subsp. *sativum*	Traditional cultivar/Landrace
IG123136	Ethiopia	CREA	*Pisum sativum* L. subsp. *sativum*	Traditional cultivar/Landrace
IG123211	China	CREA	*Pisum sativum* L. subsp. *sativum*	NA
IG123227	China	CREA	*Pisum sativum* L. subsp. *sativum*	NA
IG123237	China	CREA	*Pisum sativum* L. subsp. *sativum*	NA
IG123240	China	CREA	*Pisum sativum* L. subsp. *sativum*	NA
IG123242	China	CREA	*Pisum sativum* L. subsp. *sativum*	NA
IG123244	China	CREA	*Pisum sativum* L. subsp. *sativum*	NA
IG123245	China	CREA	*Pisum sativum* L. subsp. *sativum*	NA
IG123248	China	CREA	*Pisum sativum* L. subsp. *sativum*	NA
IG123280	Uzbekistan	CREA	*Pisum sativum* L. subsp. *sativum*	NA
IG123281	Uzbekistan	CREA	*Pisum sativum* L. subsp. *sativum*	NA
IG123288	Uzbekistan	CREA	*Pisum sativum* L. subsp. *sativum*	NA
IG123311	Kazakhstan	CREA	*Pisum sativum* L. subsp. *sativum*	NA
IG123312	Tajikistan	CREA	*Pisum sativum* L. subsp. *sativum*	Traditional cultivar/Landrace
IG123313	Morocco	CREA	*Pisum sativum* L. subsp. *sativum*	NA
IG124664	China	CREA	*Pisum sativum* L.	NA
IG124843	Ukraine	CREA	*Pisum sativum* L.	Traditional cultivar/Landrace
IG124857	Afghanistan	CREA	*Pisum sativum* L.	Traditional cultivar/Landrace
IG125324	Russia	CREA	*Pisum sativum* L. subsp. *sativum*	NA
IG125326	Russia	CREA	*Pisum sativum* L. subsp. *sativum*	NA
IG125336	Ukraine	CREA	*Pisum sativum* L. subsp. *sativum*	NA
IG125378	Russia	CREA	*Pisum sativum* L. subsp. *sativum*	NA
IG125415	Russia	CREA	*Pisum sativum* L. subsp. *sativum*	NA
IG125469	Georgia	CREA	*Pisum sativum* L. subsp. *sativum*	Traditional cultivar/Landrace
IG125471	Georgia	CREA	*Pisum sativum* L. subsp. *sativum*	Traditional cultivar/Landrace
IG125472	Georgia	CREA	*Pisum sativum* L. subsp. *sativum*	Traditional cultivar/Landrace
IG125543	Tajikistan	CREA	*Pisum sativum* L. subsp. *sativum*	NA
IG125550	China	CREA	*Pisum sativum* L. subsp. *sativum*	NA
IG125589	Kyrgyzstan	CREA	*Pisum sativum* L. subsp. *sativum*	Traditional cultivar/Landrace
IG125597	Tajikistan	CREA	*Pisum sativum* L. subsp. *sativum*	Traditional cultivar/Landrace
IG125600	Tajikistan	CREA	*Pisum sativum* L. subsp. *sativum*	NA
IG128856	Georgia	CREA	*Pisum sativum* L. subsp. *sativum*	NA
IG128863	Ukraine	CREA	*Pisum sativum* L. subsp. *sativum*	Traditional cultivar/Landrace
IG128887	Russia	CREA	*Pisum sativum* L. subsp. *sativum*	Traditional cultivar/Landrace
IG128913	Ukraine	CREA	*Pisum sativum* L. subsp. *sativum*	Traditional cultivar/Landrace
IG128934	Ukraine	CREA	*Pisum sativum* L. subsp. *sativum*	NA
IG128973	Bulgaria	CREA	*Pisum sativum* L. subsp. *sativum*	NA
IG128983	Russia	CREA	*Pisum sativum* L. subsp. *sativum*	Traditional cultivar/Landrace
IG129002	Greece	CREA	*Pisum sativum* L. subsp. *sativum*	NA
IG134060	Romania	CREA	*Pisum sativum* L.	NA
IG134080	Romania	CREA	*Pisum sativum* L.	NA
IG134094	Romania	CREA	*Pisum sativum* L.	NA
IG134109	Romania	CREA	*Pisum sativum* L.	NA
IG134609	Georgia	CREA	*Pisum sativum* L.	NA
IG134619	Russia	CREA	*Pisum sativum* L.	NA
IG134621	Russia	CREA	*Pisum sativum* L.	NA
IG134649	Ukraine	CREA	*Pisum sativum* L.	NA
IG134707	Georgia	CREA	*Pisum sativum* L.	NA
IG134718	Georgia	CREA	*Pisum sativum* L.	NA
IG134744	Ukraine	CREA	*Pisum sativum* L.	NA
IG134746	Ukraine	CREA	*Pisum sativum* L.	NA
IG134750	Ukraine	CREA	*Pisum sativum* L.	NA
IG134770	Ukraine	CREA	*Pisum sativum* L.	NA
IG134772	Ukraine	CREA	*Pisum sativum* L.	NA
IG134782	Russia	CREA	*Pisum sativum* L.	NA
IG134788	Russia	CREA	*Pisum sativum* L.	NA
IG134823	Russia	CREA	*Pisum sativum* L.	NA
IG134828	Georgia	CREA	*Pisum sativum* L.	NA
IG134841	Bulgaria	CREA	*Pisum sativum* L.	NA
IG134857	Bulgaria	CREA	*Pisum sativum* L.	Breeding/Research Material
IG134862	Bulgaria	CREA	*Pisum sativum* L.	NA
IG134870	Bulgaria	CREA	*Pisum sativum* L.	NA
IG49176	Ethiopia	CREA	*Pisum sativum* L.	Traditional cultivar/Landrace
W6 44566	China	USDA	*Pisum sativum* L.	Traditional cultivar/Landrace
IG49189	Afghanistan	CREA	*Pisum sativum* L.	Traditional cultivar/Landrace
IG49203	Afghanistan	CREA	*Pisum sativum* L.	NA
W6 44573	China	USDA	*Pisum sativum* L.	Traditional cultivar/Landrace
IG49327	Greece	CREA	*Pisum sativum* L.	NA
IG49544	Georgia	CREA	*Pisum sativum* L.	NA
IG49610	Greece	CREA	*Pisum sativum* L.	NA
IG49633	Greece	CREA	*Pisum sativum* L.	NA
IG50235	India	CREA	*Pisum sativum* L.	Traditional cultivar/Landrace
IG50248	Turkey	CREA	*Pisum sativum* L.	NA
IG50250	Turkey	CREA	*Pisum sativum* L.	Traditional cultivar/Landrace
IG50303	India	CREA	*Pisum sativum* L.	Traditional cultivar/Landrace
IG50311	Turkey	CREA	*Pisum sativum* L.	Traditional cultivar/Landrace
IG50357	Turkey	CREA	*Pisum sativum* L.	NA
IG50358	Turkey	CREA	*Pisum sativum* L.	Traditional cultivar/Landrace
W6 44579	China	USDA	*Pisum sativum* L.	Traditional cultivar/Landrace
IG50559	Greece	CREA	*Pisum sativum* L. subsp. *sativum*	NA
IG50570	Greece	CREA	*Pisum sativum* L. subsp. *sativum*	NA
IG50584	Greece	CREA	*Pisum sativum* L. subsp. *sativum*	NA
IG50592	China	CREA	*Pisum sativum* L. subsp. *sativum*	NA
IG50641	Greece	CREA	*Pisum sativum* L. subsp. *sativum*	NA
IG50669	China	CREA	*Pisum sativum* L. subsp. *sativum*	NA
IG50673	Greece	CREA	*Pisum sativum* L. subsp. *sativum*	NA
IG50756	Greece	CREA	*Pisum sativum* L. subsp. *sativum*	NA
IG50935	Greece	CREA	*Pisum sativum* L. subsp. *sativum*	NA
IG51513	Ethiopia	CREA	*Pisum sativum* L. subsp. *sativum*	Traditional cultivar/Landrace
IG51516	Ethiopia	CREA	*Pisum sativum* L. subsp. *sativum*	Traditional cultivar/Landrace
IG51520	Ethiopia	CREA	*Pisum sativum* L. subsp. *sativum*	Traditional cultivar/Landrace
IG51529	Ethiopia	CREA	*Pisum sativum* L. subsp. *sativum*	Traditional cultivar/Landrace
IG51536	Ethiopia	CREA	*Pisum sativum* L. subsp. *sativum*	Traditional cultivar/Landrace
IG51551	Ethiopia	CREA	*Pisum sativum* L. subsp. *sativum*	Traditional cultivar/Landrace
IG51562	Ethiopia	CREA	*Pisum sativum* L. subsp. *sativum*	Traditional cultivar/Landrace
IG51576	Ethiopia	CREA	*Pisum sativum* L. subsp. *sativum*	Traditional cultivar/Landrace
IG51669	Greece	CREA	*Pisum sativum* L.	Traditional cultivar/Landrace
W6 44580	China	USDA	*Pisum sativum* L.	Traditional cultivar/Landrace
W6 44582	China	USDA	*Pisum sativum* L.	Traditional cultivar/Landrace
IG51891	India	CREA	*Pisum sativum* L.	NA
IG51927	India	CREA	*Pisum sativum* L.	NA
IG51948	India	CREA	*Pisum sativum* L.	NA
IG51957	India	CREA	*Pisum sativum* L.	NA
W6 44711	China	USDA	*Pisum sativum* L.	Traditional cultivar/Landrace
W6 44719	China	USDA	*Pisum sativum* L.	Traditional cultivar/Landrace
IG51991	India	CREA	*Pisum sativum* L.	NA
IG51993	India	CREA	*Pisum sativum* L.	NA
IG51994	India	CREA	*Pisum sativum* L.	NA
IG52005	India	CREA	*Pisum sativum* L.	NA
IG52017	India	CREA	*Pisum sativum* L.	NA
IG52036	China	CREA	*Pisum sativum* L.	NA
IG52040	China	CREA	*Pisum sativum* L.	NA
IG52050	India	CREA	*Pisum sativum* L.	Traditional cultivar/Landrace
IG52081	Ethiopia	CREA	*Pisum sativum* L.	Traditional cultivar/Landrace
IG52092	Ethiopia	CREA	*Pisum sativum* L.	Traditional cultivar/Landrace
W6 44721	China	USDA	*Pisum sativum* L.	Traditional cultivar/Landrace
IG52383	China	CREA	*Pisum sativum* L.	NA
IG52401	Syria	CREA	*Pisum sativum* L.	Wild
IG52417	Syria	CREA	*Pisum sativum* L.	Wild
IG52426	Syria	CREA	*Pisum sativum* L.	Wild
IG52442	Syria	CREA	*Pisum sativum* L.	Wild
IG52455	Morocco	CREA	*Pisum sativum* L.	Traditional cultivar/Landrace
IG52456	Morocco	CREA	*Pisum sativum* L.	Traditional cultivar/Landrace
W6 44722	China	USDA	*Pisum sativum* L.	Traditional cultivar/Landrace
IG52496	Turkey	CREA	*Pisum sativum* L.	Wild
IG52521	Turkey	CREA	*Pisum sativum* L.	Wild
IG52534	Syria	CREA	*Pisum sativum* L.	Wild
IG52535	Syria	CREA	*Pisum sativum* L.	Wild
IG52586	Algeria	CREA	*Pisum sativum* L. subsp. *sativum*	Traditional cultivar/Landrace
IG52595	Algeria	CREA	*Pisum sativum* L. subsp. *sativum*	Wild
IG52596	Algeria	CREA	*Pisum sativum* L. subsp. *sativum*	Wild
Isard	France	CREA	*Pisum sativum* L.	Improved cultivar
Kapucin Belokvety	Czech Republic	CREA	*Pisum sativum* L.	Traditional cultivar/Landrace
Kentish Invicta	United Kingdom	CREA	*Pisum sativum* L.	Improved cultivar
Knights Dwarf White	United Kingdom	CREA	*Pisum sativum* L.	Improved cultivar
Knights Marrow	United Kingdom	CREA	*Pisum sativum* L.	Improved cultivar
Kocovska 108	Czech Republic	CREA	*Pisum sativum* L.	Traditional cultivar/Landrace
Landrace Orava	Czech Republic	CREA	*Pisum sativum* L.	Traditional cultivar/Landrace
Magnum Bonum	United Kingdom	CREA	*Pisum sativum* L.	Improved cultivar
Messire	France	CREA	*Pisum sativum* L.	Improved cultivar
MG 100948	Italy	CREA	*Pisum sativum* L.	NA
MG 101126	Italy	CREA	*Pisum sativum* L.	NA
MG 106069	Italy	CREA	*Pisum sativum* L.	NA
MG 106871	Italy	CREA	*Pisum sativum* L.	NA
MG 110243	Italy	CREA	*Pisum sativum* L.	NA
MG 110416	Italy	CREA	*Pisum sativum* L.	NA
MG 110417	Italy	CREA	*Pisum sativum* L.	NA
MG 110418	Italy	CREA	*Pisum sativum* L.	NA
MG 111850	Italy	CREA	*Pisum sativum* L.	NA
MG 111988	Italy	CREA	*Pisum sativum* L.	NA
MG 115084	Italy	CREA	*Pisum sativum* L.	NA
Mummy Pea	United Kingdom	CREA	*Pisum sativum* L.	Improved cultivar
Picar	France	CREA	*Pisum sativum* L.	Traditional cultivar/Landrace
PIS 278	Germany	CREA	*Pisum sativum* L. subsp. *sativum*	Improved cultivar
PIS 2856	Germany	CREA	*Pisum sativum* L. subsp. *sativum*	Traditional cultivar/Landrace
PIS 657	Germany	CREA	*Pisum sativum* L. subsp. *sativum*	Traditional cultivar/Landrace
PIS 845	Germany	CREA	*Pisum sativum* L. subsp. *sativum*	Improved cultivar
Piver	France	CREA	*Pisum sativum* L.	Traditional cultivar/Landrace
Pulawska Slodka Nr 2	Poland	CREA	*Pisum sativum* L.	Traditional cultivar/Landrace
Quarante	France	CREA	*Pisum sativum* L.	NA
Raina Victoria	United Kingdom	CREA	*Pisum sativum* L.	Improved cultivar
Santana	Germany	CREA	*Pisum sativum* L.	Improved cultivar
Serpette D'Auvergne	France	CREA	*Pisum sativum* L.	Traditional cultivar/Landrace
Serpette de Paris	France	CREA	*Pisum sativum* L.	NA
Serpette de Vitry	France	CREA	*Pisum sativum* L.	NA
Spirale (PSC0013)	France	CREA	*Pisum sativum* L.	Improved cultivar
Triomphe de Roissy	France	CREA	*Pisum sativum* L.	NA
Viriato	Spain	CREA	*Pisum sativum* L.	Improved cultivar
Witham Wonder	United Kingdom	CREA	*Pisum sativum* L.	Improved cultivar
W6 44725	China	USDA	*Pisum sativum* L.	Traditional cultivar/Landrace
ZP0076	Spain	CREA	*Pisum sativum* L.	Traditional cultivar/Landrace
ZP0126	Spain	CREA	*Pisum sativum* L.	Traditional cultivar/Landrace
ZP0181	Spain	CREA	*Pisum sativum* L.	Traditional cultivar/Landrace
ZP0202	Spain	CREA	*Pisum sativum* L.	Traditional cultivar/Landrace
ZP0213	Spain	CREA	*Pisum sativum* L.	Traditional cultivar/Landrace
ZP0535	Spain	CREA	*Pisum sativum* L.	Traditional cultivar/Landrace
ZP0798	Spain	CREA	*Pisum sativum* L.	Traditional cultivar/Landrace
ZP0799	Spain	CREA	*Pisum sativum* L.	Traditional cultivar/Landrace
ZP1261	Spain	CREA	*Pisum sativum* L.	Traditional cultivar/Landrace
ZP1264	Spain	CREA	*Pisum sativum* L.	Traditional cultivar/Landrace
ZP1282	Spain	CREA	*Pisum sativum* L.	Traditional cultivar/Landrace
ZP1294	Spain	CREA	*Pisum sativum* L.	Traditional cultivar/Landrace
ZP1300	Spain	CREA	*Pisum sativum* L.	Traditional cultivar/Landrace
A778 26 6	USA	USDA	*Pisum sativum* L.	NA
Carneval	Sweden	USDA	*Pisum sativum* L.	Improved cultivar
Cascadia	USA	USDA	*Pisum sativum* L.	NA
W6 44767	China	USDA	*Pisum sativum* L.	Traditional cultivar/Landrace
W6 44773	China	USDA	*Pisum sativum* L.	Traditional cultivar/Landrace
Kiflica	Serbia	USDA	*Pisum sativum* L.	NA
M193	USA	USDA	*Pisum sativum* L.	NA
M194 1	USA	USDA	*Pisum sativum* L.	NA
OR Giant	USA	USDA	*Pisum sativum* L.	NA
OSPII	USA	USDA	*Pisum sativum* L.	NA
W6 44774	China	USDA	*Pisum sativum* L.	Traditional cultivar/Landrace
PI 102888	China	USDA	*Pisum sativum* L.	NA
PI 103058	China	USDA	*Pisum sativum* L.	NA
PI 109866	Venezuela	USDA	*Pisum sativum* L.	Improved cultivar
PI 116056	India	USDA	*Pisum sativum* L. subsp. *sativum*	NA
PI 116844	Pakistan	USDA	*Pisum sativum* L.	NA
PI 116944	Afghanistan	USDA	*Pisum sativum* L.	NA
PI 117264	Turkey	USDA	*Pisum sativum* L.	NA
PI 117998	Brazil	USDA	*Pisum sativum* L.	Improved cultivar
PI 118501	Brazil	USDA	*Pisum sativum* L.	Improved cultivar
PI 121352	India	USDA	*Pisum sativum* L.	NA
PI 124478	Pakistan	USDA	*Pisum sativum* L.	NA
PI 125839	Afghanistan	USDA	*Pisum sativum* L.	Improved cultivar
PI 125840	Afghanistan	USDA	*Pisum sativum* L.	NA
PI 134271	Afghanistan	USDA	*Pisum sativum* L.	NA
PI 137118	Canada	USDA	*Pisum sativum* L.	NA
PI 137119	Canada	USDA	*Pisum sativum* L.	Improved cultivar
PI 142775	Mexico	USDA	*Pisum sativum* L.	Improved cultivar
PI 143485	Azerbaijan	USDA	*Pisum sativum* L.	NA
PI 155109	USA	USDA	*Pisum sativum* L.	NA
PI 156647	Ethiopia	USDA	*Pisum sativum* L.	NA
PI 156720	Japan	USDA	*Pisum sativum* L.	Improved cultivar
PI 162909	Paraguay	USDA	*Pisum sativum* L.	NA
PI 163126	India	USDA	*Pisum sativum* L.	NA
PI 163129	India	USDA	*Pisum sativum* L.	NA
PI 164548	India	USDA	*Pisum sativum* L.	NA
PI 164612	India	USDA	*Pisum sativum* L.	NA
PI 164779	India	USDA	*Pisum sativum* L.	NA
PI 164971	Turkey	USDA	*Pisum sativum* L.	NA
PI 164972	Turkey	USDA	*Pisum sativum* L.	NA
PI 165949	India	USDA	*Pisum sativum* L.	NA
PI 166084	India	USDA	*Pisum sativum* L.	NA
PI 166159	Nepal	USDA	*Pisum sativum* L.	NA
PI 169608	Turkey	USDA	*Pisum sativum* L.	NA
PI 172339	Netherlands	USDA	*Pisum sativum* L.	Improved cultivar
PI 173840	India	USDA	*Pisum sativum* L.	NA
PI 174921	Nepal	USDA	*Pisum sativum* L.	NA
PI 175231	Nepal	USDA	*Pisum sativum* L.	NA
PI 179449	Turkey	USDA	*Pisum sativum* L.	NA
PI 179450	Syria	USDA	*Pisum sativum* L.	NA
PI 179451	Syria	USDA	*Pisum sativum* L.	NA
PI 179459	Turkey	USDA	*Pisum sativum* L.	NA
PI 179722	India	USDA	*Pisum sativum* L.	NA
PI 179970	India	USDA	*Pisum sativum* L.	NA
PI 180329	India	USDA	*Pisum sativum* L.	NA
PI 180693	Germany	USDA	*Pisum sativum* L.	Improved cultivar
PI 180696	Germany	USDA	*Pisum sativum* L.	Improved cultivar
PI 180699	Germany	USDA	*Pisum sativum* L.	Improved cultivar
PI 180702	Germany	USDA	*Pisum sativum* L.	Improved cultivar
PI 181799	Lebanon	USDA	*Pisum sativum* L.	NA
PI 181801	Lebanon	USDA	*Pisum sativum* L.	NA
PI 181958	Syria	USDA	*Pisum sativum* L.	NA
PI 183467	India	USDA	*Pisum sativum* L.	NA
PI 184130	Croatia	USDA	*Pisum sativum* L.	NA
PI 184784	Guinea	USDA	*Pisum sativum* L.	NA
PI 193578	Ethiopia	USDA	*Pisum sativum* L.	NA
PI 193584	Ethiopia	USDA	*Pisum sativum* L.	NA
PI 193590	Ethiopia	USDA	*Pisum sativum* L.	NA
PI 195020	Ethiopia	USDA	*Pisum sativum* L.	NA
PI 195404	Guatemala	USDA	*Pisum sativum* L.	NA
PI 195631	Ethiopia	USDA	*Pisum sativum* L.	NA
PI 197044	Honduras	USDA	*Pisum sativum* L.	NA
PI 197990	Netherlands	USDA	*Pisum sativum* L.	Improved cultivar
PI 198072	Sweden	USDA	*Pisum sativum* L.	NA
PI 198074	Sweden	USDA	*Pisum sativum* L.	NA
PI 198735	Afghanistan	USDA	*Pisum sativum* L.	NA
PI 201390	Mexico	USDA	*Pisum sativum* L.	NA
PI 203066	Finland	USDA	*Pisum sativum* L.	NA
PI 203067	Finland	USDA	*Pisum sativum* L.	NA
PI 203068	Finland	USDA	*Pisum sativum* L.	NA
PI 203069	Finland	USDA	*Pisum sativum* L.	NA
PI 204306	Australia	USDA	*Pisum sativum* L.	NA
PI 206006	Sweden	USDA	*Pisum sativum* L.	NA
PI 206838	USA	USDA	*Pisum sativum* L.	Improved cultivar
PI 206861	USA	USDA	*Pisum sativum* L.	Improved cultivar
PI 207508	Afghanistan	USDA	*Pisum sativum* L.	NA
PI 209507	Costa Rica	USDA	*Pisum sativum* L.	NA
PI 210558	China	USDA	*Pisum sativum* L.	NA
PI 210561	Russia	USDA	*Pisum sativum* L.	NA
PI 210568	Finland	USDA	*Pisum sativum* L.	Improved cultivar
PI 210569	Finland	USDA	*Pisum sativum* L.	Improved cultivar
PI 210571	Finland	USDA	*Pisum sativum* L.	Improved cultivar
PI 210583	USA	USDA	*Pisum sativum* L.	Improved cultivar
PI 212031	Iran	USDA	*Pisum sativum* L.	NA
PI 212917	India	USDA	*Pisum sativum* L.	NA
PI 220174	Afghanistan	USDA	*Pisum sativum* L.	NA
PI 220189	Afghanistan	USDA	*Pisum sativum* L.	NA
PI 221697	Indonesia	USDA	*Pisum sativum* L.	NA
PI 222071	Afghanistan	USDA	*Pisum sativum* L.	Wild
PI 222117	Afghanistan	USDA	*Pisum sativum* L.	NA
PI 227258	Iran	USDA	*Pisum sativum* L.	NA
PI 236492	USA	USDA	*Pisum sativum* L.	Improved cultivar
PI 241593	Taiwan, China	USDA	*Pisum sativum* L.	NA
PI 242027	Denmark	USDA	*Pisum sativum* L. subsp. *sativum*	NA
PI 242028	Denmark	USDA	*Pisum sativum* L.	NA
PI 244093	Netherlands	USDA	*Pisum sativum* L.	Improved cultivar
PI 244175	Netherlands	USDA	*Pisum sativum* L.	Improved cultivar
PI 244191	Netherlands	USDA	*Pisum sativum* L.	Improved cultivar
PI 248181	Rwanda	USDA	*Pisum sativum* L.	NA
PI 249645	India	USDA	*Pisum sativum* L.	NA
PI 250438	Czech Republic	USDA	*Pisum sativum* L.	Improved cultivar
PI 250439	Czech Republic	USDA	*Pisum sativum* L.	Improved cultivar
PI 250440	Czech Republic	USDA	*Pisum sativum* L.	Improved cultivar
PI 250441	Czech Republic	USDA	*Pisum sativum* L.	Improved cultivar
PI 250444	Czech Republic	USDA	*Pisum sativum* L.	Improved cultivar
PI 250446	Czech Republic	USDA	*Pisum sativum* L.	Improved cultivar
PI 250447	Czech Republic	USDA	*Pisum sativum* L.	Improved cultivar
PI 250448	Czech Republic	USDA	*Pisum sativum* L.	Improved cultivar
PI 253968	Afghanistan	USDA	*Pisum sativum* L.	NA
PI 257244	China	USDA	*Pisum sativum* L.	Improved cultivar
PI 257592	Ethiopia	USDA	*Pisum sativum* L.	NA
PI 261622	Spain	USDA	*Pisum sativum* L.	NA
PI 261623	Spain	USDA	*Pisum sativum* L.	NA
PI 261624	Spain	USDA	*Pisum sativum* L.	NA
PI 261636	Spain	USDA	*Pisum sativum* L.	NA
PI 261671	Netherlands	USDA	*Pisum sativum* L.	Improved cultivar
PI 261677	Netherlands	USDA	*Pisum sativum* L.	NA
PI 263014	Netherlands	USDA	*Pisum sativum* L.	Improved cultivar
PI 263030	France	USDA	*Pisum sativum* L.	Improved cultivar
PI 263032	France	USDA	*Pisum sativum* L.	Improved cultivar
PI 263871	Greece	USDA	*Pisum sativum* L.	NA
PI 266070	Sweden	USDA	*Pisum sativum* L.	NA
PI 269761	Czech Republic	USDA	*Pisum sativum* L. subsp. *sativum*	NA
PI 269762	United Kingdom	USDA	*Pisum sativum* L. subsp. *sativum*	NA
PI 269777	United Kingdom	USDA	*Pisum sativum* L.	NA
PI 269778	United Kingdom	USDA	*Pisum sativum* L.	NA
PI 269782	United Kingdom	USDA	*Pisum sativum* L.	Wild
PI 269791	United Kingdom	USDA	*Pisum sativum* L.	Improved cultivar
PI 269798	United Kingdom	USDA	*Pisum sativum* L.	NA
PI 269802	United Kingdom	USDA	*Pisum sativum* L.	NA
PI 269804	United Kingdom	USDA	*Pisum sativum* L.	NA
PI 269812	United Kingdom	USDA	*Pisum sativum* L.	NA
PI 269818	United Kingdom	USDA	*Pisum sativum* L.	NA
PI 269822	United Kingdom	USDA	*Pisum sativum* L.	Improved cultivar
PI 269825	United Kingdom	USDA	*Pisum sativum* L.	NA
PI 270536	Denmark	USDA	*Pisum sativum* L.	NA
PI 271035	Sweden	USDA	*Pisum sativum* L.	Improved cultivar
PI 271038	Nepal	USDA	*Pisum sativum* L.	Improved cultivar
PI 271116	China	USDA	*Pisum sativum* L.	Improved cultivar
PI 271511	India	USDA	*Pisum sativum* L.	NA
PI 272148	Finland	USDA	*Pisum sativum* L.	NA
PI 272171	Germany	USDA	*Pisum sativum* L.	NA
PI 272175	Germany	USDA	*Pisum sativum* L.	NA
PI 272184	Greece	USDA	*Pisum sativum* L.	NA
PI 272194	Germany	USDA	*Pisum sativum* L.	NA
PI 272215	Germany	USDA	*Pisum sativum* L.	NA
PI 272216	Bulgaria	USDA	*Pisum sativum* L.	NA
PI 272218	Poland	USDA	*Pisum sativum* L.	NA
PI 273209	Russia	USDA	*Pisum sativum* L. subsp. *elatius*	NA
PI 273605	Ecuador	USDA	*Pisum sativum* L.	Wild
PI 274307	Pakistan	USDA	*Pisum sativum* L.	Wild
PI 274308	Pakistan	USDA	*Pisum sativum* L.	Wild
PI 274584	Norway	USDA	*Pisum sativum* L.	NA
PI 275821	Sweden	USDA	*Pisum sativum* L.	NA
PI 275822	Sweden	USDA	*Pisum sativum* L.	NA
PI 275825	Sweden	USDA	*Pisum sativum* L.	NA
PI 277852	Ethiopia	USDA	*Pisum sativum* L.	NA
PI 279823	Germany	USDA	*Pisum sativum* L.	Improved cultivar
PI 279825	Germany	USDA	*Pisum sativum* L.	Improved cultivar
PI 280252	Ethiopia	USDA	*Pisum sativum* L.	NA
PI 280603	Israel	USDA	*Pisum sativum* L.	NA
PI 280609	Russia	USDA	*Pisum sativum* L.	NA
PI 280611	Ukraine	USDA	*Pisum sativum* L.	NA
PI 280613	Russia	USDA	*Pisum sativum* L.	NA
PI 280614	Russia	USDA	*Pisum sativum* L.	NA
PI 280616	Belarus	USDA	*Pisum sativum* L.	NA
PI 280617	Estonia	USDA	*Pisum sativum* L.	NA
PI 280619	Estonia	USDA	*Pisum sativum* L.	NA
PI 280626	Russia	USDA	*Pisum sativum* L.	NA
PI 285710	Poland	USDA	*Pisum sativum* L.	Improved cultivar
PI 285715	Poland	USDA	*Pisum sativum* L.	Improved cultivar
PI 285717	Poland	USDA	*Pisum sativum* L.	Improved cultivar
PI 285718	Poland	USDA	*Pisum sativum* L.	Improved cultivar
PI 285722	Poland	USDA	*Pisum sativum* L.	Improved cultivar
PI 285724	Poland	USDA	*Pisum sativum* L.	Improved cultivar
PI 285727	Poland	USDA	*Pisum sativum* L.	Improved cultivar
PI 285730	Poland	USDA	*Pisum sativum* L.	Improved cultivar
PI 285740	Poland	USDA	*Pisum sativum* L.	Improved cultivar
PI 285747	Poland	USDA	*Pisum sativum* L.	Improved cultivar
PI 286430	Nepal	USDA	*Pisum sativum* L.	NA
PI 286431	Nepal	USDA	*Pisum sativum* L.	NA
PI 286607	Thailand	USDA	*Pisum sativum* L.	NA
PI 288025	France	USDA	*Pisum sativum* L.	Improved cultivar
PI 293426	Bulgaria	USDA	*Pisum sativum* L.	NA
PI 306591	Hungary	USDA	*Pisum sativum* L.	Improved cultivar
PI 307666	Costa Rica	USDA	*Pisum sativum* L.	Traditional cultivar/Landrace
PI 308796	India	USDA	*Pisum sativum* L.	NA
PI 314794	Australia	USDA	*Pisum sativum* L.	NA
PI 314795	Australia	USDA	*Pisum sativum* L.	NA
PI 319374	Mexico	USDA	*Pisum sativum* L.	Traditional cultivar/Landrace
PI 320972	Hungary	USDA	*Pisum sativum* L.	NA
PI 324695	Hungary	USDA	*Pisum sativum* L.	Improved cultivar
PI 324697	Hungary	USDA	*Pisum sativum* L.	Improved cultivar
PI 324700	Hungary	USDA	*Pisum sativum* L.	Improved cultivar
PI 324702	Hungary	USDA	*Pisum sativum* L.	Improved cultivar
PI 324703	Hungary	USDA	*Pisum sativum* L.	Improved cultivar
PI 324706	Romania	USDA	*Pisum sativum* L.	NA
PI 331413	Ethiopia	USDA	*Pisum sativum* L.	NA
PI 331414	Ethiopia	USDA	*Pisum sativum* L.	NA
PI 340128	Turkey	USDA	*Pisum sativum* L.	NA
PI 340130	Turkey	USDA	*Pisum sativum* L.	NA
PI 343292	USA	USDA	*Pisum sativum* L.	NA
PI 343321	USA	USDA	*Pisum sativum* L.	NA
PI 343331	USA	USDA	*Pisum sativum* L.	NA
PI 343338	USA	USDA	*Pisum sativum* L.	NA
PI 343824	Uganda	USDA	*Pisum sativum* L.	NA
PI 343958	Turkey	USDA	*Pisum sativum* L.	NA
PI 343972	Turkey	USDA	*Pisum sativum* L. subsp. *elatius*	Wild
PI 343977	Turkey	USDA	*Pisum sativum* L. subsp. *elatius*	Wild
PI 343979	Turkey	USDA	*Pisum sativum* L. subsp. *elatius*	Wild
PI 343987	Turkey	USDA	*Pisum sativum* L. subsp. *sativum*	NA
PI 344003	Turkey	USDA	*Pisum sativum* L.	Wild
PI 344007	Greece	USDA	*Pisum sativum* L. subsp. *elatius*	Wild
PI 344009	Greece	USDA	*Pisum sativum* L. subsp. *elatius*	Wild
PI 344010	Greece	USDA	*Pisum sativum* L. subsp. *elatius*	Wild
PI 344011	Greece	USDA	*Pisum sativum* L. subsp. *elatius*	Wild
PI 344012	Greece	USDA	*Pisum sativum* L. subsp. *elatius*	Wild
PI 344013	Greece	USDA	*Pisum sativum* L. subsp. *elatius*	Wild
PI 344538	Italy	USDA	*Pisum sativum* L. subsp. *elatius*	NA
PI 347281	India	USDA	*Pisum sativum* L.	NA
PI 347295	India	USDA	*Pisum sativum* L.	NA
PI 347457	India	USDA	*Pisum sativum* L.	NA
PI 347477	India	USDA	*Pisum sativum* L.	NA
PI 347490	India	USDA	*Pisum sativum* L.	NA
PI 347496	India	USDA	*Pisum sativum* L.	NA
PI 355906	Japan	USDA	*Pisum sativum* L.	Improved cultivar
PI 356974	India	USDA	*Pisum sativum* L.	NA
PI 356980	India	USDA	*Pisum sativum* L.	NA
PI 356984	India	USDA	*Pisum sativum* L.	NA
PI 356986	India	USDA	*Pisum sativum* L.	NA
PI 356991	India	USDA	*Pisum sativum* L.	NA
PI 356992	India	USDA	*Pisum sativum* L.	NA
PI 357290	Macedonia	USDA	*Pisum sativum* L.	NA
PI 357292	Serbia	USDA	*Pisum sativum* L.	NA
PI 358300	Ethiopia	USDA	*Pisum sativum* L.	NA
PI 358620	Ethiopia	USDA	*Pisum sativum* L.	NA
PI 358633	Ethiopia	USDA	*Pisum sativum* L.	NA
PI 358640	Ethiopia	USDA	*Pisum sativum* L.	NA
PI 365419	Canada	USDA	*Pisum sativum* L.	Improved cultivar
PI 371796	New Zealand	USDA	*Pisum sativum* L.	NA
PI 378157	Malaysia	USDA	*Pisum sativum* L.	NA
PI 381334	Netherlands	USDA	*Pisum sativum* L.	Improved cultivar
PI 393488	Czech Republic	USDA	*Pisum sativum* L.	Improved cultivar
PI 393489	Czech Republic	USDA	*Pisum sativum* L.	Improved cultivar
PI 393490	Czech Republic	USDA	*Pisum sativum* L.	Improved cultivar
PI 404225	Belarus	USDA	*Pisum sativum* L.	Improved cultivar
PI 409031	Germany	USDA	*Pisum sativum* L.	Improved cultivar
PI 411141	New Zealand	USDA	*Pisum sativum* L.	Improved cultivar
PI 411142	New Zealand	USDA	*Pisum sativum* L.	Improved cultivar
PI 413678	Hungary	USDA	*Pisum sativum* L.	Improved cultivar
PI 413683	Hungary	USDA	*Pisum sativum* L.	Improved cultivar
PI 413685	Hungary	USDA	*Pisum sativum* L.	Improved cultivar
PI 413688	Hungary	USDA	*Pisum sativum* L.	Improved cultivar
PI 413698	Hungary	USDA	*Pisum sativum* L.	Improved cultivar
PI 413703	Hungary	USDA	*Pisum sativum* L.	Improved cultivar
PI 429839	Afghanistan	USDA	*Pisum sativum* L.	Improved cultivar
PI 429843	Latvia	USDA	*Pisum sativum* L.	Improved cultivar
PI 429845	Russia	USDA	*Pisum sativum* L.	Improved cultivar
PI 429849	Uzbekistan	USDA	*Pisum sativum* L.	Improved cultivar
PI 430702	Hungary	USDA	*Pisum sativum* L.	NA
PI 433560	Israel	USDA	*Pisum fulvum* Sm.	Wild
PI 476409	Latvia	USDA	*Pisum sativum* L.	NA
PI 476410	Ukraine	USDA	*Pisum sativum* L.	NA
PI 476413	Russia	USDA	*Pisum sativum* L.	NA
PI 477371	Denmark	USDA	*Pisum sativum* L.	Improved cultivar
PI 486131	Ecuador	USDA	*Pisum sativum* L.	NA
PI 494077	Chile	USDA	*Pisum sativum* L.	NA
PI 499982	China	USDA	*Pisum sativum* L.	NA
PI 505059	Sudan	USDA	*Pisum sativum* L. subsp. *elatius*	NA
PI 505062	Greece	USDA	*Pisum sativum* L. subsp. *sativum*	NA
PI 505080	Cyprus	USDA	*Pisum sativum* L. subsp. *sativum*	NA
PI 505108	Greece	USDA	*Pisum sativum* L. subsp. *sativum*	NA
PI 505122	Albania	USDA	*Pisum sativum* L. subsp. *sativum*	NA
PI 505127	Albania	USDA	*Pisum sativum* L. subsp. *sativum*	NA
PI 505144	Spain	USDA	*Pisum sativum* L. subsp. *sativum*	NA
PI 508092	USA	USDA	*Pisum sativum* L.	Improved cultivar
PI 531199	Israel	USDA	*Pisum fulvum* Sm.	Wild
PI 560055	Russia	USDA	*Pisum sativum* L. subsp. *elatius*	NA
PI 560056	Russia	USDA	*Pisum sativum* L. subsp. *elatius*	NA
PI 560058	Israel	USDA	*Pisum sativum* L. subsp. *elatius*	NA
PI 560061	Israel	USDA	*Pisum fulvum* Sm.	Wild
PI 560063 4	Israel	USDA	*Pisum fulvum* Sm.	Wild
PI 560064	Israel	USDA	*Pisum fulvum* Sm.	Wild
PI 560065	Israel	USDA	*Pisum fulvum* Sm.	Wild
PI 560066	Israel	USDA	*Pisum fulvum* Sm.	Wild
PI 560067 5	Israel	USDA	*Pisum fulvum* Sm.	Wild
PI 560069	Israel	USDA	*Pisum sativum* L. subsp. *elatius*	NA
PI 595932	Israel	USDA	*Pisum fulvum* Sm.	Wild
PI 595933 1	NA	USDA	*Pisum fulvum* Sm.	Wild
PI 595934	NA	USDA	*Pisum fulvum* Sm.	Wild
PI 595936	NA	USDA	*Pisum fulvum* Sm.	Wild
PI 595938	NA	USDA	*Pisum fulvum* Sm.	Wild
W6 44775	China	USDA	*Pisum sativum* L.	Traditional cultivar/Landrace
IG123102	Ethiopia	CREA	*Pisum sativum* L. subsp. *sativum*	Traditional cultivar/Landrace
IG49224	Georgia	CREA	*Pisum sativum* L.	Traditional cultivar/Landrace
PI 595943	NA	USDA	*Pisum fulvum* Sm.	Wild
IG51687	Greece	CREA	*Pisum sativum* L.	NA
PI 595948	NA	USDA	*Pisum fulvum* Sm.	Wild
PI 595953	NA	USDA	*Pisum fulvum* Sm.	Wild
PI 601426	USA	USDA	*Pisum sativum* L.	Improved cultivar
PI 601516	Netherlands	USDA	*Pisum sativum* L.	Improved cultivar
PI 614141	USA	USDA	*Pisum sativum* L.	Improved cultivar
PI 618586	USA	USDA	*Pisum sativum* L.	NA
PI 619079	USA	USDA	*Pisum sativum* L.	Improved cultivar
PI 639957	Turkey	USDA	*Pisum sativum* L. subsp. *elatius*	Wild
PI 639959	Turkey	USDA	*Pisum sativum* L. subsp. *elatius*	Wild
PI 639962	Turkmenistan	USDA	*Pisum sativum* L. subsp. *sativum*	Wild
PI 639964	Bulgaria	USDA	*Pisum sativum* L. subsp. *sativum*	Wild
PI 639967	India	USDA	*Pisum sativum* L. subsp. *sativum*	NA
PI 639968	Nepal	USDA	*Pisum sativum* L. subsp. *sativum*	NA
PI 639969	Nepal	USDA	*Pisum sativum* L. subsp. *sativum*	NA
PI 639974	Georgia	USDA	*Pisum sativum* L. subsp. *elatius*	NA
PI 639976	Bulgaria	USDA	*Pisum sativum* L. subsp. *sativum*	NA
PI 639977	Bulgaria	USDA	*Pisum sativum* L. subsp. *sativum*	NA
PI 639980	Bulgaria	USDA	*Pisum sativum* L. subsp. *sativum*	NA
PI 639981	Bulgaria	USDA	*Pisum sativum* L. subsp. *sativum*	NA
PI 664469	USA	USDA	*Pisum sativum* L.	Improved cultivar
PS0010128	USA	USDA	*Pisum sativum* L.	NA
PS0010946	USA	USDA	*Pisum sativum* L.	NA
PS02101137	USA	USDA	*Pisum sativum* L.	NA
PS03101445	USA	USDA	*Pisum sativum* L.	NA
PS03101822	USA	USDA	*Pisum sativum* L.	NA
PS04100462	USA	USDA	*Pisum sativum* L.	NA
PS04100710	USA	USDA	*Pisum sativum* L.	NA
PS05100120	USA	USDA	*Pisum sativum* L.	NA
PS05100522	USA	USDA	*Pisum sativum* L.	NA
PS05100632	USA	USDA	*Pisum sativum* L.	NA
PS05100735	USA	USDA	*Pisum sativum* L.	NA
PS05100736	USA	USDA	*Pisum sativum* L.	NA
PS05100840	USA	USDA	*Pisum sativum* L.	NA
PS05101142	USA	USDA	*Pisum sativum* L.	NA
PS05101240	USA	USDA	*Pisum sativum* L.	NA
PS06100490	USA	USDA	*Pisum sativum* L.	NA
PS06100542	USA	USDA	*Pisum sativum* L.	NA
PS06100617	USA	USDA	*Pisum sativum* L.	NA
PS06100760	USA	USDA	*Pisum sativum* L.	NA
PS06101004	USA	USDA	*Pisum sativum* L.	NA
PS06101043	USA	USDA	*Pisum sativum* L.	NA
PS06101119	USA	USDA	*Pisum sativum* L.	NA
PS06101338	USA	USDA	*Pisum sativum* L.	NA
PS06310024W	USA	USDA	*Pisum sativum* L.	NA
PS07100170	USA	USDA	*Pisum sativum* L.	NA
PS07100396	USA	USDA	*Pisum sativum* L.	NA
PS07100470	USA	USDA	*Pisum sativum* L.	NA
PS07100471	USA	USDA	*Pisum sativum* L.	NA
PS07100474	USA	USDA	*Pisum sativum* L.	NA
PS07100480	USA	USDA	*Pisum sativum* L.	NA
PS07100914	USA	USDA	*Pisum sativum* L.	NA
S1047	USA	USDA	*Pisum sativum* L.	NA
S1081	USA	USDA	*Pisum sativum* L.	NA
S1086	USA	USDA	*Pisum sativum* L.	NA
S1120 6	USA	USDA	*Pisum sativum* L.	NA
S1188	USA	USDA	*Pisum sativum* L.	NA
S1195	USA	USDA	*Pisum sativum* L.	NA
S1208	USA	USDA	*Pisum sativum* L.	NA
S1306	USA	USDA	*Pisum sativum* L.	NA
S1364 4	USA	USDA	*Pisum sativum* L.	NA
S1397	USA	USDA	*Pisum sativum* L.	NA
S1430	USA	USDA	*Pisum sativum* L.	NA
S1431	USA	USDA	*Pisum sativum* L.	NA
S1432	USA	USDA	*Pisum sativum* L.	NA
S1456	USA	USDA	*Pisum sativum* L.	NA
S1516	USA	USDA	*Pisum sativum* L.	NA
S1544	USA	USDA	*Pisum sativum* L.	NA
S1553	USA	USDA	*Pisum sativum* L.	NA
S1558	USA	USDA	*Pisum sativum* L.	NA
S1561	USA	USDA	*Pisum sativum* L.	NA
S1573	USA	USDA	*Pisum sativum* L.	NA
S158	USA	USDA	*Pisum sativum* L.	NA
S1586	USA	USDA	*Pisum sativum* L.	NA
S1587	USA	USDA	*Pisum sativum* L.	NA
S1591	USA	USDA	*Pisum sativum* L.	NA
S718	USA	USDA	*Pisum sativum* L.	NA
S859	USA	USDA	*Pisum sativum* L.	NA
S875 1	USA	USDA	*Pisum sativum* L.	NA
S906	USA	USDA	*Pisum sativum* L.	NA
S947	USA	USDA	*Pisum sativum* L.	NA
S973	USA	USDA	*Pisum sativum* L.	NA
W6 10096	Czech Republic	USDA	*Pisum sativum* L.	NA
W6 10925	Bulgaria	USDA	*Pisum sativum* L. subsp. *elatius*	Wild
W6 12723	Bulgaria	USDA	*Pisum sativum* L. subsp. *sativum*	Improved cultivar
W6 12738	NA	USDA	*Pisum sativum* L. subsp. *sativum*	NA
W6 12739	Bulgaria	USDA	*Pisum sativum* L. subsp. *sativum*	Improved cultivar
W6 15008	Israel	USDA	*Pisum sativum* L. subsp. *elatius*	NA
W6 15009	Georgia	USDA	*Pisum sativum* L. subsp. *elatius*	NA
W6 15010	Latvia	USDA	*Pisum sativum* L. subsp. *elatius*	NA
W6 15019	Turkey	USDA	*Pisum sativum* L. subsp. *elatius*	NA
W6 15028	NA	USDA	*Pisum sativum* L. subsp. *sativum*	NA
W6 15043	NA	USDA	*Pisum sativum* L. subsp. *elatius*	NA
W6 15044	NA	USDA	*Pisum sativum* L. subsp. *elatius*	NA
W6 15045	NA	USDA	*Pisum fulvum* Sm.	NA
IG51688	Greece	CREA	*Pisum sativum* L.	NA
W6 15047	NA	USDA	*Pisum sativum* L. subsp. *elatius*	NA
W6 15048	NA	USDA	*Pisum sativum* L. subsp. *elatius*	NA
W6 15163	USA	USDA	*Pisum sativum* L. subsp. *sativum*	NA
W6 17293	Pakistan	USDA	*Pisum sativum* L. subsp. *sativum*	Traditional cultivar/Landrace
W6 20025	Bulgaria	USDA	*Pisum* spp.	Wild
W6 20026	Bulgaria	USDA	*Pisum* spp.	Wild
W6 24570	Turkmenistan	USDA	*Pisum sativum* L. subsp. *sativum*	NA
W6 26109	Georgia	USDA	*Pisum sativum* L. subsp. *elatius*	Wild
W6 26127	Georgia	USDA	*Pisum sativum* L. subsp. *elatius*	Wild
W6 26154	Georgia	USDA	*Pisum sativum* L. subsp. *sativum*	Wild
W6 26157	Georgia	USDA	*Pisum sativum* L. subsp. *sativum*	Wild
W6 26160	Georgia	USDA	*Pisum sativum* L. subsp. *sativum*	Wild
W6 26161	Georgia	USDA	*Pisum sativum* L. subsp. *sativum*	Wild
W6 31707	Russia	USDA	*Pisum sativum* L. subsp. *sativum*	NA
W6 34960	United Kingdom	USDA	*Pisum sativum* L.	NA
W6 39729	USA	USDA	*Pisum sativum* L.	NA
IG51976	India	CREA	*Pisum sativum* L.	NA
IG51989	India	CREA	*Pisum sativum* L.	NA
W6 44574	China	USDA	*Pisum sativum* L.	Traditional cultivar/Landrace
W6 44578	China	USDA	*Pisum sativum* L.	Traditional cultivar/Landrace
PI 595944	Israel	USDA	*Pisum fulvum* Sm.	Wild
IG52459	Morocco	CREA	*Pisum sativum* L.	Wild
W6 44581	China	USDA	*Pisum sativum* L.	Traditional cultivar/Landrace
PI 595939	NA	USDA	*Pisum fulvum* Sm.	Wild
W6 44583	China	USDA	*Pisum sativum* L.	Traditional cultivar/Landrace
W6 44642	China	USDA	*Pisum sativum* L.	Traditional cultivar/Landrace
PI 595940	NA	USDA	*Pisum fulvum* Sm.	Wild
W6 44712	China	USDA	*Pisum sativum* L.	Traditional cultivar/Landrace
W6 44713	China	USDA	*Pisum sativum* L.	Traditional cultivar/Landrace
W6 44714	China	USDA	*Pisum sativum* L.	Traditional cultivar/Landrace
W6 44715	China	USDA	*Pisum sativum* L.	Traditional cultivar/Landrace
W6 44716	China	USDA	*Pisum sativum* L.	Traditional cultivar/Landrace
W6 44717	China	USDA	*Pisum sativum* L.	Traditional cultivar/Landrace
W6 44718	China	USDA	*Pisum sativum* L.	Traditional cultivar/Landrace
PI 595941	NA	USDA	*Pisum fulvum* Sm.	Wild
W6 44720	China	USDA	*Pisum sativum* L.	Traditional cultivar/Landrace
W6 15046	NA	USDA	*Pisum fulvum* Sm.	NA
IG115145	Nepal	CREA	*Pisum sativum* L.	Traditional cultivar/Landrace
W6 44723	China	USDA	*Pisum sativum* L.	Traditional cultivar/Landrace
W6 44724	China	USDA	*Pisum sativum* L.	Traditional cultivar/Landrace
ZP0064	Spain	CREA	*Pisum sativum* L.	Traditional cultivar/Landrace
W6 44726	China	USDA	*Pisum sativum* L.	Traditional cultivar/Landrace
W6 44765	China	USDA	*Pisum sativum* L.	Traditional cultivar/Landrace
W6 44766	China	USDA	*Pisum sativum* L.	Traditional cultivar/Landrace
IFPI 3232	Syria	USDA	*Pisum fulvum* Sm.	NA
W6 44768	China	USDA	*Pisum sativum* L.	Traditional cultivar/Landrace
W6 44769	China	USDA	*Pisum sativum* L.	Traditional cultivar/Landrace
W6 44770	China	USDA	*Pisum sativum* L.	Traditional cultivar/Landrace
IFPI 3260	Syria	USDA	*Pisum fulvum* Sm.	NA
P660 4	Syria	USDA	*Pisum fulvum* Sm.	NA
IG50362	Turkey	CREA	*Pisum sativum* L.	NA

Table S3. Composition of the 38 duplication groups (DGs) identified using the pairwise identity by state (IBS) threshold of 0.99. DGs in which accessions from CREA and USDA collections are grouped together are marked in red.

DG	Genotype	Origin	Collection
1	PI 286431	Nepal	USDA
	PI 271511	India	USDA
	IG115341	Nepal	CREA
	IG115100	Nepal	CREA
	IG51957	India	CREA
	IG51948	India	CREA
2	W6 15045	NA	USDA
	PI 595948	NA	USDA
	PI 595936	NA	USDA
	PI 595932	Israel	USDA
3	W6 44716	China	USDA
	W6 44715	China	USDA
	IG52383	China	CREA
4	W6 15047	NA	USDA
	PI 344010	Greece	USDA
	PI 269762	United Kingdom	USDA
5	PS05100736	USA	USDA
	PS05100735	USA	USDA
	PS04100462	USA	USDA
6	PI 413688	Hungary	USDA
	PI 413678	Hungary	USDA
	PI 280611	Ukraine	USDA
7	PI 357292	Serbia	USDA
	PI 356992	India	USDA
	PI 356991	India	USDA
8	PI 347281	India	USDA
	PI 163129	India	USDA
	IG51891	India	CREA
9	PI 319374	Mexico	USDA
	PI 314794	Australia	USDA
	PI 271035	Sweden	USDA
10	PI 250448	Czech Republic	USDA
	PI 250440	Czech Republic	USDA
	PI 250438	Czech Republic	USDA
11	IG52081	Ethiopia	CREA
	IG51516	Ethiopia	CREA
	IG49176	Ethiopia	CREA
12	PI 285724	Poland	USDA
	PI 250447	Czech Republic	USDA
13	Witham Wonder	United Kingdom	CREA
	PI 275822	Sweden	USDA
14	W6 44770	China	USDA
	W6 44765	China	USDA
15	W6 26161	Georgia	USDA
	W6 26157	Georgia	USDA
16	W6 26160	Georgia	USDA
	W6 26154	Georgia	USDA
17	W6 24570	Turkmenistan	USDA
	PI 639962	Turkmenistan	USDA
18	W6 15043	NA	USDA
	PI 269777	United Kingdom	USDA
19	S1195	USA	USDA
	S1188	USA	USDA
20	S1086	USA	USDA
	S1081	USA	USDA
21	PS07100480	USA	USDA
	PS07100470	USA	USDA
22	PI 639980	Bulgaria	USDA
	PI 639976	Bulgaria	USDA
23	PI 595953	NA	USDA
	PI 595938	NA	USDA
24	PI 595934	NA	USDA
	PI 595933 1	NA	USDA
25	PI 393488	Czech Republic	USDA
	PI 357290	Macedonia	USDA
26	PI 347477	India	USDA
	PI 347457	India	USDA
27	PI 343958	Turkey	USDA
	PI 343824	Uganda	USDA
28	PI 324706	Romania	USDA
	PI 206006	Sweden	USDA
29	PI 286607	Thailand	USDA
	PI 257244	China	USDA
30	PI 271116	China	USDA
	IG49633	Greece	CREA
31	PI 271038	Nepal	USDA
	Landrace Orava	Czech Republic	CREA
32	PI 269778	United Kingdom	USDA
	Fillbasket	United Kingdom	CREA
33	PI 263871	Greece	USDA
	PI 261671	Netherlands	USDA
34	PI 220174	Afghanistan	USDA
	PI 212031	Iran	USDA
35	PI 210568	Finland	USDA
	PI 203066	Finland	USDA
36	PI 195631	Ethiopia	USDA
	PI 195404	Guatemala	USDA
37	PI 180699	Germany	USDA
	PI 180696	Germany	USDA
38	PI 175231	Nepal	USDA
	PI 174921	Nepal	USDA

Table 54. Composition of the 81 similarity groups (SGs) identified using the pairwise identity by state (IBS) threshold of 0.95. SGs in which accessions from CREA and USDA collections are grouped together are marked in red. The accessions with the highest call rate, chosen to represent each SG, are highlighted in bold.

SG	Genotype	Origin	Collection
1	S1081	USA	USDA
	S1086	USA	USDA
	S1120_6	USA	USDA
	S1188	USA	USDA
	**S1195**	USA	USDA
	S1208	USA	USDA
	S859	USA	USDA
	S973	USA	USDA
2	IG115100	Nepal	CREA
	IG115266	Nepal	CREA
	IG115341	Nepal	CREA
	IG51948	India	CREA
	**IG51957**	India	CREA
	PI_271511	India	USDA
	PI_286431	Nepal	USDA
3	PI_560061	Israel	USDA
	PI_560067_5	Israel	USDA
	PI_595932	Israel	USDA
	PI_595936	NA	USDA
	PI_595948	NA	USDA
	**W6_15045**	NA	USDA
4	IG49189	Afghanistan	CREA
	IG49203	Afghanistan	CREA
	PI_116944	Afghanistan	USDA
	**PI_212031**	Iran	USDA
	PI_220174	Afghanistan	USDA
	PI_222071	Afghanistan	USDA
5	IG128983	Russia	CREA
	IG134857	Bulgaria	CREA
	PI_280611	Ukraine	USDA
	**PI_413678**	Hungary	USDA
	PI_413688	Hungary	USDA
6	Kiflica	Serbia	USDA
	**PI_250444**	Czech Republic	USDA
	PI_261636	Spain	USDA
	PI_413683	Hungary	USDA
7	**Fillbasket**	United Kingdom	CREA
	PI_269778	United Kingdom	USDA
	PI_411142	New Zealand	USDA
	PI_618586	USA	USDA
8	**IG52383**	China	CREA
	W6_44714	China	USDA
	W6_44715	China	USDA
	W6_44716	China	USDA
9	**PI_242027**	Denmark	USDA
	PI_269762	United Kingdom	USDA
	PI_344010	Greece	USDA
	W6_15047	NA	USDA
10	S1516	USA	USDA
	S1553	USA	USDA
	**S1558**	USA	USDA
	S1561	USA	USDA
11	PI_197044	Honduras	USDA
	PI_271035	Sweden	USDA
	**PI_314794**	Australia	USDA
	PI_319374	Mexico	USDA
12	IG125589	Kyrgyzstan	CREA
	IG134782	Russia	CREA
	**IG134870**	Bulgaria	CREA
13	PI_250447	Czech Republic	USDA
	PI_285724	Poland	USDA
	**ZP1282**	Spain	CREA
14	Carneval	Sweden	USDA
	PS06101338	USA	USDA
	**W6_10096**	Czech Republic	USDA
15	**Cascadia**	USA	USDA
	OR_Giant	USA	USDA
	S718	USA	USDA
16	S1364_4	USA	USDA
	S1397	USA	USDA
	**S1591**	USA	USDA
17	**English_Wonder**	United Kingdom	CREA
	PI_275822	Sweden	USDA
	Witham_Wonder	United Kingdom	CREA
18	**M194_1**	USA	USDA
	OSPII	USA	USDA
	S158	USA	USDA
19	PS04100462	USA	USDA
	PS05100735	USA	USDA
	**PS05100736**	USA	USDA
20	**PI_531199**	Israel	USDA
	PI_560064	Israel	USDA
	PI_560066	Israel	USDA
21	PI_358620	Ethiopia	USDA
	PI_358640	Ethiopia	USDA
	**PI_505080**	Cyprus	USDA
22	**PI_356991**	India	USDA
	PI_356992	India	USDA
	PI_357292	Serbia	USDA
23	IG51891	India	CREA
	**PI_163129**	India	USDA
	PI_347281	India	USDA
24	**Kentish_Invicta**	United Kingdom	CREA
	PI_274584	Norway	USDA
	PI_285727	Poland	USDA
25	**Alderman**	United Kingdom	CREA
	Landrace_Orava	Czech Republic	CREA
	PI_271038	Nepal	USDA
26	PI_250438	Czech Republic	USDA
	**PI_250440**	Czech Republic	USDA
	PI_250448	Czech Republic	USDA
27	**IG49176**	Ethiopia	CREA
	IG51516	Ethiopia	CREA
	IG52081	Ethiopia	CREA
28	**PI_261622**	Spain	USDA
	PI_320972	Hungary	USDA
29	**PI_411141**	New Zealand	USDA
	ZP1300	Spain	CREA
30	**PI_263032**	France	USDA
	PI_664469	USA	USDA
31	**Kapucin_Belokvety**	Czech Republic	CREA
	PI_381334	Netherlands	USDA
32	**PI_250439**	Czech Republic	USDA
	PI_285747	Poland	USDA
33	W6_44765	China	USDA
	**W6_44770**	China	USDA
34	**W6_44578**	China	USDA
	W6_44720	China	USDA
35	PS05100120	USA	USDA
	**W6_39729**	USA	USDA
36	W6_26157	Georgia	USDA
	**W6_26161**	Georgia	USDA
37	**W6_26154**	Georgia	USDA
	W6_26160	Georgia	USDA
38	W6_26109	Georgia	USDA
	**W6_26127**	Georgia	USDA
39	PI_639962	Turkmenistan	USDA
	**W6_24570**	Turkmenistan	USDA
40	**W6_15019**	Turkey	USDA
	W6_15044	NA	USDA
41	PI_269777	United Kingdom	USDA
	**W6_15043**	NA	USDA
42	**S1430**	USA	USDA
	S1432	USA	USDA
43	S1306	USA	USDA
	**S947**	USA	USDA
44	PS07100470	USA	USDA
	**PS07100480**	USA	USDA
45	PS0010946	USA	USDA
	**PS07100170**	USA	USDA
46	PS06101004	USA	USDA
	**PS06101043**	USA	USDA
47	PI_639976	Bulgaria	USDA
	**PI_639980**	Bulgaria	USDA
48	PI_595938	NA	USDA
	**PI_595953**	NA	USDA
49	PI_595933_1	NA	USDA
	**PI_595934**	NA	USDA
50	**PI_180329**	India	USDA
	PI_505059	Sudan	USDA
51	PI_269822	United Kingdom	USDA
	**PI_476410**	Ukraine	USDA
52	**IG134609**	Georgia	CREA
	PI_476409	Latvia	USDA
53	**IG52586**	Algeria	CREA
	PI_413698	Hungary	USDA
54	**PI_357290**	Macedonia	USDA
	PI_393488	Czech Republic	USDA
55	**PI_172339**	Netherlands	USDA
	PI_355906	Japan	USDA
56	**PI_347457**	India	USDA
	PI_347477	India	USDA
57	PI_344012	Greece	USDA
	**PI_344013**	Greece	USDA
58	PI_343824	Uganda	USDA
	**PI_343958**	Turkey	USDA
59	**PI_206006**	Sweden	USDA
	PI_324706	Romania	USDA
60	PI_257244	China	USDA
	**PI_286607**	Thailand	USDA
61	PI_279823	Germany	USDA
	**PI_285740**	Poland	USDA
62	**PI_261624**	Spain	USDA
	PI_285730	Poland	USDA
63	**PI_274307**	Pakistan	USDA
	PI_274308	Pakistan	USDA
64	IG49633	Greece	CREA
	**PI_271116**	China	USDA
65	**PI_155109**	USA	USDA
	PI_269802	United Kingdom	USDA
66	**PI_261671**	Netherlands	USDA
	PI_263871	Greece	USDA
67	Chemin_Long	France	CREA
	**PI_263030**	France	USDA
68	Englishsabel	United Kingdom	CREA
	**PI_263014**	Netherlands	USDA
69	PI_179970	India	USDA
	**PI_250446**	Czech Republic	USDA
70	**PI_203069**	Finland	USDA
	PI_210571	Finland	USDA
71	**PI_203066**	Finland	USDA
	PI_210568	Finland	USDA
72	**PI_195404**	Guatemala	USDA
	PI_195631	Ethiopia	USDA
73	PI_180696	Germany	USDA
	**PI_180699**	Germany	USDA
74	IG115114	Nepal	CREA
	**PI_179722**	India	USDA
75	PI_174921	Nepal	USDA
	**PI_175231**	Nepal	USDA
76	IG50235	India	CREA
	**PI_164548**	India	USDA
77	**PI_137118**	Canada	USDA
	PI_137119	Canada	USDA
78	IG116455	Turkey	CREA
	**PIS_845**	Germany	CREA
79	**IG52534**	Syria	CREA
	IG52535	Syria	CREA
80	IG52417	Syria	CREA
	**IG52442**	Syria	CREA
81	**IG50250**	Turkey	CREA
	IG51927	India	CREA

Table S5. Coordinates on the top three axes of accessions subjected to principal component analysis.

Accession	PC1(%20.37)	PC2(%14.14)	PC3(%11.67)
A778_26_6-USA	-0,0121	0,0357	-0,0249
Alderman-UK(SG25)	0,0466	0,0214	-0,0175
Attika-FRA	0,0415	0,0095	-0,0215
Cartouche-FRA	0,0192	-0,0506	0,0096
Cascadia-USA(SG15)	0,0379	0,0223	-0,0062
Champagne-FRA	-0,0312	-0,1728	0,0700
Cigarron-SPA	0,0291	-0,0061	-0,0095
CL_19cvs1-CSK	0,0185	-0,0055	-0,0166
Cote_D'Or-FRA	-0,0320	-0,1699	0,0769
Dove-FRA	0,0152	-0,0266	0,0074
Emerald_Gem-UK	0,0346	0,0162	-0,0076
Enduro-FRA	0,0208	-0,0356	-0,0008
English_Maple-UK	-0,0001	-0,0095	-0,0205
English_Wonder-UK(SG17)	0,0450	0,0235	-0,0154
Fillbasket-UK(SG7)	0,0415	0,0214	-0,0103
Genial_(PSC0073)-FRA	0,0368	0,0114	-0,0182
Gloire_de_Correze-FRA	0,0206	0,0060	-0,0175
Gris_de_Bourgogne-FRA	-0,0208	-0,1063	0,0295
Haute_Loire-FRA	-0,0240	-0,1248	0,0438
Hrach_Z_Pardubic-CSK	0,0277	0,0001	-0,0149
IG112140-MOR	-0,0553	-0,0267	-0,0468
IG114899-NPL	0,0100	0,0069	-0,0070
IG114914-NPL	0,0254	0,0136	-0,0053
IG114977-NPL	0,0131	0,0263	0,0246
IG115228-NPL	0,0258	0,0115	-0,0152
IG115331-NPL	0,0050	0,0275	0,0290
IG116232-TUR	-0,0257	0,0122	0,0194
IG116297-TUR	-0,0028	-0,0982	0,0335
IG122966-TKM	0,0057	-0,0089	-0,0121
IG122974-TJK	-0,0099	-0,0195	-0,0166
IG122996-KAZ	-0,0071	-0,0166	-0,0139
IG123004-TUN	0,0371	0,0100	-0,0165
IG123006-ETH	0,0272	0,0068	-0,0171
IG123021-AFG	0,0047	-0,0029	0,0011
IG123028-AFG	-0,0301	-0,0800	0,0341
IG123029-AFG	-0,0088	0,0163	0,0179
IG123034-AFG	-0,0012	0,0320	0,0372
IG123041-KAZ	0,0131	-0,0055	-0,0177
IG123050-TUR	0,0058	0,0021	0,0001
IG123073-DZA	-0,0656	-0,0513	-0,0504
IG123080-PAL	-0,0219	-0,0278	-0,0163
IG123084-PAL	-0,0091	-0,0054	-0,0166
IG123085-LBN	-0,0443	-0,0047	-0,0315
IG123118-ETH	0,0239	-0,0048	-0,0101
IG123136-ETH	0,0277	0,0124	-0,0167
IG123211-CHN	0,0182	0,0309	0,0266
IG123227-CHN	-0,0239	0,0219	0,0504
IG123237-CHN	-0,0545	0,0743	0,1455
IG123240-CHN	-0,0399	0,0666	0,1211
IG123242-CHN	-0,0497	0,0720	0,1427
IG123244-CHN	0,0075	0,0194	0,0150
IG123245-CHN	-0,0440	-0,0439	0,0462
IG123248-CHN	-0,0100	0,0353	0,0538
IG123280-UZB	-0,0120	-0,0223	-0,0213
IG123281-UZB	-0,0067	-0,0183	-0,0060
IG123288-UZB	-0,0093	-0,0407	-0,0084
IG123311-KAZ	-0,0053	-0,0083	0,0019
IG123312-TJK	-0,0073	0,0143	0,0273
IG123313-MOR	-0,0912	-0,0542	-0,0737
IG124664-CHN	0,0184	0,0176	0,0202
IG124843-UKR	0,0297	0,0070	-0,0132
IG124857-AFG	-0,0596	0,0235	0,0392
IG125324-RUS	0,0247	0,0068	-0,0135
IG125326-RUS	0,0231	0,0021	-0,0156
IG125336-UKR	0,0196	-0,0024	-0,0130
IG125378-RUS	-0,0342	-0,0101	0,0048
IG125415-RUS	-0,0098	-0,0101	-0,0127
IG125469-GEO	-0,0746	-0,0092	0,0154
IG125471-GEO	-0,0849	-0,0024	0,0120
IG125472-GEO	-0,0742	-0,0159	0,0009
IG125543-TJK	-0,0325	-0,0337	-0,0270
IG125550-CHN	0,0009	0,0152	0,0256
IG125597-TJK	-0,0365	0,0088	0,0485
IG125600-TJK	-0,0325	0,0093	0,0499
IG128856-GEO	0,0119	-0,0010	-0,0230
IG128863-UKR	-0,0068	-0,0118	-0,0179
IG128887-RUS	-0,0294	-0,0249	-0,0142
IG128913-UKR	0,0187	0,0006	-0,0181
IG128934-UKR	-0,0089	-0,0121	-0,0109
IG128973-BGR	0,0065	0,0016	-0,0124
IG129002-GRC	-0,0028	-0,0478	-0,0066
IG134060-ROM	-0,0051	-0,0810	0,0122
IG134080-ROM	-0,0207	-0,1307	0,0447
IG134094-ROM	-0,0193	-0,1248	0,0456
IG134109-ROM	-0,0269	-0,1018	0,0228
IG134609-GEO(SG52)	-0,0002	-0,0197	-0,0225
IG134619-RUS	0,0332	0,0034	-0,0163
IG134621-RUS	0,0332	0,0105	-0,0116
IG134649-UKR	0,0221	0,0008	-0,0174
IG134707-GEO	-0,0189	-0,0235	-0,0138
IG134718-GEO	-0,0091	-0,1042	0,0344
IG134744-UKR	0,0141	-0,0006	-0,0155
IG134746-UKR	0,0174	-0,0092	-0,0167
IG134750-UKR	0,0149	-0,0025	-0,0103
IG134770-UKR	0,0323	0,0088	-0,0160
IG134772-UKR	0,0317	0,0014	-0,0164
IG134788-RUS	0,0259	0,0132	-0,0015
IG134823-RUS	0,0144	-0,0433	0,0109
IG134828-GEO	-0,0067	-0,0103	-0,0154
IG134862-BGR	0,0247	0,0031	-0,0153
IG134870-BGR(SG12)	0,0116	-0,0165	-0,0166
IG49176-ETH(SG27)	0,0297	0,0135	-0,0080
IG49327-GRC	-0,0332	-0,1643	0,0560
IG49544-GEO	-0,0497	-0,0411	0,0292
IG49610-GRC	-0,0159	-0,0883	0,0059
IG50248-TUR	-0,0827	-0,0132	-0,1014
IG50250-TUR(SG81)	0,0287	0,0173	-0,0077
IG50303-IND	0,0262	0,0044	-0,0213
IG50311-TUR	0,0261	0,0002	-0,0202
IG50357-TUR	-0,1521	-0,0165	-0,1678
IG50358-TUR	0,0227	0,0008	-0,0254
IG50559-GRC	-0,0047	-0,0721	0,0179
IG50570-GRC	0,0005	-0,0316	0,0195
IG50584-GRC	0,0024	-0,0454	0,0040
IG50592-CHN	0,0123	0,0227	0,0223
IG50641-GRC	-0,0424	-0,1637	0,0539
IG50669-CHN	0,0156	0,0087	0,0063
IG50673-GRC	-0,0169	-0,0354	0,0486
IG50756-GRC	0,0249	0,0079	-0,0130
IG50935-GRC	0,0236	0,0050	-0,0147
IG51513-ETH	0,0214	0,0050	-0,0114
IG51520-ETH	0,0203	0,0107	-0,0060
IG51529-ETH	0,0215	0,0063	-0,0024
IG51536-ETH	0,0070	-0,0320	-0,0024
IG51551-ETH	0,0321	0,0102	-0,0162
IG51562-ETH	0,0309	0,0060	-0,0211
IG51576-ETH	0,0246	0,0028	-0,0196
IG51669-GRC	-0,0030	-0,0437	0,0114
IG51957-IND(SG2)	0,0322	0,0100	-0,0131
IG51991-IND	0,0186	0,0123	0,0005
IG51993-IND	0,0167	0,0073	-0,0087
IG51994-IND	0,0107	0,0076	0,0121
IG52005-IND	0,0268	0,0136	-0,0088
IG52017-IND	0,0182	0,0033	-0,0088
IG52036-CHN	0,0248	0,0027	-0,0162
IG52040-CHN	-0,0293	0,0594	0,1120
IG52050-IND	-0,0017	-0,0206	-0,0058
IG52092-ETH	0,0285	0,0103	-0,0110
IG52383-CHN(SG8)	0,0112	0,0317	0,0358
IG52401-SYR	-0,0366	0,0024	-0,0315
IG52426-SYR	-0,1343	-0,0183	-0,1515
IG52442-SYR(SG80)	-0,1671	-0,0162	-0,1831
IG52455-MOR	0,0104	-0,0028	-0,0308
IG52456-MOR	-0,0129	-0,0759	0,0148
IG52496-TUR	-0,1197	0,0764	0,0808
IG52521-TUR	-0,1579	-0,0192	-0,1654
IG52534-SYR(SG79)	0,0021	-0,0239	-0,0280
IG52586-DZA(SG53)	0,0455	0,0221	-0,0134
IG52595-DZA	-0,0794	-0,0504	-0,0642
IG52596-DZA	-0,0834	-0,0508	-0,0689
Isard-FRA	0,0216	-0,0374	0,0081
Kapucin_Belokvety-CSK(SG31)	0,0348	0,0118	-0,0174
Kentish_Invicta-UK(SG24)	0,0397	0,0199	-0,0108
Knights_Dwarf_White-UK	0,0393	0,0168	-0,0150
Knights_Marrow-UK	0,0424	0,0186	-0,0117
Kocovska_108-CSK	-0,0166	-0,0234	-0,0324
M193-USA	0,0415	0,0209	-0,0076
M194_1-USA(SG18)	0,0328	0,0174	-0,0087
Magnum_Bonum-UK	0,0318	0,0080	-0,0147
Messire-FRA	0,0319	0,0099	-0,0139
MG_100948-ITA	0,0181	0,0008	-0,0099
MG_101126-ITA	0,0189	0,0033	-0,0268
MG_106069-ITA	0,0259	0,0126	-0,0060
MG_106871-ITA	0,0197	-0,0040	-0,0146
MG_110243-ITA	0,0129	0,0047	-0,0192
MG_110416-ITA	0,0023	-0,0094	-0,0279
MG_110417-ITA	0,0036	-0,0107	-0,0216
MG_110418-ITA	0,0016	-0,0040	-0,0244
MG_111850-ITA	0,0025	-0,0119	-0,0158
MG_111988-ITA	-0,0040	-0,0200	-0,0239
MG_115084-ITA	0,0018	-0,0014	-0,0168
Mummy_Pea-UK	0,0356	0,0102	-0,0181
PI_102888-CHN	-0,0417	0,0584	0,1215
PI_103058-CHN	0,0141	0,0344	0,0340
PI_109866-VEN	0,0017	-0,0134	-0,0224
PI_116056-IND	0,0218	0,0170	0,0029
PI_116844-PAK	0,0352	0,0198	-0,0079
PI_117264-TUR	0,0132	0,0002	-0,0159
PI_117998-BRA	0,0011	-0,0148	-0,0253
PI_118501-BRA	0,0261	0,0036	-0,0191
PI_121352-IND	0,0308	0,0130	-0,0146
PI_124478-PAK	0,0331	0,0078	-0,0115
PI_125839-AFG	-0,0624	0,0211	0,0734
PI_125840-AFG	-0,0572	0,0467	0,1085
PI_134271-AFG	-0,0298	0,0554	0,1152
PI_137118-CAN(SG77)	0,0272	0,0146	-0,0106
PI_142775-MEX	0,0267	0,0063	-0,0226
PI_143485-AZE	-0,0541	0,0393	0,0608
PI_155109-USA(SG65)	0,0227	0,0030	-0,0132
PI_156647-ETH	0,0200	0,0036	-0,0145
PI_156720-JPN	0,0261	0,0117	-0,0124
PI_162909-PRY	0,0256	0,0273	0,0099
PI_163126-IND	0,0076	0,0383	0,0512
PI_163129-IND(SG23)	0,0294	0,0218	0,0053
PI_164548-IND(SG76)	0,0301	0,0248	0,0022
PI_164612-IND	0,0176	0,0039	-0,0067
PI_164779-IND	-0,0477	0,0705	0,1425
PI_164971-TUR	0,0271	0,0045	-0,0204
PI_164972-TUR	-0,0088	-0,0055	0,0040
PI_165949-IND	-0,0457	0,0671	0,1399
PI_166084-IND	-0,0259	0,0583	0,1046
PI_166159-NPL	-0,0507	0,0741	0,1619
PI_169608-TUR	0,0309	0,0014	-0,0208
PI_172339-NLD(SG55)	0,0416	0,0109	-0,0226
PI_173840-IND	0,0294	0,0076	-0,0192
PI_175231-NPL(SG75)	-0,0477	0,0721	0,1468
PI_179449-TUR	-0,0694	-0,0063	0,0178
PI_179450-SYR	0,0255	0,0072	-0,0151
PI_179451-SYR	0,0044	-0,0091	-0,0122
PI_179459-TUR	-0,0007	-0,0008	-0,0060
PI_179722-IND(SG74)	0,0158	0,0313	0,0324
PI_180329-IND(SG50)	-0,1351	-0,0154	-0,1372
PI_180693-GER	-0,0158	-0,0160	-0,0118
PI_180699-GER(SG73)	0,0348	0,0109	-0,0140
PI_180702-GER	0,0023	-0,0140	-0,0162
PI_181799-LBN	0,0319	0,0071	-0,0119
PI_181801-LBN	0,0432	0,0190	-0,0129
PI_181958-SYR	0,0179	0,0020	-0,0181
PI_183467-IND	-0,0323	-0,1782	0,0723
PI_184130-HRV	0,0035	-0,0061	-0,0121
PI_184784-GIN	0,0358	0,0147	-0,0271
PI_193578-ETH	0,0244	0,0287	0,0200
PI_193584-ETH	0,0265	0,0288	0,0155
PI_193590-ETH	0,0226	0,0241	0,0167
PI_195020-ETH	0,0318	0,0082	-0,0210
PI_195404-GTM(SG72)	0,0276	0,0226	0,0027
PI_197990-NLD	0,0217	0,0146	-0,0048
PI_198072-SWE	0,0038	-0,0072	-0,0166
PI_198074-SWE	0,0043	-0,0451	0,0026
PI_198735-AFG	-0,0296	0,0588	0,1133
PI_201390-MEX	0,0231	0,0024	-0,0190
PI_203066-FIN(SG71)	-0,0014	-0,0126	-0,0224
PI_203067-FIN	0,0237	0,0015	-0,0140
PI_203068-FIN	0,0217	-0,0035	-0,0163
PI_203069-FIN(SG70)	0,0016	-0,0162	-0,0201
PI_204306-AUS	-0,0135	-0,0236	-0,0167
PI_206006-SWE(SG59)	0,0128	-0,0040	-0,0164
PI_206838-USA	0,0290	0,0105	-0,0115
PI_206861-USA	0,0383	0,0154	-0,0123
PI_207508-AFG	-0,0516	0,0691	0,1587
PI_209507-CRI	0,0267	0,0086	-0,0118
PI_210558-CHN	0,0188	0,0278	0,0220
PI_210561-RUS	0,0354	0,0156	-0,0125
PI_210569-FIN	0,0113	0,0111	0,0109
PI_210583-USA	0,0383	0,0166	-0,0151
PI_212031-IRN(SG4)	-0,0556	0,0730	0,1683
PI_212917-IND	0,0293	0,0173	-0,0031
PI_220189-AFG	-0,0490	0,0574	0,1289
PI_221697-IDN	-0,0040	-0,0237	-0,0155
PI_222117-AFG	-0,0544	0,0765	0,1660
PI_227258-IRN	-0,0408	0,0275	0,0592
PI_236492-USA	-0,0255	-0,0071	-0,0026
PI_241593-TWN	0,0343	0,0178	-0,0079
PI_242027-DNK(SG9)	-0,0821	-0,0180	-0,0768
PI_242028-DNK	0,0150	0,0017	-0,0105
PI_244093-NLD	0,0253	0,0190	0,0001
PI_244175-NLD	0,0333	0,0133	-0,0137
PI_244191-NLD	0,0365	0,0131	-0,0147
PI_248181-RWA	0,0059	-0,0202	-0,0087
PI_249645-IND	0,0251	0,0281	0,0156
PI_250439-CSK(SG32)	0,0335	0,0135	-0,0100
PI_250440-CSK(SG26)	0,0301	0,0103	-0,0188
PI_250441-CSK	0,0380	0,0206	-0,0101
PI_250444-CSK(SG6)	0,0448	0,0250	-0,0159
PI_250446-CSK(SG69)	0,0431	0,0207	-0,0199
PI_253968-AFG	-0,0495	0,0634	0,1466
PI_257592-ETH	0,0280	0,0102	-0,0069
PI_261622-SPA(SG28)	0,0475	0,0212	-0,0141
PI_261623-SPA	0,0353	0,0098	-0,0166
PI_261624-SPA(SG62)	0,0409	0,0216	-0,0122
PI_261671-NLD(SG66)	0,0149	0,0089	-0,0250
PI_261677-NLD	-0,0401	-0,0234	-0,0259
PI_263014-NLD(SG68)	0,0334	0,0147	-0,0176
PI_263030-FRA(SG67)	0,0339	0,0132	-0,0165
PI_263032-FRA(SG30)	0,0368	0,0151	-0,0180
PI_266070-SWE	0,0160	-0,0082	-0,0194
PI_269761-CSK	-0,0280	-0,0285	-0,0114
PI_269782-UK	0,0384	0,0154	-0,0150
PI_269791-UK	0,0273	0,0060	-0,0168
PI_269798-UK	0,0258	0,0064	-0,0155
PI_269804-UK	0,0259	0,0099	-0,0203
PI_269812-UK	-0,0072	-0,0479	0,0023
PI_269818-UK	-0,0201	0,0401	0,0669
PI_269825-UK	0,0280	0,0075	-0,0112
PI_270536-DNK	0,0071	-0,0076	-0,0238
PI_271116-CHN(SG64)	-0,0024	-0,0055	-0,0256
PI_272148-FIN	0,0059	-0,0132	-0,0119
PI_272171-GER	-0,0083	-0,0281	-0,0210
PI_272175-GER	0,0105	0,0147	0,0170
PI_272184-GRC	-0,0317	-0,1184	0,0337
PI_272194-GER	0,0229	0,0086	-0,0018
PI_272215-GER	-0,0091	-0,0872	0,0278
PI_272216-BGR	-0,0026	-0,0607	0,0187
PI_272218-POL	-0,0200	-0,0777	0,0127
PI_273209-RUS	-0,0777	0,0402	-0,0461
PI_273605-ECU	0,0259	0,0031	-0,0209
PI_274307-PAK(SG63)	-0,0521	0,0656	0,1419
PI_275821-SWE	0,0180	0,0041	-0,0177
PI_275825-SWE	0,0338	0,0160	-0,0118
PI_277852-ETH	0,0179	0,0112	-0,0013
PI_279825-GER	0,0368	0,0160	-0,0120
PI_280252-ETH	0,0292	0,0122	-0,0204
PI_280603-ISR	0,0337	0,0162	-0,0128
PI_280609-RUS	-0,0937	0,0094	0,0256
PI_280613-RUS	0,0268	0,0095	-0,0070
PI_280614-RUS	0,0414	0,0217	-0,0126
PI_280616-BLR	0,0099	-0,0068	-0,0222
PI_280617-EST	0,0258	0,0092	-0,0119
PI_280619-EST	0,0061	-0,0072	-0,0232
PI_280626-RUS	0,0385	0,0168	-0,0128
PI_285710-POL	0,0143	0,0048	-0,0097
PI_285715-POL	0,0087	-0,0131	-0,0148
PI_285717-POL	-0,0147	-0,0197	-0,0210
PI_285718-POL	0,0142	-0,0019	-0,0220
PI_285722-POL	0,0044	-0,0123	-0,0281
PI_285740-POL(SG61)	0,0405	0,0195	-0,0135
PI_286430-NPL	0,0052	0,0453	0,0566
PI_286607-THA(SG60)	0,0365	0,0217	-0,0006
PI_288025-FRA	0,0327	0,0088	-0,0158
PI_293426-BGR	-0,0323	-0,1733	0,0696
PI_306591-HUN	-0,0203	-0,0212	-0,0293
PI_307666-CRI	0,0269	0,0078	-0,0167
PI_308796-IND	0,0331	0,0118	-0,0142
PI_314794-AUS(SG11)	0,0307	0,0066	-0,0108
PI_314795-AUS	0,0382	0,0171	-0,0119
PI_324695-HUN	0,0296	0,0158	-0,0029
PI_324697-HUN	-0,0076	-0,0351	-0,0155
PI_324700-HUN	-0,0224	-0,1434	0,0622
PI_324702-HUN	-0,0273	-0,1626	0,0672
PI_324703-HUN	-0,0185	-0,0333	-0,0205
PI_331413-ETH	0,0143	0,0179	0,0054
PI_331414-ETH	0,0295	0,0111	-0,0194
PI_340128-TUR	-0,0324	-0,1473	0,0624
PI_340130-TUR	-0,0590	-0,0042	0,0083
PI_343292-USA	-0,0264	-0,1676	0,0683
PI_343321-USA	0,0274	0,0075	-0,0161
PI_343331-USA	0,0109	-0,0081	-0,0199
PI_343338-USA	0,0402	0,0129	-0,0195
PI_343958-TUR(SG58)	0,0314	0,0106	-0,0202
PI_343972-TUR	-0,1700	0,0748	-0,0787
PI_343977-TUR	-0,0068	0,0393	0,0603
PI_343979-TUR	-0,1414	0,0473	-0,0482
PI_343987-TUR	0,0085	-0,0118	-0,0108
PI_344003-TUR	0,0240	0,0022	-0,0252
PI_344007-GRC	-0,1626	0,0844	-0,0801
PI_344009-GRC	-0,0868	0,0464	-0,0527
PI_344011-GRC	-0,1588	0,0821	-0,0764
PI_344013-GRC(SG57)	-0,1629	0,0856	-0,0777
PI_344538-ITA	-0,1444	0,0699	-0,0700
PI_347295-IND	-0,0565	0,0389	0,0935
PI_347457-IND(SG56)	0,0206	0,0131	0,0059
PI_347490-IND	0,0371	0,0193	-0,0087
PI_347496-IND	0,0225	0,0150	0,0077
PI_356974-IND	0,0355	0,0199	-0,0074
PI_356980-IND	0,0242	0,0114	0,0013
PI_356984-IND	-0,0129	0,0476	0,0753
PI_356986-IND	0,0242	0,0134	0,0030
PI_356991-IND(SG22)	0,0203	0,0112	0,0042
PI_357290-MKD(SG54)	-0,0270	-0,1632	0,0660
PI_358300-ETH	0,0215	0,0125	-0,0156
PI_358633-ETH	0,0257	0,0189	0,0055
PI_365419-CAN	0,0379	0,0208	-0,0136
PI_371796-NZL	0,0396	0,0182	-0,0112
PI_378157-MYS	0,0326	0,0099	-0,0127
PI_393489-CSK	0,0390	0,0189	-0,0170
PI_393490-CSK	-0,0159	-0,1229	0,0503
PI_404225-BLR	-0,0303	-0,0392	-0,0226
PI_409031-GER	0,0055	-0,0145	-0,0262
PI_411141-NZL(SG29)	0,0419	0,0179	-0,0097
PI_413678-HUN(SG5)	0,0342	0,0088	-0,0144
PI_413685-HUN	0,0295	0,0159	-0,0132
PI_413703-HUN	0,0410	0,0208	-0,0189
PI_429839-AFG	-0,0119	0,0274	0,0379
PI_429843-LVA	0,0073	-0,0153	-0,0128
PI_429845-RUS	0,0153	-0,0108	-0,0061
PI_429849-UZB	-0,0293	-0,1743	0,0688
PI_430702-HUN	0,0437	0,0248	-0,0168
PI_476410-UKR(SG51)	0,0394	0,0221	-0,0122
PI_476413-RUS	0,0247	0,0021	-0,0108
PI_477371-DNK	0,0063	-0,0121	-0,0148
PI_486131-ECU	0,0233	0,0090	-0,0092
PI_494077-CHL	0,0312	0,0101	-0,0163
PI_499982-CHN	-0,1475	0,0698	-0,0695
PI_505062-GRC	-0,0202	-0,0859	0,0325
PI_505080-CYP(SG21)	0,0267	0,0042	-0,0096
PI_505108-GRC	-0,0290	-0,0947	0,0521
PI_505122-ALB	-0,0339	-0,1368	0,0443
PI_505127-ALB	-0,0116	-0,0052	-0,0252
PI_505144-SPA	0,0038	-0,0152	-0,0225
PI_508092-USA	0,0359	0,0189	-0,0133
PI_560055-RUS	-0,0560	0,0259	-0,0400
PI_560056-RUS	-0,0197	0,0261	-0,0284
PI_560058-ISR	-0,1542	0,0701	-0,0649
PI_560069-ISR	-0,1464	0,0736	-0,0621
PI_601426-USA	0,0385	0,0194	-0,0110
PI_601516-NLD	0,0411	0,0165	-0,0183
PI_614141-USA	0,0445	0,0203	-0,0116
PI_619079-USA	0,0344	0,0142	-0,0184
PI_639957-TUR	-0,1290	0,0562	-0,0342
PI_639959-TUR	-0,1175	0,0376	-0,0311
PI_639964-BGR	-0,1579	0,0517	-0,0382
PI_639967-IND	-0,0486	0,0696	0,1472
PI_639968-NPL	-0,0610	0,0794	0,1553
PI_639969-NPL	-0,0411	0,0743	0,1319
PI_639974-GEO	-0,0179	0,0139	-0,0243
PI_639977-BGR	-0,0226	-0,0886	0,0155
PI_639980-BGR(SG47)	-0,0053	-0,0851	0,0211
PI_639981-BGR	-0,0247	-0,1462	0,0526
Picar-FRA	-0,0328	-0,1733	0,0759
PIS_278-GER	0,0321	0,0086	-0,0217
PIS_2856-GER	0,0104	-0,0018	-0,0097
PIS_657-GER	-0,0055	-0,0220	-0,0239
PIS_845-GER(SG78)	0,0231	-0,0006	-0,0168
Piver-FRA	-0,0142	-0,1059	0,0249
PS0010128-USA	0,0388	0,0164	-0,0173
PS02101137-USA	0,0351	0,0163	-0,0179
PS03101445-USA	0,0332	0,0171	-0,0098
PS03101822-USA	0,0387	0,0169	-0,0160
PS04100710-USA	0,0348	0,0134	-0,0161
PS05100522-USA	0,0382	0,0149	-0,0125
PS05100632-USA	0,0369	0,0137	-0,0155
PS05100736-USA(SG19)	0,0377	0,0125	-0,0134
PS05100840-USA	0,0364	0,0062	-0,0114
PS05101142-USA	0,0276	0,0083	-0,0135
PS05101240-USA	0,0262	0,0225	0,0029
PS06100490-USA	0,0385	0,0140	-0,0136
PS06100542-USA	0,0355	0,0160	-0,0134
PS06100617-USA	0,0424	0,0168	-0,0153
PS06100760-USA	0,0372	0,0140	-0,0169
PS06101043-USA(SG46)	0,0376	0,0170	-0,0144
PS06101119-USA	0,0217	-0,0010	-0,0249
PS06310024W-USA	0,0068	-0,0365	0,0102
PS07100170-USA(SG45)	0,0391	0,0132	-0,0159
PS07100396-USA	0,0338	0,0119	-0,0132
PS07100471-USA	0,0000	0,0224	-0,0227
PS07100474-USA	0,0326	0,0028	-0,0125
PS07100480-USA(SG44)	0,0335	0,0057	-0,0179
PS07100914-USA	0,0339	0,0148	-0,0118
Pulawska_Slodka_Nr_2-POL	0,0053	-0,0067	-0,0166
Quarante-deux_de_Sarcelles-FRA	0,0251	0,0090	-0,0169
Raina_Victoria-UK	0,0107	-0,0122	-0,0202
S1047-USA	0,0355	0,0213	-0,0101
S1195-USA(SG1)	0,0410	0,0206	-0,0105
S1430-USA(SG42)	0,0351	0,0191	-0,0104
S1431-USA	0,0349	0,0186	-0,0074
S1456-USA	0,0398	0,0211	-0,0120
S1544-USA	0,0338	0,0178	-0,0090
S1558-USA(SG10)	0,0363	0,0208	-0,0046
S1573-USA	0,0391	0,0184	-0,0102
S1586-USA	0,0354	0,0197	-0,0085
S1587-USA	0,0231	0,0199	-0,0085
S1591-USA(SG16)	0,0363	0,0190	-0,0080
S875_1-USA	0,0352	0,0204	-0,0053
S906-USA	0,0364	0,0188	-0,0101
S947-USA(SG43)	0,0395	0,0189	-0,0106
Santana-GER	0,0312	0,0075	-0,0163
Serpette_D'Auvergne-FRA	0,0417	0,0192	-0,0219
Serpette_de_Paris-FRA	0,0423	0,0196	-0,0169
Serpette_de_Vitry-FRA	0,0252	-0,0212	-0,0048
Spirale_(PSC0013)-FRA	0,0262	0,0076	-0,0140
Triomphe_de_Roissy-FRA	0,0410	0,0197	-0,0145
Viriato-SPA	0,0274	-0,0125	-0,0059
W6_10096-CSK(SG14)	0,0432	0,0149	-0,0184
W6_10925-BGR	-0,0826	0,0328	-0,0281
W6_12723-BGR	-0,0111	-0,0833	0,0253
W6_12738-NA	-0,0291	-0,1742	0,0744
W6_12739-BGR	0,0177	0,0049	-0,0122
W6_15008-ISR	-0,0100	-0,0004	-0,0201
W6_15009-GEO	-0,0551	0,0110	-0,0289
W6_15010-LIT	-0,0740	0,0183	-0,0291
W6_15019-TUR(SG40)	-0,1425	-0,0249	-0,1403
W6_15028-NA	-0,0816	-0,0037	0,0153
W6_15043-NA(SG41)	-0,0036	-0,0242	-0,0223
W6_15048-NA	0,0222	0,0033	-0,0199
W6_15163-USA	0,0344	0,0121	-0,0108
W6_17293-PAK	-0,0628	0,0788	0,1476
W6_20025-BGR	-0,1529	0,0515	-0,0387
W6_20026-BGR	-0,1582	0,0580	-0,0389
W6_24570-TKM(SG39)	-0,1440	0,0514	-0,0445
W6_26127-GEO(SG38)	-0,1512	0,0683	-0,0523
W6_26154-GEO(SG37)	0,0325	0,0090	-0,0232
W6_26161-GEO(SG36)	0,0313	0,0098	-0,0213
W6_31707-RUS	0,0298	0,0127	-0,0104
W6_34960-UK	0,0136	0,0124	-0,0015
W6_39729-USA(SG35)	0,0417	0,0203	-0,0100
W6_44574-CHN	-0,0157	0,0442	0,0770
W6_44578-CHN(SG34)	0,0204	0,0246	0,0141
W6_44581-CHN	0,0112	0,0241	0,0282
W6_44583-CHN	0,0015	0,0326	0,0463
W6_44642-CHN	-0,0316	0,0566	0,0962
W6_44712-CHN	-0,0004	0,0354	0,0457
W6_44713-CHN	-0,0034	0,0380	0,0597
W6_44717-CHN	-0,0389	0,0236	0,0465
W6_44718-CHN	-0,0018	0,0369	0,0469
W6_44723-CHN	0,0048	0,0301	0,0429
W6_44724-CHN	0,0052	0,0388	0,0550
W6_44726-CHN	-0,0322	0,0588	0,1113
W6_44766-CHN	0,0358	0,0158	-0,0112
W6_44768-CHN	0,0081	0,0209	0,0260
W6_44769-CHN	0,0228	0,0128	-0,0053
W6_44770-CHN(SG33)	0,0115	0,0232	0,0278
ZP0076-SPA	0,0221	-0,0021	-0,0247
ZP0126-SPA	0,0094	-0,0108	-0,0242
ZP0181-SPA	0,0319	0,0061	-0,0187
ZP0202-SPA	0,0476	0,0225	-0,0153
ZP0213-SPA	-0,0102	-0,0269	-0,0176
ZP0535-SPA	-0,0169	-0,0352	-0,0272
ZP0798-SPA	0,0042	-0,0163	-0,0226
ZP0799-SPA	0,0059	-0,0093	-0,0146
ZP1261-SPA	0,0094	-0,0113	-0,0203
ZP1264-SPA	0,0242	0,0003	-0,0175
ZP1282-SPA(SG13)	0,0437	0,0212	-0,0133
ZP1294-SPA	-0,0059	-0,0579	-0,0024

Table S6. Genomic windows associated with putative domestication sweeps, sorted by decreasing XP-CLR score. Names and putative functions of the genes included in each window are reported

Window coordinates	XP-CLR score	Gene name and function
1LG6_288600001_289600000	40,39547224	Psat1g145040: Unknown gene
		Psat1g145080: Unknown gene
		Psat1g145120: Unknown gene
		Psat1g145160: Unknown gene
		Psat1g145240: Unknown gene
		Psat1g145280: Unknown gene
		Psat1g145320: Unknown gene
		Psat1g145360: Unknown gene
		Psat1g145400: Unknown gene
		Psat1g145520: Pyridoxal phosphate-dependent transferase + major region + subdomain 1
		Psat1g145560: Transferase activity + transferring phosphorus-containing groups
		Psat1g145200: Unknown gene
		Psat1g145440: Rab-GTPase-TBC domain
		Psat1g145480: Plasma membrane
5LG3_37000001_38000000	33,21736744	Psat5g019640: Unknown gene
		Psat5g019680: Unknown gene
		Psat5g019720: Unknown gene
		Psat5g019760: Erg28 like protein
		Psat5g019800: Unknown gene
		Psat5g019880: KH domain
		Psat5g019920: Epidermal patterning factor proteins
		Psat5g019960: Protein of unknown function (DUF1005)
		Psat5g020000: Poly(ADP-ribose) polymerase catalytic domain
		Psat5g020040: Protein of unknown function DUF597
		Psat5g020080: Putative serine esterase (DUF676)
		Psat5g020120: Unknown gene
		Psat5g019840: Transmembrane amino acid transporter protein
		Psat5g020160: Unknown gene
3LG5_157600001_158800000	29,89527166	Psat3g075960: N-acetylglucosaminyl transferase component (Gpi1)
		Psat3g076000: Unknown gene
		Psat3g076040: Cellular macromolecular complex subunit organization
		Psat3g076080: Unknown gene
		Psat3g076120: Gibberellin regulated protein
		Psat3g076160: Reverse transcriptase (RNA-dependent DNA polymerase)
		Psat3g076200: Unknown gene
		Psat3g076240: Unknown gene
		Psat3g076280: Unknown gene
		Psat3g076320: Protein phosphatase 2C
		Psat3g076360: NAD dependent epimerase/dehydratase family
		Psat3g076520: Unknown gene
		Psat3g076560: Helix-loop-helix DNA-binding domain
		Psat3g076640: Unknown gene
		Psat3g076680: Helix-loop-helix DNA-binding domain
		Psat3g076720: Unknown gene
		Psat3g076760: Transition metal ion binding
		Psat3g076800: Polynucleotide kinase 3 phosphatase
		Psat3g076840: Poly(ADP-ribose) polymerase and DNA-Ligase Zn-finger region
		Psat3g076880: Unknown gene
		Psat3g076920: Poly(ADP-ribose) polymerase and DNA-Ligase Zn-finger region
		Psat3g076960: Poly(ADP-ribose) polymerase and DNA-Ligase Zn-finger region
		Psat3g077000: Rab3 GTPase-activating protein regulatory subunit N-terminus
		Psat3g077040: Poly(ADP-ribose) polymerase and DNA-Ligase Zn-finger region
		Psat3g077080: EF-hand domain pair
		Psat3g076400: Plant calmodulin-binding domain
		Psat3g076440: Domain of unknown function (DUF3336)
		Psat3g076480: Helix-loop-helix DNA-binding domain
3LG5_255800001_257600000	24,94894578	Psat3g130920: Unknown gene
		Psat3g130960: Protein of unknown function (DUF740)
		Psat3g131000: Zinc finger C2H2 type domain signature.
		Psat3g131080: Transmembrane amino acid transporter protein
		Psat3g131120: Intracellular membrane-bounded organelle
		Psat3g131160: PPR repeat family
		Psat3g131040: Haloacid dehalogenase-like hydrolase
4LG4_38800001_40600000	23,40165515	Psat4g027280: Unknown gene
		Psat4g027320: Ulp1 protease family + C-terminal catalytic domain
		Psat4g027360: BTB/POZ domain
		Psat4g027400: Unknown gene
		Psat4g027440: Unknown gene
		Psat4g027480: AIG1 family
		Psat4g027520: SAM-dependent methyltransferase PRMT-type domain profile.
		Psat4g027560: Eukaryotic porin
		Psat4g027600: SANT domain profile.
		Psat4g027640: Galactose oxidase + central domain
7LG7_330600001_331600000	23,2405493	Psat7g174160: RNA recognition motif. (a.k.a. RRM + RBD + or RNP domain)
		Psat7g174240: Domain of unknown function (DUF3067)
		Psat7g174280: Dirigent-like protein
		Psat7g174320: Dirigent-like protein
		Psat7g174360: Amidase
		Psat7g174480: Magnesium transporter NIPA
		Psat7g174560: Unknown gene
		Psat7g174600: Protein of unknown function (DUF3755)
		Psat7g174720: Unknown gene
		Psat7g174760: PPR repeat family
		Psat7g174800: Phosphatidylinositol-specific phospholipase C + X domain
		Psat7g174880: Unknown gene
		Psat7g174920: NB-ARC domain
		Psat7g174960: NB-ARC domain
		Psat7g175000: Armadillo-like helical
		Psat7g175040: Armadillo/beta-catenin-like repeat
		Psat7g175160: Purine ribonucleotide binding
		Psat7g175200: Von Willebrand factor type A domain
		Psat7g175280: Unknown gene
		Psat7g174200: Unknown gene
		Psat7g174440: Plant specific eukaryotic initiation factor 4B
		Psat7g174520: Protein kinase domain
		Psat7g174680: 2OG-Fe(II) oxygenase superfamily
		Psat7g174840: NB-ARC domain
		Psat7g175080: ATP synthase (E/31 kDa) subunit
		Psat7g175240: Organic solute transporter Ostalpha
		Psat7g174400: Amidase
6LG2_362600001_363600000	22,19154914	Psat6g180520: Unknown gene
		Psat6g180600: Proline rich extensin signature
		Psat6g180640: Papain family cysteine protease
		Psat6g180680: Unknown gene
		Psat6g180720: Unknown gene
		Psat6g180760: Cyclophilin type peptidyl-prolyl cis-trans isomerase/CLD
		Psat6g180800: Unknown gene
		Psat6g180840: Unknown gene
		Psat6g180880: Unknown gene
		Psat6g180920: Transferase activity + transferring phosphorus-containing groups
		Psat6g180960: Myc-type + basic helix-loop-helix (bHLH) domain profile.
		Psat6g181000: AP endonucleases family 1 signature 1.
		Psat6g181040: Unknown gene
		Psat6g181120: Domain of unknown function (DUF3783)
		Psat6g181080: CLASP N terminal
5LG3_566600001_567800000	21,55277216	Psat5g299400: AUX/IAA family
		Psat5g299440: Intracellular membrane-bounded organelle
		Psat5g299480: Transferase activity + transferring phosphorus-containing groups
		Parent=Psat5g299520: Transferase activity + transferring phosphorus-containing groups
		Psat5g299560: Cyclin + N-terminal domain
		Psat5g299600: Protein kinase domain
		Psat5g299640: Ribosomal L27e protein family
		Psat5g299680: ETC complex I subunit conserved region
		Psat5g299720: 2OG-Fe(II) oxygenase superfamily
		Psat5g299760: Unknown gene
2LG1_68800001_69800000	17,33181862	Psat2g041440: NAC domain
		Psat2g041480: RNA recognition motif. (a.k.a. RRM + RBD + or RNP domain)
		Psat2g041520: Unknown gene
		Psat2g041560: C2 domain
		Psat2g041600: RNA recognition motif. (a.k.a. RRM + RBD + or RNP domain)
		Psat2g041640: Radical SAM superfamily
		Psat2g041400: RNA recognition motif. (a.k.a. RRM + RBD + or RNP domain)
		Psat2g041680: Protein kinase domain
		Psat2g041680: Protein tyrosine kinase
4LG4_189400001_190800000	15,16253804	Psat4g100520: Unknown gene
		Psat4g100600: Unknown gene
		Psat4g100680: Unknown gene
		Psat4g100720: 16S rRNA methyltransferase RsmB/F
		Psat4g100800: Rhodanese-like domain
		Psat4g100840: Unknown gene
		Psat4g100880: Unknown gene
		Psat4g100960: Senescence-associated protein
		Psat4g101000: Unknown gene
		Psat4g101040: Allergen V5/Tpx-1 related
		Psat4g101080: Allergen V5/Tpx-1 related
		Psat4g101120: Allergen V5/Tpx-1 related
		Psat4g101160: Unknown gene
		Psat4g101200: Hydrolase activity + acting on acid anhydrides + in phosphorus-containing anhydrides
		Psat4g100640: Lysine methyltransferase
		Psat4g100920: Von Willebrand factor type A domain
		Psat4g101240: MCM2/3/5 family
3LG5_292000001_293000000	13,17989079	Psat3g151080: Unknown gene
		Psat3g151160: Unknown gene
		Psat3g151200: Unknown gene
		Psat3g151280: Transferase activity + transferring acyl groups other than amino-acyl groups
		Psat3g151320: Unknown gene
		Psat3g151360: MazG-like family
		Psat3g151400: Myb-like DNA-binding domain
		Psat3g151600: Serine carboxypeptidase
		Psat3g151640: CAF1 family ribonuclease
		Psat3g151680: Unknown gene
		Psat3g151760: Intracellular membrane-bounded organelle
		Psat3g151800: Unknown gene
		Psat3g151120: Plasma membrane
		Psat3g151240: SOS response associated peptidase (SRAP)
		Psat3g151720: FAD dependent oxidoreductase
		Psat3g151560: Serine carboxypeptidase
5LG3_520000001_521200000	13,04482658	Psat5g264160: Unknown gene
		Psat5g264200: Receptor family ligand binding region
		Psat5g264240: Unknown gene
		Psat5g264360: Unknown gene
		Psat5g264400: Unknown gene
		Psat5g264440: Unknown gene
		Psat5g264480: Unknown gene
		Psat5g264560: Unknown gene
		Psat5g264600: Late embryogenesis abundant protein
		Psat5g264680: Late embryogenesis abundant protein
		Psat5g264120: Receptor family ligand binding region
		Psat5g264320: START domain
		Psat5g264520: RING-H2 zinc finger domain
		Psat5g264640: Unknown gene
7LG7_191600001_192600000	12,65377242	Psat7g116840: Unknown gene
		Psat7g116920: GDSL-like Lipase/Acylhydrolase
		Psat7g116960: Unknown gene
		Psat7g117000: AP2 domain
		Psat7g117040: Ras family
		Psat7g117080: Unknown gene
		Psat7g117120: Unknown gene
		Psat7g117160: Homeobox domain
		Psat7g117200: Ras family
		Psat7g117280: Transcription elongation factor Elf1 like
		Psat7g117320: Tetratricopeptide repeat
		Psat7g117360: Cytochrome P450
		Psat7g116800: Protein of unknown function (DUF2775)
		Psat7g117240: Casein kinase substrate phosphoprotein PP28
3LG5_270600001_272400000	12,5656262	Psat3g139200: Unknown gene
		Psat3g139240: Hydrolase activity + acting on acid anhydrides + catalyzing transmembrane movement of substances
		Psat3g139280: WRKY DNA -binding domain
		Psat3g139360: Unknown gene
		Psat3g139400: Clathrin adaptor + mu subunit + C-terminal
		Psat3g139480: Unknown gene
		Psat3g139520: Myb-like DNA-binding domain
		Psat3g139600: Unknown gene
		Psat3g139640: Transferase activity + transferring phosphorus-containing groups
		Psat3g139680: Protein kinase domain
		Psat3g139800: Cytochrome P450 + E-class + group I
		Psat3g139840: Expansin signature
		Psat3g139880: Unknown gene
		Psat3g139320: Unknown gene
		Psat3g139560: Tub family
		Psat3g139760: Polycomb protein + VEFS-Box
1LG6_39400001_40400000	12,11028642	Psat1g027600: Tetraspanin family
		Psat1g027640: AMP-binding enzyme
		Psat1g027680: Unknown gene
		Psat1g027720: Phospholipid-translocating P-type ATPase C-terminal
		Psat1g027760: DnaJ domain
		Psat1g027800: Unknown gene
		Psat1g027840: Unknown gene
		Psat1g027880: Peptidase S24-like
		Psat1g027920: Peptidase S24-like
		Psat1g027960: Transcription regulator activity
		Psat1g028000: Unknown gene
		Psat1g028040: Enolase + C-terminal TIM barrel domain
		Psat1g028080: Protein of unknown function (DUF1637)
		Psat1g028120: Unknown gene
3LG5_407600001_408800000	11,40437962	Psat3g190960: TCP family transcription factor
		Psat3g191000: Purine ribonucleotide binding
		Psat3g191040: ABC transporter
		Psat3g191080: Unknown gene
		Psat3g191120: ABC transporter
		Psat3g191160: Unknown gene
		Psat3g191200: Unknown gene
		Psat3g191240: Unknown gene
		Psat3g191320: Initiation factor 2 subunit family
		Psat3g191440: Nuclear transport factor 2 (NTF2) domain
		Psat3g191480: NB-ARC domain
		Psat3g191600: Hydrolase activity + acting on acid anhydrides + in phosphorus-containing anhydrides
		Psat3g191280: Down-regulated-in-metastasis protein
		Psat3g191400: Protein tyrosine kinase
		Psat3g191560: Unknown gene
		Psat3g191640: Helicase associated domain (HA2)
6LG2_315600001_317000000	11,3662689	Psat6g158720: AUX/IAA family
		Psat6g158760: Unknown gene
		Psat6g158800: DHHC palmitoyltransferase
		Psat6g158880: Glycosyl hydrolases family 28
		Psat6g158960: Unknown gene
		Psat6g159040: ABC transporter transmembrane region
		Psat6g159080: Unknown gene
		Psat6g159120: HTH transcriptional regulator + Myb-type + DNA-binding
		Psat6g158840: Protein of unknown function (DUF861)
		Psat6g158920: SecA DEAD-like domain
		Psat6g159000: Aminotransferase class-V
1LG6_25000001_26000000	10,29662814	Psat1g017440: NAF domain
		Psat1g017520: Mitochondrial branched-chain alpha-ketoacid dehydrogenase kinase
		Psat1g017560: Unknown gene
		Psat1g017600: Unknown gene
		Psat1g017720: Proteasome assembly chaperone 4
		Psat1g017800: RNA recognition motif. (a.k.a. RRM + RBD + or RNP domain)
		Psat1g017840: Unknown gene
		Psat1g017880: Protein of unknown function + DUF538
		Psat1g017920: Ubiquitin family
		Psat1g018000: Unknown gene
		Psat1g018040: S-adenosylmethionine synthetase + C-terminal domain
		Psat1g018080: Late nodulin protein
		Psat1g018120: SBP domain
		Psat1g018160: AWPM-19-like family
		Psat1g018200: Unknown gene
		Psat1g017680: Sec20
		Psat1g017760: Protein kinase domain
		Psat1g017960: Unknown gene
		Psat1g017480: Protein of unknown function (DUF 659)
2LG1_255200001_257000000	10,04361571	Psat2g099440: Peptidase inhibitor I9
		Psat2g099480: Lysine methyltransferase
		Psat2g099520: SNARE associated Golgi protein
		Psat2g099560: SNARE associated Golgi protein
		Psat2g099600: Unknown gene
		Psat2g099640: Transition metal ion binding
		Psat2g099680: Unknown gene
		Psat2g099720: Ribosomal protein S2 signature
		Psat2g099760: Unknown gene
		Psat2g099880: Myb-like DNA-binding domain
		Psat2g099920: Transferase activity + transferring phosphorus-containing groups
		Psat2g100000: Unknown gene
		Psat2g100040: Nuclear fragile X mental retardation-interacting protein 1 + conservedregion
		Psat2g100080: Endonuclease/Exonuclease/phosphatase family
		Psat2g100120: Unknown gene
		Psat2g099960: Protein of unknown function DUF3475
5LG3_30000001_31000000	9,862665852	Psat5g016000: Myc-type + basic helix-loop-helix (bHLH) domain profile.
		Psat5g016040: Unknown gene
		Psat5g016080: Unknown gene
		Psat5g016120: F-box associated
		Psat5g016160: Inositol-pentakisphosphate 2-kinase
		Psat5g016200: CRAL/TRIO domain
		Psat5g016320: Unknown gene
		Psat5g016360: Retroviral aspartyl protease
		Psat5g016400: Unknown gene
		Psat5g016440: Unknown gene
		Psat5g016240: Bifunctional nuclease
		Psat5g016280: Cytochrome P450
7LG7_229200001_231000000	9,492301172	Psat7g136320: Terpene synthase family + metal binding domain
		Psat7g136360: Terpene synthase family + metal binding domain
		Psat7g136400: Terpene synthase family + metal binding domain
		Psat7g136480: Unknown gene
		Psat7g136520: Protein modification by small protein conjugation or removal
		Psat7g136560: F-box-like
		Psat7g136640: Photosystem II oxygen evolving complex protein PsbP
		Psat7g136440: Formin Homology 2 Domain
		Psat7g136600: Protein tyrosine kinase
		Psat7g136600: Ethylene-responsive protein kinase Le-CTR1
2LG1_6600001_7600000	9,267293646	Psat2g007200: Protein of unknown function (DUF2775)
		Psat2g007240: Legume lectin domain
		Psat2g007320: Unknown gene
		Psat2g007360: Unknown gene
		Psat2g007440: Chloroplast import apparatus Tic20-like
		Psat2g007480: Histone-like transcription factor (CBF/NF-Y) and archaeal histone
		Psat2g007520: Unknown gene
		Psat2g007560: DEK C terminal domain
		Psat2g007600: Pentatricopeptide (PPR) repeat profile.
		Psat2g007640: PPR repeat family
		Psat2g007680: DYW family of nucleic acid deaminases
		Psat2g007720: Pyridoxal-phosphate dependent enzyme
		Psat2g007800: Ion channel
		Psat2g007840: Cell part
		Psat2g007880: Cell part
		Psat2g007920: Unknown gene
		Psat2g007960: Unknown gene
		Psat2g008000: Unknown gene
		Psat2g008040: Unknown gene
		Psat2g008080: NLI interacting factor-like phosphatase
		Psat2g008120: Unknown gene
		Psat2g008200: Pectinesterase
		Psat2g008240: Bacterial transferase hexapeptide (six repeats)
		Psat2g008320: Serine acetyltransferase + N-terminal
		Psat2g008400: Unknown gene
		Psat2g008440: PPR repeat family
		Psat2g007400: Unknown gene
		Psat2g008160: Isy1-like splicing family
		Psat2g008280: Serine acetyltransferase + N-terminal
		Psat2g008360: Unknown gene
		Psat2g007280: Cytidylyltransferase-like
5LG3_141200001_142800000	8,976115837	Psat5g078720: Short chain dehydrogenase
		Psat5g078760: UDP-glucose:Glycoprotein Glucosyltransferase
		Psat5g078800: Protein serine/threonine phosphatase activity
		Psat5g078840: Short-chain dehydrogenase/reductase SDR
		Psat5g078880: Protein phosphatase 2C
		Psat5g078920: Unknown gene
		Psat5g078960: Unknown gene
		Psat5g079000: Aldo-keto reductase signature
		Psat5g079040: GHMP kinases N terminal domain
		Psat5g079080: Protein of unknown function (DUF668)
		Psat5g079120: Unknown gene
		Psat5g079320: Unknown gene
		Psat5g079360: Polyketide cyclase / dehydrase and lipid transport
		Psat5g079440: Unknown gene
		Psat5g079480: Unknown gene
		Psat5g079520: Unknown gene
		Psat5g079560: Cellular nucleobase + nucleoside + nucleotide and nucleic acid metabolic process
		Psat5g079600: Sugar efflux transporter for intercellular exchange
		Psat5g079680: WRKY DNA -binding domain
		Psat5g079200: Domain of unknown function (DUF3444)
		Psat5g079240: Trehalose-phosphatase
		Psat5g079280: Transferase activity + transferring phosphorus-containing groups
		Psat5g079400: RNA recognition motif. (a.k.a. RRM + RBD + or RNP domain)
		Psat5g079640: Sigma-70 + region 4
5LG3_449600001_450600000	8,302640727	Psat5g224720: SH2 domain
		Psat5g224760: ABC transporter transmembrane region
		Psat5g224800: Intrinsic to membrane
		Psat5g224840: Unknown gene
		Psat5g224880: Intracellular membrane-bounded organelle
		Psat5g224920: Unknown gene
6LG2_361600001_362600000	7,835232611	Psat6g180160: Unknown gene
		Psat6g180240: Protein of unknown function (DUF4050)
		Psat6g180280: Unknown gene
		Psat6g180320: Unknown gene
		Psat6g180360: Unknown gene
		Psat6g180200: U-box domain
2LG1_145400001_146800000	7,758970933	Psat2g076800: Seed dormancy control
		Psat2g076840: Protein kinase domain
		Psat2g076920: Unknown gene
		Psat2g076960: Unknown gene
		Psat2g077000: Unknown gene
		Psat2g077080: B3 DNA binding domain
		Psat2g077160: Unknown gene
		Psat2g077240: Unknown gene
		Psat2g077280: Cytochrome P450 + E-class + group I
		Psat2g076880: Unknown gene
		Psat2g077040: Eukaryotic molybdopterin domain signature
		Psat2g077120: UDP-glucoronosyl and UDP-glucosyl transferase
		Psat2g077320: Unknown gene
2LG1_392400001_393600000	7,20675993	Psat2g160360: SNF2 family N-terminal domain
		Psat2g160400: Unknown gene
		Psat2g160440: Sodium/hydrogen exchanger family
		Psat2g160480: Sodium/hydrogen exchanger family
		Psat2g160520: Pterin 4 alpha carbinolamine dehydratase
		Psat2g160600: Protein tyrosine kinase
		Psat2g160640: SDA1
		Psat2g160720: Transcription initiation factor IIB signature
		Psat2g160760: Protein of unknown function (DUF1195)
		Psat2g160800: Sugar (and other) transporter
		Psat2g160880: Protein of unknown function (DUF620)
		Psat2g160920: PPR repeat family
		Psat2g161000: Unknown gene
		Psat2g160560: Sulfite exporter TauE/SafE
		Psat2g160680: Unknown gene
		Psat2g160960: Sin-like protein conserved region
3LG5_218200001_219200000	6,558304749	Psat3g112760: Unknown gene
		Psat3g112800: Unknown gene
		Psat3g112840: C2 domain
		Psat3g112880: Phospholipase D C terminal
		Psat3g112960: Unknown gene
		Psat3g113040: Unknown gene
		Psat3g113080: Unknown gene
		Psat3g113160: Unknown gene
		Psat3g113200: Plant specific mitochondrial import receptor subunit TOM20
		Psat3g113240: Armadillo/beta-catenin-like repeat
		Psat3g112920: Spinocerebellar ataxia type 10 protein domain
		Psat3g113120: Unknown gene
3LG5_199600001_200600000	6,4525826	Psat3g099120: Unknown gene
		Psat3g099200: Unknown gene
		Psat3g099240: Unknown gene
		Psat3g099280: Unknown gene
		Psat3g099320: Unknown gene
		Psat3g099360: Lipase (class 3)
		Psat3g099400: Unknown gene
		Psat3g099440: Unknown gene
		Psat3g099520: Unknown gene
		Psat3g099600: RNA recognition motif. (a.k.a. RRM + RBD + or RNP domain)
		Psat3g099640: Cyclophilin type peptidyl-prolyl cis-trans isomerase/CLD
		Psat3g099720: Myb/SANT-like DNA-binding domain
		Psat3g099760: Copine
		Psat3g099800: Cyclin
		Psat3g099560: DAHP synthetase I family
		Psat3g099160: Hyaluronan / mRNA binding family
		Psat3g099480: Unknown gene
7LG7_242000001_243400000	6,042033371	Psat7g140000: Unknown gene
		Psat7g140160: BZIP transcription factor
		Psat7g140200: SnoRNA binding domain + fibrillarin
		Psat7g140240: Unknown gene
		Psat7g140280: Unknown gene
		Psat7g140360: Arginine decarboxylase signature
		Psat7g140440: Auxin responsive protein
		Psat7g140480: Auxin responsive protein
		Psat7g140520: Auxin responsive protein
		Psat7g140560: Unknown gene
		Psat7g140600: Cytochrome P450
		Psat7g140680: Unknown gene
		Psat7g140640: RNA recognition motif. (a.k.a. RRM + RBD + or RNP domain)
1LG6_357800001_358800000	5,986924686	Psat1g207320: Bromodomain
		Psat1g207360: Unknown gene
		Psat1g207400: CBF/Mak21 family
		Psat1g207440: Unknown gene
		Psat1g207480: Protein of unknown function (DUF707)
		Psat1g207520: Transferase activity + transferring glycosyl groups
		Psat1g207560: Establishment of protein localization
		Psat1g207600: Establishment of protein localization
		Psat1g207640: Unknown gene
		Psat1g207680: Inositol monophosphatase family
		Psat1g207760: Transmembrane amino acid transporter protein
		Psat1g207800: S1/P1 Nuclease
		Psat1g207840: Ribosomal proteins L26 eukaryotic + L24P archaeal
		Psat1g207880: Zinc-binding dehydrogenase
		Psat1g207920: Zinc-binding dehydrogenase
		Psat1g207960: Organic solute transporter Ostalpha
		Psat1g208000: Cotton fibre expressed protein
		Psat1g208080: Unknown gene
		Psat1g208120: Unknown gene
		Psat1g208200: Syntaxin
		Psat1g208280: Ribosomal protein S2 signature
		Psat1g208360: Protein tyrosine kinase
		Psat1g208440: Unknown gene
		Psat1g208480: Unknown gene
		Psat1g208520: Unknown gene
		Psat1g208640: Unknown gene
		Psat1g208040: Tetratricopeptide repeat
		Psat1g208240: B3 DNA binding domain
		Psat1g208320: Diacylglycerol kinase catalytic domain
		Psat1g208400: Unknown gene
		Psat1g208560: Unknown gene
1LG6_33200001_34600000	5,913505501	Psat1g023720: Unknown gene
		Psat1g023760: Unknown gene
		Psat1g023800: Unknown gene
		Psat1g023840: Putative serine esterase (DUF676)
		Psat1g023880: Unknown gene
		Psat1g023960: SPOC domain
		Psat1g024000: Cellular nucleobase + nucleoside + nucleotide and nucleic acid metabolic process
		Psat1g024080: Unknown gene
		Psat1g024120: Unknown gene
		Psat1g024160: Ammonium Transporter Family
		Psat1g024200: Unknown gene
		Psat1g024240: Unknown gene
		Psat1g024320: Rad51
		Psat1g024360: Unknown gene
		Psat1g024400: Actin
		Psat1g024480: Myb-like DNA-binding domain
		Psat1g024520: Transferase family
		Psat1g024560: Unknown gene
		Psat1g024600: Acyl-ACP thioesterase
		Psat1g024640: Unknown gene
		Psat1g024720: Heme oxygenase
		Psat1g024760: Metalloenzyme superfamily
		Psat1g024800: F-box associated
		Psat1g024840: Glycosyl hydrolases family 17
		Psat1g024880: Stabilization of polarity axis
		Psat1g023920: Inositol monophosphatase family
		Psat1g024440: Unknown gene
		Psat1g024040: SPX domain
		Psat1g024680: Nuclear pore complex scaffold + nucleoporins 186/192/205
7LG7_37200001_38200000	5,910164678	Psat7g024160: SnoaL-like domain
		Psat7g024200: Unknown gene
		Psat7g024240: Acyltransferase C-terminus
		Psat7g024280: ATPase family associated with various cellular activities (AAA)
		Psat7g024320: Recoverin family signature
		Psat7g024360: Recoverin family signature
		Psat7g024400: Phosphatidylinositol 3- and 4-kinase
		Psat7g024440: Late embryogenesis abundant protein
		Psat7g024480: Unknown gene
		Psat7g024560: Plant transposase (Ptta/En/Spm family)
		Psat7g024640: Ring finger domain
		Psat7g024720: Development and cell death domain
		Psat7g024760: Unknown gene
		Psat7g024800: Glutaredoxin
		Psat7g024840: Unknown gene
		Psat7g024680: Ring finger domain
		Psat7g024600: Wall-associated receptor kinase galacturonan-binding
6LG2_430200001_431200000	5,83474017	Psat6g215880: Protein of unknown function (DUF620)
		Psat6g215960: Protein phosphatase 2C
		Psat6g216000: Ring finger domain
		Psat6g216040: Unknown gene
		Psat6g216080: 2OG-Fe(II) oxygenase superfamily
		Psat6g216120: Unknown gene
		Psat6g216160: Unknown gene
		Psat6g216240: Unknown gene
		Psat6g216360: Unknown gene
		Psat6g216400: Pumilio-family RNA binding repeat
		Psat6g216440: Unknown gene
		Psat6g216480: Thioredoxin
		Psat6g216640: Zinc finger C2H2 type domain profile.
		Psat6g216680: Unknown gene
		Psat6g216200: FAR1 DNA-binding domain
		Psat6g216280: PHD-finger
		Psat6g216560: Trp-Asp (WD) repeats profile.
		Psat6g216600: TIP41-like family
		Psat6g216320: Protein of unknown function (DUF760)
5LG3_553600001_554800000	5,125206632	Psat5g287640: Unknown gene
		Psat5g287680: Protein kinase domain
		Psat5g287720: Unknown gene
		Psat5g287760: Unknown gene
		Psat5g287800: Unknown gene
		Psat5g287840: Unknown gene
		Psat5g287920: Unknown gene
		Psat5g287960: Unknown gene
		Psat5g288120: Unknown gene
		Psat5g288160: Zinc knuckle
		Psat5g288200: MULE transposase domain
		Psat5g288240: Helix-loop-helix DNA-binding domain
		Psat5g288280: Unknown gene
		Psat5g288320: Unknown gene
		Psat5g288360: High mobility group + superfamily
		Psat5g288400: Unknown gene
		Psat5g288440: High mobility group + superfamily
		Psat5g287880: AP2 domain
		Psat5g288000: Nucleic acid binding protein NABP
		Psat5g288040: Unknown gene
		Psat5g288080: Leucine rich repeat N-terminal domain
1LG6_81600001_83200000	4,949727463	Psat1g052320: Actin
		Psat1g052400: Unknown gene
		Psat1g052440: Unknown gene
		Psat1g052480: Diacylglycerol kinase catalytic domain
		Psat1g052520: Proline rich extensin signature
		Psat1g052640: Secondary active transmembrane transporter activity
		Psat1g052680: MatE
		Psat1g052760: YbaK/aminoacyl-tRNA synthetase-associated domain
		Psat1g052800: Thiamine pyrophosphate enzyme + N-terminal TPP binding domain
		Psat1g052880: Unknown gene
		Psat1g052920: Unknown gene
		Psat1g052960: Protein tyrosine kinase
		Psat1g053000: MULE transposase domain
		Psat1g053120: Protein kinase domain
		Psat1g053160: Protein of unknown function (DUF3531)
		Psat1g053200: Unknown gene
		Psat1g053240: Pyruvate kinase + barrel domain
		Psat1g053320: Ferredoxin thioredoxin reductase variable alpha chain
		Psat1g053360: Unknown gene
		Psat1g053440: Purine ribonucleotide binding
		Psat1g053480: Mevalonate kinase family signature
		Psat1g053480: GHMP kinases N terminal domain
		Psat1g053520: Unknown gene
		Psat1g053560: Unknown gene
		Psat1g053640: Cytochrome P450
		Psat1g053720: Aldose 1-epimerase
		Psat1g053760: Uso1 / p115 like vesicle tethering protein + C terminal region
		Psat1g053800: CRS1 / YhbY (CRM) domain
		Psat1g053840: Cytochrome P450
		Psat1g052360: Unknown gene
		Psat1g052560: LIS1 homology (LisH) motif profile.
		Psat1g052840: Leucine-rich repeat
		Psat1g053040: Unknown gene
		Psat1g053080: Con80 domain of Katanin
		Psat1g053280: Trypsin-like peptidase domain
		Psat1g053400: TruB family pseudouridylate synthase (N terminal domain)
		Psat1g053600: Cytochrome P450
		Psat1g053680: Unknown gene
2LG1_101200001_102200000	4,8205225	Psat2g057480: Unknown gene
		Psat2g057520: Pyridine nucleotide-disulphide oxidoreductase
		Psat2g057560: Unknown gene
		Psat2g057600: Rdx family
		Psat2g057640: Jacalin-like lectin domain
		Psat2g057680: DYW family of nucleic acid deaminases
		Psat2g057760: Sugar (and other) transporter
		Psat2g057800: Unknown gene
		Psat2g057840: Unknown gene
		Psat2g057880: Unknown gene
		Psat2g057920: Unknown gene
		Psat2g057960: DUF761-associated sequence motif
		Psat2g058000: Ankyrin repeats (3 copies)
5LG3_571400001_572400000	4,554749086	Psat5g302600: Unknown gene
		Psat5g302640: Unknown gene
		Psat5g302720: CBS domain
		Psat5g302720: Unknown gene
		Psat5g302800: C-terminal to LisH (CTLH) motif profile.
		Psat5g302840: WD domain + G-beta repeat
		Psat5g302880: Unknown gene
		Psat5g302920: Unknown gene
		Psat5g302960: MIF4G domain
		Psat5g303000: Unknown gene
		Psat5g303040: Pectinesterase
		Psat5g303080: Thioredoxin-like [2Fe-2S] ferredoxin
		Psat5g303120: Formamidopyrimidine-DNA glycosylase H2TH domain
		Psat5g303160: HAT family C-terminal dimerisation region
		Psat5g302560: Protein kinase domain
		Psat5g302560: Protein tyrosine kinase
		Psat5g302680: Leucine rich repeat
2LG1_103600001_104600000	4,522281185	Psat2g059120: Unknown gene
		Psat2g059160: Myb-like DNA-binding domain
		Psat2g059200: Cytochrome P450
		Psat2g059240: Thylakoid soluble phosphoprotein TSP9
		Psat2g059280: Machado-Joseph disease protein MJD
		Psat2g059320: Josephin
		Psat2g059360: Unknown gene
		Psat2g059400: Unknown gene
		Psat2g059480: Myb-like DNA-binding domain
		Psat2g059520: Unknown gene
		Psat2g059640: POT family
		Psat2g059680: Major facilitator superfamily + general substrate transporter
		Psat2g059720: Thioesterase superfamily
		Psat2g059800: Major Facilitator Superfamily
		Psat2g059840: WD domain + G-beta repeat
		Psat2g059880: Unknown gene
		Psat2g059960: Protein of unknown function DUF125 + transmembrane
		Psat2g060000: Unknown gene
		Psat2g060040: WD40 repeat-like-containing domain
		Psat2g059080: Unknown gene
		Psat2g059560: Unknown gene
		Psat2g059600: Multicopper oxidase
		Psat2g059760: Retrotransposon gag protein
		Psat2g059440: Unknown gene
		Psat2g059920: Retroviral aspartyl protease
2LG1_77600001_78800000	4,37081148	Psat2g044200: AAA domain
		Psat2g044240: Unknown gene
		Psat2g044280: AAA domain
		Psat2g044360: RNA polymerase Rpb1 + domain 5
		Psat2g044400: Ribosomal protein S2
		Psat2g044440: Ribosomal protein S2
		Psat2g044480: Unknown gene
		Psat2g044520: Squalene/phytoene synthase
		Psat2g044560: Unknown gene
		Psat2g044600: Unknown gene
		Psat2g044640: Protein of unknown function (DUF775)
		Psat2g044680: PPR repeat family
		Psat2g044720: PRP38
		Psat2g044800: Transcription factor IIIC subunit delta N-term
		Psat2g044840: Universal stress protein family
		Psat2g044880: Unknown gene
		Psat2g044920: U-box domain
		Psat2g044960: Unknown gene
		Psat2g045000: Protein kinase domain
		Psat2g045040: Unknown gene
		Psat2g045080: Unknown gene
		Psat2g045120: Ribosomal protein L13e
		Psat2g045200: Unknown gene
		Psat2g044080: Domain of unknown function (DUF588)
		Psat2g044120: MAPEG family
		Psat2g044160: AAA domain
		Psat2g044320: AAA domain
		Psat2g044760: Endomembrane protein 70

Table S7. Genomic windows associated with putative breeding sweeps, sorted by decreasing XP-CLR score. Names and putative functions of the genes included in each window are reported.

Window coordinates	XP-CLR score	Gene name and function
7LG7_2000001_3000000	95,7499193	Psat7g002040: Protein of unknown function (DUF1295)
		Psat7g002080: PPR repeat family
		Psat7g002120: Golgin subfamily A member 5
		Psat7g002160: Ras family
		Psat7g002240: Prokaryotic membrane lipoprotein lipid attachment site profile.
		Psat7g002280: Unknown gene
		Psat7g002320: Unknown gene
		Psat7g002360: EF-hand domain pair
3LG5_18800001_19800000	51,03763679	Psat3g006760: Unknown gene
		Psat3g006800: GPCR-chaperone
		Psat3g006880: Unknown gene
		Psat3g006920: Pre-mRNA splicing factor component
		Psat3g006960: Unknown gene
		Psat3g006840: Unknown gene
5LG3_79600001_81200000	41,85524157	Psat5g044200: Unknown gene
		Psat5g044240: Protein of unknown function (DUF1645)
		Psat5g044280: RNA recognition motif. (a.k.a. RRM + RBD + or RNP domain)
		Psat5g044320: Unknown gene
		Psat5g044360: Protein of unknown function + DUF617
		Psat5g044400: Protein of unknown function + DUF617
		Psat5g044480: Unknown gene
		Psat5g044520: Protein of unknown function + DUF547
		Psat5g044560: Unknown gene
		Psat5g044600: Oligopeptide transporter OPT superfamily
		Psat5g044680: Down-regulated-in-metastasis protein
		Psat5g044720: Myb-like DNA-binding domain
		Psat5g044760: Phytocyanin domain profile.
		Psat5g044800: Unknown gene
		Psat5g044440: Uroporphyrinogen-III synthase HemD
		Psat5g044640: Unknown gene
4LG4_195600001_197000000	35,11670692	Psat4g104680: Receptor family ligand binding region
		Psat4g104720: CHD5-like protein
		Psat4g104800: Retrotransposon gag protein
		Psat4g104880: Unknown gene
		Psat4g104920: Myb-like DNA-binding domain
		Psat4g104960: Hydrolase activity + acting on acid anhydrides + catalyzing transmembrane movement of substances
		Psat4g105000: Domain of unknown function
		Psat4g105040: Mitochondrial ribosomal protein L27
		Psat4g105080: Histidine phosphatase superfamily + clade-1
		Psat4g105120: H+-transporting ATPase (proton pump) signature
		Psat4g105200: Nucleic acid binding protein NABP
		Psat4g105240: Unknown gene
		Psat4g105280: Unknown gene
		Psat4g105320: Unknown gene
		Psat4g105360: Exocyst complex + component Exoc1
		Psat4g105400: Protein kinase domain
		Psat4g105440: Retrotransposon gag protein
		Psat4g105480: Unknown gene
		Psat4g105520: Transmembrane amino acid transporter protein
		Psat4g105600: Unknown gene
		Psat4g105640: Reverse transcriptase-like
		Psat4g104600: RNB domain
		Psat4g104640: Ribonuclease 2-5A
		Psat4g104760: Unknown gene
3LG5_291800001_292800000	35,043179	Psat3g151000: 3-demethylubiquinone-9 3-methyltransferase
		Psat3g151080: Unknown gene
		Psat3g151160: Unknown gene
		Psat3g151200: Unknown gene
		Psat3g151280: Transferase activity + transferring acyl groups other than amino-acyl groups
		Psat3g151320: Unknown gene
		Psat3g151360: MazG-like family
		Psat3g151400: Myb-like DNA-binding domain
		Psat3g151600: Serine carboxypeptidase
		Psat3g151640: CAF1 family ribonuclease
		Psat3g151680: Unknown gene
		Psat3g151760: Intracellular membrane-bounded organelle
		Psat3g151800: Unknown gene
		Psat3g151120: Plasma membrane
		Psat3g151240: SOS response associated peptidase (SRAP)
		Psat3g151720: FAD dependent oxidoreductase
		Psat3g151560: Serine carboxypeptidase
2LG1_292400001_293600000	33,82953508	Psat2g111600: NB-ARC domain
		Psat2g111640: Unknown gene
		Psat2g111680: GDP-fucose protein O-fucosyltransferase
		Psat2g111720: NB-ARC domain
		Psat2g111760: Purine ribonucleotide binding
		Psat2g111800: NB-ARC domain
		Psat2g111840: Protein of unknown function + DUF393
		Psat2g111880: Tyrosine phosphatase family
		Psat2g112000: Unknown gene
		Psat2g112040: Unknown gene
		Psat2g112120: Protein kinase domain
		Psat2g112160: DnaJ domain
		Psat2g112200: Unknown gene
		Psat2g112280: Hydrolase activity + acting on acid anhydrides + in phosphorus-containing anhydrides
		Psat2g112320: Unknown gene
		Psat2g112360: Unknown gene
		Psat2g112400: Unknown gene
		Psat2g111960: NB-ARC domain
		Psat2g112080: Protein of unknown function (DUF1262)
		Psat2g112240: Plant transposase (Ptta/En/Spm family)
6LG2_168400001_169400000	31,27901779	Psat6g101800: Methylenetetrahydrofolate reductase
		Psat6g101840: Protein of unknown function (DUF3531)
		Psat6g101880: AAA domain
		Psat6g101960: SBP domain
		Psat6g102000: Protein of unknown function DUF3595
		Psat6g102040: Unknown gene
		Psat6g102120: Intracellular membrane-bounded organelle
		Psat6g102200: Prolyl oligopeptidase family
		Psat6g101920: BolA-like protein
		Psat6g102080: Cellular nucleobase + nucleoside + nucleotide and nucleic acid metabolic process
		Psat6g102160: Unknown gene
1LG6_344400001_345400000	29,06660409	Psat1g193000: GDSL-like Lipase/Acylhydrolase
		Psat1g193040: Hydrolase activity + acting on ester bonds
		Psat1g193080: Ribosomal S3Ae family
		Psat1g193120: K-box region
		Psat1g193160: Adipose-regulatory protein + Seipin
		Psat1g193240: Major intrinsic protein
		Psat1g193280: Unknown gene
		Psat1g193360: MAC/Perforin domain
		Psat1g193400: Unknown gene
		Psat1g193440: POT family
		Psat1g193480: Histone H2B signature
		Psat1g193560: Histone H2B signature
		Psat1g193600: Histone H2B signature
		Psat1g193640: Protein tyrosine kinase
		Psat1g193680: Unknown gene
		Psat1g193720: Protein of unknown function (DUF1264)
		Psat1g193760: Unknown gene
		Psat1g193800: Unknown gene
		Psat1g193840: Unknown gene
		Psat1g193920: HSF-type DNA-binding
		Psat1g193960: Unknown gene
		Psat1g193200: Putative adipose-regulatory protein (Seipin)
		Psat1g193320: Unknown gene
		Psat1g193520: Unknown gene
		Psat1g193880: Peptidase activity + acting on L-amino acid peptides
6LG2_38200001_39400000	28,9636044	Psat6g043000: Unknown gene
		Psat6g043040: Unknown gene
		Psat6g043080: ZPR1 zinc-finger domain
		Psat6g043120: Unknown gene
		Psat6g043160: Membrane transport protein
		Psat6g043200: PPR repeat
		Psat6g043240: Oxidoreductase activity + acting on peroxide as acceptor
		Psat6g043320: Pentatricopeptide repeat
		Psat6g043440: NUDIX domain
		Psat6g043480: Short chain dehydrogenase
		Psat6g043520: Unknown gene
		Psat6g043560: No apical meristem-associated C-terminal domain
		Psat6g043600: Unknown gene
		Psat6g043680: Unknown gene
		Psat6g043720: Protein kinase domain
		Psat6g043840: Wall-associated receptor kinase galacturonan-binding
		Psat6g043880: Protein kinase domain
		Psat6g043920: Unknown gene
		Psat6g044040: C2 domain
		Psat6g043400: Helicase conserved C-terminal domain
		Psat6g043640: GDP-fucose protein O-fucosyltransferase
		Psat6g043760: Protein tyrosine kinase
		Psat6g043800: Pathogenesis-related protein Bet v I family
		Psat6g043960: C2 domain
		Psat6g044080: C2 domain
3LG5_109400001_110600000	27,88599898	Psat3g050960: Pentatricopeptide repeat
		Psat3g051040: Unknown gene
		Psat3g051080: Unknown gene
		Psat3g051200: Response regulator receiver domain
		Psat3g051240: Response regulator receiver domain
		Psat3g051280: ABC-2 type transporter
		Psat3g051320: WD domain + G-beta repeat
		Psat3g051360: Response regulator receiver domain
		Psat3g051440: PPR repeat family
		Psat3g051480: Metallopeptidase family M24
		Psat3g051520: Malic enzyme + NAD binding domain
		Psat3g051560: Unknown gene
		Psat3g051600: Exostosin family
		Psat3g050800: Flavin-binding monooxygenase-like
		Psat3g050840: Unknown gene
		Psat3g051400: Aspartyl protease
7LG7_488600001_489600000	27,71898885	Psat7g260400: ABC transporter family E signature
		Psat7g260440: Unknown gene
		Psat7g260480: Unknown gene
		Psat7g260520: Kelch motif
		Psat7g260560: Galactose oxidase + central domain
		Psat7g260600: Unknown gene
		Psat7g260680: Core-2/I-Branching enzyme
		Psat7g260720: Unknown gene
		Psat7g260760: B-box zinc finger
		Psat7g260800: Unknown gene
		Psat7g260840: KH domain
		Psat7g260920: MULE transposase domain
		Psat7g260960: Glycosyl hydrolase family 47
		Psat7g261000: Cation efflux family
		Psat7g261040: Ribosomal L30 N-terminal domain
		Psat7g261120: Cation transporting ATPase + C-terminus
		Psat7g261160: Unknown gene
		Psat7g261200: GDP-fucose protein O-fucosyltransferase
		Psat7g261240: Zinc-binding in reverse transcriptase
		Psat7g261280: Adaptin N terminal region
		Psat7g261320: Exo70 exocyst complex subunit
		Psat7g261360: Leucine-rich repeat + cysteine-containing subtype
		Psat7g261400: CSN8/PSMD8/EIF3K family
		Psat7g261440: Leucine-rich repeat + cysteine-containing subtype
		Psat7g261480: Leucine-rich repeat + cysteine-containing subtype
		Psat7g261520: Leucine-rich repeat + cysteine-containing subtype
		Psat7g261560: Leucine-rich repeat + cysteine-containing subtype
		Psat7g261600: Leucine-rich repeat + cysteine-containing subtype
		Psat7g261640: CpeT/CpcT family (DUF1001)
		Psat7g261680: Zinc finger + Mcm10/DnaG-type
		Psat7g261720: F-box domain
		Psat7g261760: Leucine-rich repeat + cysteine-containing subtype
		Psat7g261800: Protein phosphatase 2C
		Psat7g261840: Unknown gene
		Psat7g261880: Protein kinase domain
		Psat7g261920: Xylanase inhibitor N-terminal
		Psat7g261960: Unknown gene
		Psat7g262000: Unknown gene
		Psat7g262040: Unknown gene
		Psat7g262080: Unknown gene
		Psat7g262120: Hydrolase activity + acting on carbon-nitrogen (but not peptide) bonds + in linear amides
		Psat7g262160: Unknown gene
		Psat7g262200: Unknown gene
		Psat7g262240: Unknown gene
		Psat7g262280: Prokaryotic membrane lipoprotein lipid attachment site profile.
		Psat7g262320: Unknown gene
		Psat7g262360: Unknown gene
		Psat7g262400: Unknown gene
		Psat7g262440: Unknown gene
		Psat7g262520: Transferase activity + transferring acyl groups other than amino-acyl groups
		Psat7g262560: Transferase family
		Psat7g262600: Unknown gene
		Psat7g262640: Prokaryotic membrane lipoprotein lipid attachment site profile.
		Psat7g262720: DYW family of nucleic acid deaminases
		Psat7g262760: Protein phosphatase 2C
		Psat7g262800: AP2 domain
		Psat7g260640: Hydrolase activity + acting on carbon-nitrogen (but not peptide) bonds + in linear amides
		Psat7g262880: CCT motif
		Psat7g261080: E1-E2 ATPase
		Psat7g262680: Prokaryotic membrane lipoprotein lipid attachment site profile.
6LG2_85200001_86200000	27,40985285	Psat6g068760: Unknown gene
		Psat6g068800: Unknown gene
		Psat6g068840: Tetratricopeptide repeat
		Psat6g068960: C2H2-type zinc finger
		Psat6g069000: C2H2-type zinc finger
		Psat6g069040: C2H2-type zinc finger
		Psat6g069080: Hydrolase activity + hydrolyzing O-glycosyl compounds
		Psat6g069160: Zinc finger + C3HC4 type (RING finger)
		Psat6g069200: Unknown gene
		Psat6g068880: Unknown gene
		Psat6g068920: Kinesin motor domain
		Psat6g069120: Prokaryotic membrane lipoprotein lipid attachment site profile.
6LG2_457000001_458800000	27,3805472	Psat6g227760: Trp-Asp (WD) repeats profile.
		Psat6g227800: Unknown gene
		Psat6g227840: Regulator of Vps4 activity in the MVB pathway
		Psat6g227880: Serine aminopeptidase + S33
		Psat6g227920: Unknown gene
		Psat6g227960: Unknown gene
		Psat6g228000: Unknown gene
		Psat6g228040: Unknown gene
		Psat6g228080: Unknown gene
		Psat6g227720: Trp-Asp (WD) repeats profile.
2LG1_89200001_90400000	26,89253474	Psat2g050560: AP2 domain
		Psat2g050600: Ubiquitin-conjugating enzyme
		Psat2g050640: Protein kinase domain
		Psat2g050680: Unknown gene
		Psat2g050720: Cytochrome P450
		Psat2g050800: Unknown gene
		Psat2g050840: Protein tyrosine kinase
		Psat2g050880: Unknown gene
		Psat2g050920: B3 DNA binding domain
		Psat2g050960: Unknown gene
		Psat2g051000: Unknown gene
		Psat2g051040: BZIP transcription factor
		Psat2g051080: Regulation of cellular nucleobase + nucleoside + nucleotide and nucleic acid metabolic process
		Psat2g051120: Regulation of cellular nucleobase + nucleoside + nucleotide and nucleic acid metabolic process
		Psat2g051160: Expansin signature
		Psat2g051280: Protein kinase domain
		Psat2g050760: NPH3 family
		Psat2g051200: Transferase activity + transferring phosphorus-containing groups
		Psat2g051240: Peptidase activity + acting on L-amino acid peptides
1LG6_304200001_305200000	25,09087503	Psat1g155240: 3-hydroxyacyl-CoA dehydrogenase + NAD binding domain
		Psat1g155280: Unknown gene
		Psat1g155320: Protein of unknown function (DUF295)
		Psat1g155360: Sugar efflux transporter for intercellular exchange
		Psat1g155400: LURP-one-related
		Psat1g155440: Prokaryotic membrane lipoprotein lipid attachment site profile.
		Psat1g155560: Unknown gene
		Psat1g155600: Unknown gene
		Psat1g155640: MatE
		Psat1g155520: Zinc-finger of the FCS-type + C2-C2
5LG3_224000001_225000000	24,72509194	Psat5g124880: Unknown gene
		Psat5g124960: Unknown gene
		Psat5g124920: Ras family
3LG5_251000001_252800000	23,82688807	Psat3g130120: Yippee zinc-binding/DNA-binding /Mis18 + centromere assembly
		Psat3g130160: Cellular nucleobase + nucleoside + nucleotide and nucleic acid metabolic process
		Psat3g130200: Yippee-like protein
		Psat3g130280: Transferase activity + transferring phosphorus-containing groups
		Psat3g130320: Unknown gene
		Psat3g130360: Alpha 1-4 glycosyltransferase conserved region
		Psat3g130400: Unknown gene
		Psat3g130560: Unknown gene
		Psat3g130600: S-adenosylmethionine-dependent methyltransferase activity
		Psat3g130480: TCP-1/cpn60 chaperonin family
		Psat3g130520: Transmembrane amino acid transporter protein
		Psat3g130440: PPR repeat family
1LG6_288600001_289600000	23,01292447	Psat1g145040: Unknown gene
		Psat1g145080: Unknown gene
		Psat1g145120: Unknown gene
		Psat1g145160: Unknown gene
		Psat1g145200: Unknown gene
		Psat1g145240: Unknown gene
		Psat1g145280: Unknown gene
		Psat1g145320: Unknown gene
		Psat1g145360: Unknown gene
		Psat1g145400: Unknown gene
		Psat1g145520: Pyridoxal phosphate-dependent transferase + major region + subdomain 1
		Psat1g145560: Transferase activity + transferring phosphorus-containing groups
		Psat1g145440: Rab-GTPase-TBC domain
		Psat1g145480: Plasma membrane
6LG2_172200001_174000000	22,46704659	Psat6g103840: Piwi domain
		Psat6g103920: Bacterial leader peptidase 1 (S26A) family signature
		Psat6g104040: Unknown gene
		Psat6g104080: Protein of unknown function (DUF4005)
[illegible]	[illegible]	[illegible]
		Psat6g000960: Unknown gene
		Psat6g001000: Alpha-amylase C-terminal beta-sheet domain
		Psat6g001080: Plasma membrane
		Psat6g001120: Complex 1 protein (LYR family)
		Psat6g001160: Complex 1 protein (LYR family)
		Psat6g001200: Protein kinase domain
		Psat6g001240: Protein kinases ATP-binding region signature.
		Psat6g001280: Reverse transcriptase (RNA-dependent DNA polymerase)
		Psat6g001320: Ring finger domain
		Psat6g001400: Expansin signature
		Psat6g001440: Unknown gene
		Psat6g001480: Protein of unknown function (DUF677)
		Psat6g001520: Protein of unknown function DUF677
		Psat6g001560: Protein of unknown function (DUF677)
		Psat6g001600: Alcohol dehydrogenase GroES-like domain
		Psat6g001680: GDSL-like Lipase/Acylhydrolase
		Psat6g001720: Unknown gene
		Psat6g001760: Ribosomal protein S3 + C-terminal domain
		Psat6g001800: GDSL-like Lipase/Acylhydrolase
		Psat6g001840: Hydrolase activity + acting on ester bonds
		Psat6g001880: Ligase activity + forming carbon-nitrogen bonds
		Psat6g001920: Unknown gene
		Psat6g001960: SRF-type transcription factor (DNA-binding and dimerisation domain)
		Psat6g002000: GDSL-like Lipase/Acylhydrolase
		Psat6g002040: GDSL-like Lipase/Acylhydrolase
		Psat6g002080: GDSL-like Lipase/Acylhydrolase
		Psat6g002120: Unknown gene
		Psat6g002160: GDSL-like Lipase/Acylhydrolase
		Psat6g002200: Intracellular membrane-bounded organelle
		Psat6g002240: Unknown gene
		Psat6g002320: Unknown gene
		Psat6g001360: LAGLIDADG DNA endonuclease family
		Psat6g001640: La domain
		Psat6g002280: Unknown gene
7LG7_455200001_456200000	15,89445968	Psat7g226320: Unknown gene
		Psat7g226400: Sad1 / UNC-like C-terminal
		Psat7g226480: Casein Kinase 2 substrate
		Psat7g226520: EMG1/NEP1 methyltransferase
		Psat7g226560: Ribosomal L18 of archaea + bacteria + mitoch. and chloroplast
		Psat7g226600: Unknown gene
		Psat7g226680: Unknown gene
		Psat7g226720: AIG1 family
		Psat7g226760: AIG1 family
		Psat7g226800: Unknown gene
		Psat7g226840: Plant phospholipid transfer protein signature
		Psat7g226360: Dynamin family
5LG3_183600001_185000000	15,84383705	Psat5g101480: Unknown gene
		Psat5g101520: Unknown gene
		Psat5g101600: Unknown gene
		Psat5g101640: Unknown gene
		Psat5g101680: Unknown gene
		Psat5g101720: 2OG-Fe(II) oxygenase superfamily
		Psat5g101760: Multicopper oxidase
		Psat5g101800: O-methyltransferase
		Psat5g101840: Ubiquitin carboxyl-terminal hydrolase
		Psat5g101880: Unknown gene
		Psat5g101920: Unknown gene
		Psat5g101960: Unknown gene
		Psat5g102000: Unknown gene
		Psat5g102040: Multicopper oxidase
		Psat5g102120: Sec23/Sec24 trunk domain
		Psat5g102160: Regulation of small GTPase mediated signal transduction
		Psat5g102200: Rab-GTPase-TBC domain
		Psat5g102240: Sodium/hydrogen exchanger family
		Psat5g102280: Unknown gene
		Psat5g102320: TAFII55 protein conserved region
		Psat5g102360: Response to chemical stimulus
		Psat5g102400: Unknown gene
		Psat5g102440: Unknown gene
		Psat5g102520: Cystatin domain
		Psat5g102560: S-adenosylmethionine-dependent methyltransferase activity
		Psat5g102600: Cystatin domain
		Psat5g102640: Unknown gene
		Psat5g102680: Acetyltransferase (GNAT) domain
		Psat5g102720: Molecular chaperone + heat shock protein + Hsp40 + DnaJ
		Psat5g101560: Unknown gene
		Psat5g102080: Calcineurin-like phosphoesterase
		Psat5g102480: Unknown gene
6LG2_28800001_29800000	15,72451246	Psat6g035920: Macro domain
		Psat6g035960: Mut7-C RNAse domain
		Psat6g036040: Unknown gene
		Psat6g036160: Major sigma-70 factor signature
		Psat6g036200: Transmembrane amino acid transporter protein
		Psat6g036280: Unknown gene
		Psat6g036320: F-box associated
		Psat6g036360: Unknown gene
		Psat6g036400: Unknown gene
		Psat6g036440: Unknown gene
		Psat6g036480: Ribosomal L28 family
		Psat6g036520: Unknown gene
		Psat6g036560: OPT oligopeptide transporter protein
		Psat6g036600: OPT oligopeptide transporter protein
		Psat6g036640: Rab-GTPase-TBC domain
		Psat6g036680: PPR repeat family
		Psat6g036720: DYW family of nucleic acid deaminases
		Psat6g035840: WD domain + G-beta repeat
		Psat6g036000: PHD-finger
		Psat6g036080: Haloacid dehalogenase-like hydrolase
		Psat6g036120: Early nodulin 93 ENOD93 protein
		Psat6g035880: TLC ATP/ADP transporter
6LG2_428600001_430000000	15,65987737	Psat6g215120: Unknown gene
		Psat6g215200: Unknown gene
		Psat6g215280: Malic enzyme + N-terminal domain
		Psat6g215320: Clp protease
		Psat6g215440: Unknown gene
		Psat6g215480: Unknown gene
		Psat6g215520: Protein of unknown function DUF260
		Psat6g215560: Family of unknown function (DUF566)
		Psat6g215600: BED zinc finger
		Psat6g215240: NPH3 family
		Psat6g215360: Transition metal ion binding
		Psat6g215400: Helix-loop-helix DNA-binding domain
3LG5_255800001_257600000	15,61330953	Psat3g130920: Unknown gene
		Psat3g130960: Protein of unknown function (DUF740)
		Psat3g131000: Zinc finger C2H2 type domain signature.
		Psat3g131080: Transmembrane amino acid transporter protein
		Psat3g131120: Intracellular membrane-bounded organelle
		Psat3g131160: PPR repeat family
		Psat3g131040: Haloacid dehalogenase-like hydrolase
6LG2_108800001_109800000	15,58619315	Psat6g080160: Unknown gene
		Psat6g080200: Gag-polypeptide of LTR copia-type
		Psat6g080240: Unknown gene
		Psat6g080280: Unknown gene
		Psat6g080320: Unknown gene
		Psat6g080360: Calcineurin-like phosphoesterase
		Psat6g080400: Unknown gene
		Psat6g080480: Unknown gene
		Psat6g080520: Unknown gene
		Psat6g080560: IQ motif profile.
		Psat6g080600: Glycosyltransferase like family 2
		Psat6g080440: F-box domain
2LG1_379000001_380000000	15,58220878	Psat2g148600: Unknown gene
		Psat2g148640: Unknown gene
		Psat2g148680: TPL-binding domain in jasmonate signalling
		Psat2g148720: Unknown gene
		Psat2g148760: Ring finger domain
		Psat2g148800: Unknown gene
		Psat2g148880: Unknown gene
		Psat2g148920: PPR repeat family
		Psat2g148960: Rpp14/Pop5 family
		Psat2g149080: Binding
		Psat2g149120: Sec63 Brl domain
		Psat2g149160: RWP-RK domain
		Psat2g149200: Lipoxygenase
		Psat2g149240: Protein kinase domain
		Psat2g149280: SacI homology domain
4LG4_74200001_75200000	15,43262596	Psat4g042360: Unknown gene
		Psat4g042400: Unknown gene
		Psat4g042440: Unknown gene
		Psat4g042520: Protein phosphatase 2A regulatory B subunit (B56 family)
		Psat4g042560: Polynucleotidyl transferase + ribonuclease H fold
		Psat4g042600: Unknown gene
		Psat4g042640: Unknown gene
		Psat4g042680: Unknown gene
		Psat4g042720: Acyl-ACP thioesterase
		Psat4g042760: Unknown gene
		Psat4g042840: Unknown gene
		Psat4g042480: Integral membrane protein DUF92
		Psat4g042800: Domain of unknown function (DUF4339)
1LG6_14800001_15800000	15,31404869	Psat1g011760: Skp1 family + dimerisation domain
		Psat1g011800: Armadillo/plakoglobin ARM repeat profile.
		Psat1g011840: Transferase activity + transferring glycosyl groups
		Psat1g011880: Skp1 family + dimerisation domain
		Psat1g011920: Unknown gene
		Psat1g011960: Protein tyrosine kinase
		Psat1g012000: Zinc finger + C3HC4 type (RING finger)
		Psat1g012080: Major facilitator superfamily + general substrate transporter
		Psat1g012120: Glycosyl transferases group 1
		Psat1g012160: Gag-polypeptide of LTR copia-type
		Psat1g012200: Elongation factor Tu GTP binding domain
		Psat1g012280: Unknown gene
		Psat1g012320: Nodule-specific GRP repeat
		Psat1g012360: Nodule-specific GRP repeat
		Psat1g012400: 2OG-Fe(II) oxygenase superfamily
		Psat1g012440: 2OG-Fe(II) oxygenase superfamily
		Psat1g012480: Unknown gene
		Psat1g012520: Unknown gene
		Psat1g012560: Elongation factor Tu GTP binding domain
		Psat1g012600: Hydrolase activity + acting on acid anhydrides + in phosphorus-containing anhydrides
		Psat1g012640: Galactose oxidase + central domain
		Psat1g012680: No apical meristem (NAM) protein
		Psat1g012040: HORMA domain
4LG4_296000001_297800000	15,16023537	Psat4g150440: Unknown gene
		Psat4g150520: ATPase family associated with various cellular activities (AAA)
		Psat4g150600: Ribosomal protein S28e
		Psat4g150640: Sin3 associated polypeptide p18 (SAP18)
		Psat4g150720: D-isomer specific 2-hydroxyacid dehydrogenase + NAD binding domain
		Psat4g150760: D-isomer specific 2-hydroxyacid dehydrogenase + NAD binding domain
		Psat4g150800: D-isomer specific 2-hydroxyacid dehydrogenase + NAD binding domain
		Psat4g150840: Unknown gene
		Psat4g150880: Cid1 family poly A polymerase
		Psat4g150920: C2 domain
		Psat4g151000: Cellular amino acid and derivative metabolic process
		Psat4g151080: F-box associated interaction domain
		Psat4g151160: Unknown gene
		Psat4g151240: Phospholipase D C terminal
		Psat4g151280: TCP family transcription factor
		Psat4g151320: Putative S-adenosyl-L-methionine-dependent methyltransferase
		Psat4g151360: Phloem protein 2
		Psat4g151400: Anion-transporting ATPase
		Psat4g151440: Urm1 (Ubiquitin related modifier)
		Psat4g151520: WD domain + G-beta repeat
		Psat4g150480: Glycosyl transferase family 8
		Psat4g150960: Phospholipase D Active site motif
		Psat4g151040: F-box associated
		Psat4g151120: Subtilase family
		Psat4g151480: G protein beta WD-40 repeat signature
6LG2_436400001_437600000	15,05832547	Psat6g220760: Unknown gene
		Psat6g220800: Unknown gene
		Psat6g220840: Unknown gene
		Psat6g220880: Unknown gene
		Psat6g220920: Transcription factor + FAR1-related
		Psat6g220960: Protein tyrosine kinase
		Psat6g221000: Unknown gene
		Psat6g221040: Unknown gene
		Psat6g221080: PPR repeat
		Psat6g221120: Reverse transcriptase-like
		Psat6g221200: Unknown gene
		Psat6g221240: Unknown gene
		Psat6g221280: Unknown gene
		Psat6g221160: MatE
6LG2_146400001_147400000	14,64486862	Psat6g092640: Diphthamide synthesis + DPH1/DHP2
		Psat6g092680: Plant phosphoribosyltransferase C-terminal
		Psat6g092720: Gag-polyprotein putative aspartyl protease
		Psat6g092840: Uncharacterised conserved protein
		Psat6g092880: Unknown gene
		Psat6g092920: CCAAT-binding transcription factor (CBF-B/NF-YA) subunit B
		Psat6g093000: Unknown gene
		Psat6g093040: ABC-2 type transporter
		Psat6g093080: Pentatricopeptide repeat
[illegible]	[illegible]	[illegible]
